# Distinct Origin of Claudin7 in Early Tumor Endosomes Affects Exosome Assembly

**DOI:** 10.7150/ijbs.35347

**Published:** 2019-08-19

**Authors:** Daisuke Kyuno, Nathalie Bauer, Martina Schnölzer, Jan Provaznik, Eduard Ryschich, Thilo Hackert, Margot Zöller

**Affiliations:** 1 Department of General, Visceral and Transplantation Surgery, University of Heidelberg, Germany.; 2 Department of Surgery, Surgical Oncology and Science, Sapporo Medical University, Sapporo, Japan.; 3 Functional Proteome Analysis, DKFZ, Heidelberg, Germany.; 4 Gene Core Center, EMBL, Heidelberg, Germany.

**Keywords:** Claudin7, cancer-initiating cells, tumor exosomes, microvesicle proteome, microvesicle miRNA, gastrointestinal cancer

## Abstract

Microvesicles are the body's most powerful intercellular communication system and cancer-initiating cell microvesicles (CIC-TEX) reprogram Non-CIC towards fortified malignancy. Claudin7, a CIC-biomarker in gastrointestinal tumors, is recovered in TEX. Recent evidence suggesting individual cells delivering distinct microvesicles became of particular interest for claudin7, which is part of tight junctions (TJ) and glycolipid-enriched membrane domains (GEM), GEM-located claudin7 is palmitoylated. This offered the unique possibility of exploring the contribution of a CIC marker and its origin from distinct membrane domains on CIC-TEX biogenesis and activities. Proteome and miRNA analysis of wild-type, claudin7-knockdown and a rescue with claudin7 harboring a mutated palmitoylation site (mP) of a rat pancreatic and a human colon cancer line uncovered significant, only partly overlapping contributions of palmitoylated and non-palmitoylated claudin7 to TEX composition. Palmitoylated claudin7 facilitates GEM-integrated plasma membrane and associated signaling molecule recruitment; non-palmitoylated claudin7 supports recruitment of trafficking components, proteins engaged in fatty acid metabolism and TJ proteins into TEX. Claudin7mP also assists TEX recovery of selected miRNA. Thus, distinctly located claudin7 affects CIC-TEX composition and TJ-derived cld7 might play a unique role in equipping CIC-TEX with transporters and lipid metabolism-regulating molecules, awareness of distinct TEX populations being crucial facing therapeutic translation.

## Introduction

Cancer-initiating cells (CIC) are the major obstacle in curative treatment after tumor spread from the primary location 1. This minor subpopulation in the mass of the primary tumor accounts for drug and radiation resistance 2, tumor cell dissemination and metastatic growth 3. CIC fulfill these tasks via exosomes/microvesicles (TEX) 4,5, which reprogram host cells and Non-CIC 6. Strong evidence for a contribution of CIC markers in TEX biogenesis 7 is of particular interest for claudin7 (cld7), a pancreatic and colorectal cancer (PaCa, CoCa) CIC marker 8,9, which is recovered in distinct TEX subpopulations 10.

Claudins are four-pass membrane proteins. They are essential for the formation of tight junctions (TJ) 11,12. Expression of many clds, including cld7 is vital. Cld7-knockout (ko) mice show a lethal destruction of the intestine within 10 days after birth 13,14, which is suggested to rely on a failure to associate with integrins and to upregulate metalloproteinase-3 expression 13 or on enhanced paracellular flux of an inflammation-initiating major bacterial product across the TJ of the colon 14. Notably, claudins are targets of and become phosphorylated by protein kinase A and myosin light chain kinase. Phosphorylated clds are not integrated into TJ, which leads to lost epithelial cell polarization 15-17, internalization and, at least, a partial recruitment into microvesicles (MV) 18. Besides TJ remodeling-promoted cld internalizations 19,20, membrane-anchored clds are also recovered outside of TJ in glycolipid-enriched membrane domains (GEM), where cld7 palmitoylation is a prerequisite 21,22. Within GEM, palmitoylated cld7 associates with EpCAM and tetraspanins. GEM function as a scaffold for attachment of signal transduction, cytosolic signaling and cytoskeleton linker molecules 23,24. Due to the lipid composition, GEM are prone for internalization. Internalized GEM are engaged in exosome biogenesis 25. Accordingly, cld7 is recovered in two distinct TEX populations, derived from GEM, where it is EpCAM-associated or from vesicles harboring “TJ-excluded” cld7 10.

Exosomes are a subpopulation of small 40-130nm microvesicles. Many cells and abundantly tumor cells deliver exosomes 7. Exosome biogenesis starts with the formation of early endosomes (EE), which derive from the trans-Golgi network or internalized membrane microdomains, including TJ, GEM, clathrin-coated pits and cholesterol- and ceramide-rich membrane domains 26. EE are guided to multivesicular bodies (MVB), the transport machinery varying for the different types of EE 27. During inward budding of these intraluminal vesicles (ILV) into MVB, vesicles are loaded with proteins, coding and noncoding RNA and DNA by non-random processes 28. After transport to the cell membrane, MVB fuse with the plasma membrane, the released ILV are called exosomes 26,27. Exosome disperse in the body 29. They bind to and can become ingested by targets, which express appropriate ligands 30. Exosome components are function competent 31, binding and the transferred messages promoting target modulation 32,33.

We here asked whether palmitoylated and possibly TJ-derived, non-palmitoylated cld7 contribute distinctly to the TEX composition and ILV loading. We approached the question using the rat PaCa ASML and the human CoCa SW948 lines as CIC-TEX donors. A cld7-knockdown (kd) in these lines results in a loss of CIC features 8,9. To decipher cld7 location-dependent activities, ASML-cld7kd cells were transfected with cld7 harboring a mutated palmitoylation site (cld7mP) 21,22.

## Results

CIC-TEX promote metastasis by altering host cells and non-CIC 4,5,7. In gastrointestinal cancer a cld7kd severely affects tumor progression 8,30, in vitro studies suggesting regain of metastatic features by coculturing cld7kd tumor cells with cld7-competent CIC-TEX 9,22. We here explored in rat pancreatic ASML and human colorectal SW948 tumor cells the impact of distinctly located cld7 on the proteome and miRNA TEX profile.

### The impact of palmitoylated and non-palmitoylated cld7 on the proteome profile of cells and TEX

Cld7 is detected in the cell membrane at two different locations, in TJ and GEM 10,21,22. TJ-located cld7 is non-phosphorylated and non-palmitoylated 21. GEM-located cld7 is palmitoylated and associated with EpCAM and tetraspanins 22. TJ- and GEM-located cld7 become internalized, TJ-located cld7 upon phosphorylation 16 and GEM-located cld7 as part of the GEM-internalization complex 25. At least part of internalized cld7 is recovered in TEX, being delivered either from the apical membrane, where cld7 is EpCAM-associated or from the basal membrane, where TEX lack EpCAM 10. This information was used comparing the protein profile of wt, cld7kd and cld7mP cells and TEX, the cld7mP rescue allowing differentiating between GEM versus Non-GEM derived cld7.

**Figure 1:**
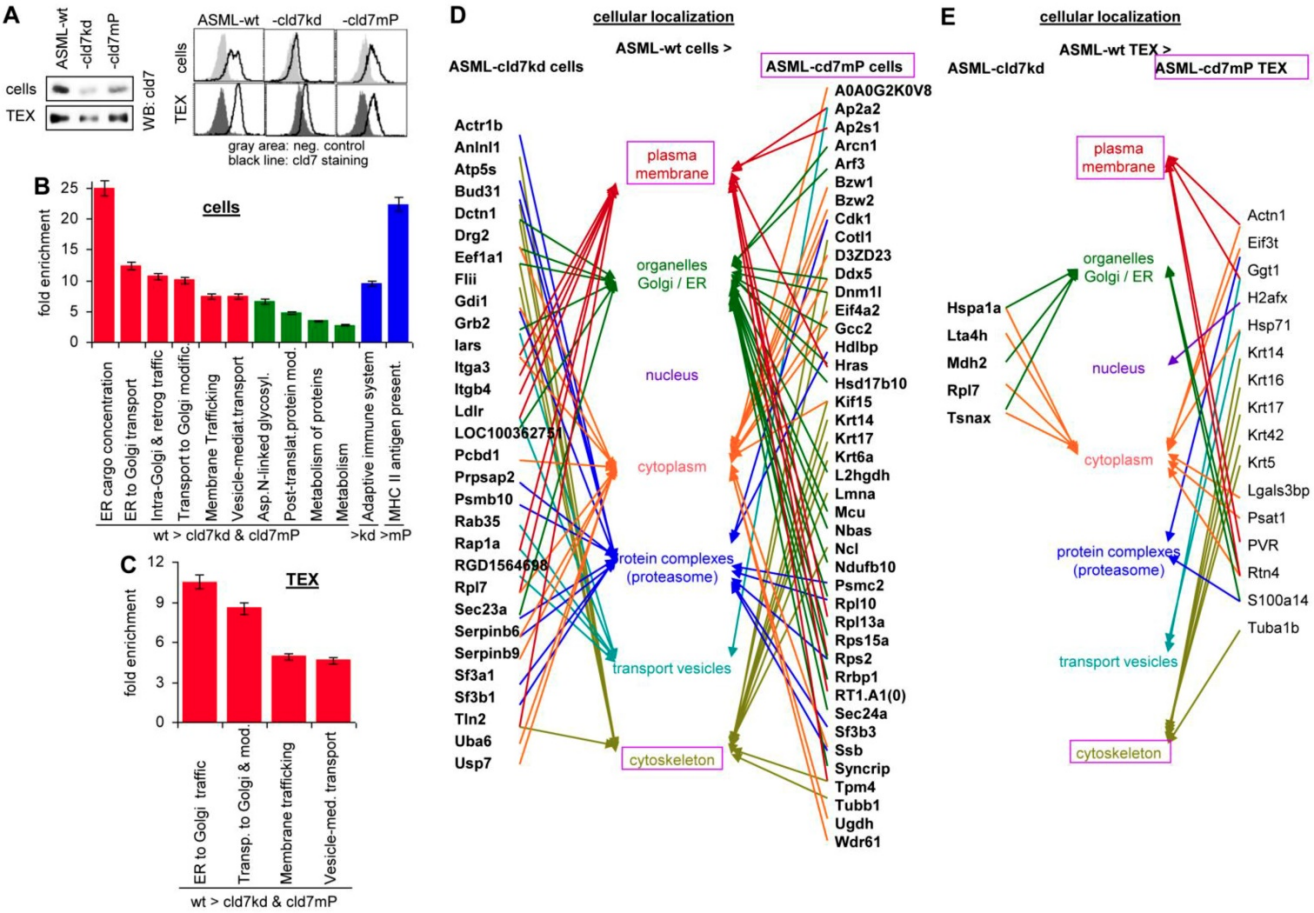
** The impact of cld7 on the protein profile of pancreatic cancer cells and TEX**. (A) Representative examples of WB and flow-cytometry overlays of cld7 expression in ASML-wt, -cld7kd and -cld7mP cells and TEX. (B-E) NanoLC-ESI-MS/MS proteome analysis of ASML-wt, -cld7kd and -cld7mP cells and TEX. Distinctly recovered proteins were evaluated in (B) cells and (C) TEX for the indicated enrichments according to KEGG; (D,E) Panther/Reactome analysis of cell compartment localization of proteins recovered at a higher level in ASML-wt than -cld7kd and -cld7mP cells and TEX; (D,E) full names of synonyms in Table S6. The strong overrepresentation of transporter and vesicle biogenesis-associated molecules in wt cells and TEX indicates engagement of cld7.

Western blot (WB) and flow-cytometry of ASML cells and TEX confirmed the efficacy of the cld7kd and the partial rescue in cld7mP cells and TEX, rescued cld7mP being non-palmitoylated 22 (Fig. 1A). The proteome profile was elaborated by nanoLC-ESI-MS/MS on an LTQ orbitrap. A total of 1723-1744 proteins were recovered in ASML-wild type (wt), -cld7kd and -cld7mP cells. From these, 119 proteins were preferentially recovered in wt compared to cld7kd and cld7mP cells, 30 preferentially compared to cld7kd cells and 43 compared to cld7mP cells (Table S1A-S1C). Panther pathway molecular function classification revealed no obvious differences between wt, cld7kd and cld7mP cells, but structural molecules were enriched in wt compared to cld7kd cells. Reactome analysis of biological processes indicated a high enrichment of proteins engaged in vesicle biogenesis and transport (Fig. S1A, Fig. 1B, Table S1D). The protein profile of ASML-wt, -cld7kd and -cld7mP TEX (Table S1E-S1G) provided similar results; no significant differences in the overall recovery of proteins and the molecular functions, but structural molecule and transporter enrichment in wt compared to cld7kd and cld7mP TEX (Fig. S1B, Table S1H). According to Reactome pathway analysis all proteins enriched in wt TEX are involved in transport and trafficking (Fig. 1C). It should be stressed that the reduced recovery of transporters in cells and TEX is selective for the cld7kd/cld7mP, not being seen in e.g. ASML-CD44v6kd or ASML-Tspan8kd cells 34. The strong link of wt cell- and TEX-enriched proteins in vesicle trafficking prompted us evaluating the cellular localization of distinctly expressed proteins. The majority of proteins more abundant in wt than cld7kd cells is located in the plasma membrane and transport vesicles. Proteins more abundant in ASML cells than ASML-cld7mP cells are located in organelles or associated with the cytoskeleton, indicating differences in palmitoylated cld7 and cld7mP trafficking (Fig. 1D). Too few proteins were distinctly recovered in wt and cld7kd TEX to judge on differences in the cellular origin. However, in TEX derived from cld7mP cells, the linkage to the cytoskeleton was maintained (Fig. 1E).

Proteins displaying reduced expression in ASML-cld7kd and -cld7mP cells and TEX cover a wide range of functions. In ASML-cld7kd and -cld7mP cells, mostly metabolism- and trafficking/transport-engaged proteins are reduced. In cld7mP cells, a reduction in trafficking/transport is dominating (Fig. S1C). In TEX, transcription/translation- and signaling-engaged proteins are most frequently reduced in cld7kd and cld7mP TEX. A reduction in structural molecule expression is more frequent in cld7mP than cld7kd TEX (Fig.S1D). However, differences between GEM-associated cld7 and cld7mP are less pronounced in TEX than cells, also demonstrated by flow-cytometry. Differences in several GEM-located protein expression, like Tspan8-associated CD29 and CD104 are waved or reduced (CD49f) in TEX, whereas CD166 is reduced in cld7kd and cld7mP TEX, but only in cld7kd cells (Fig. S1E).

**Figure 2:**
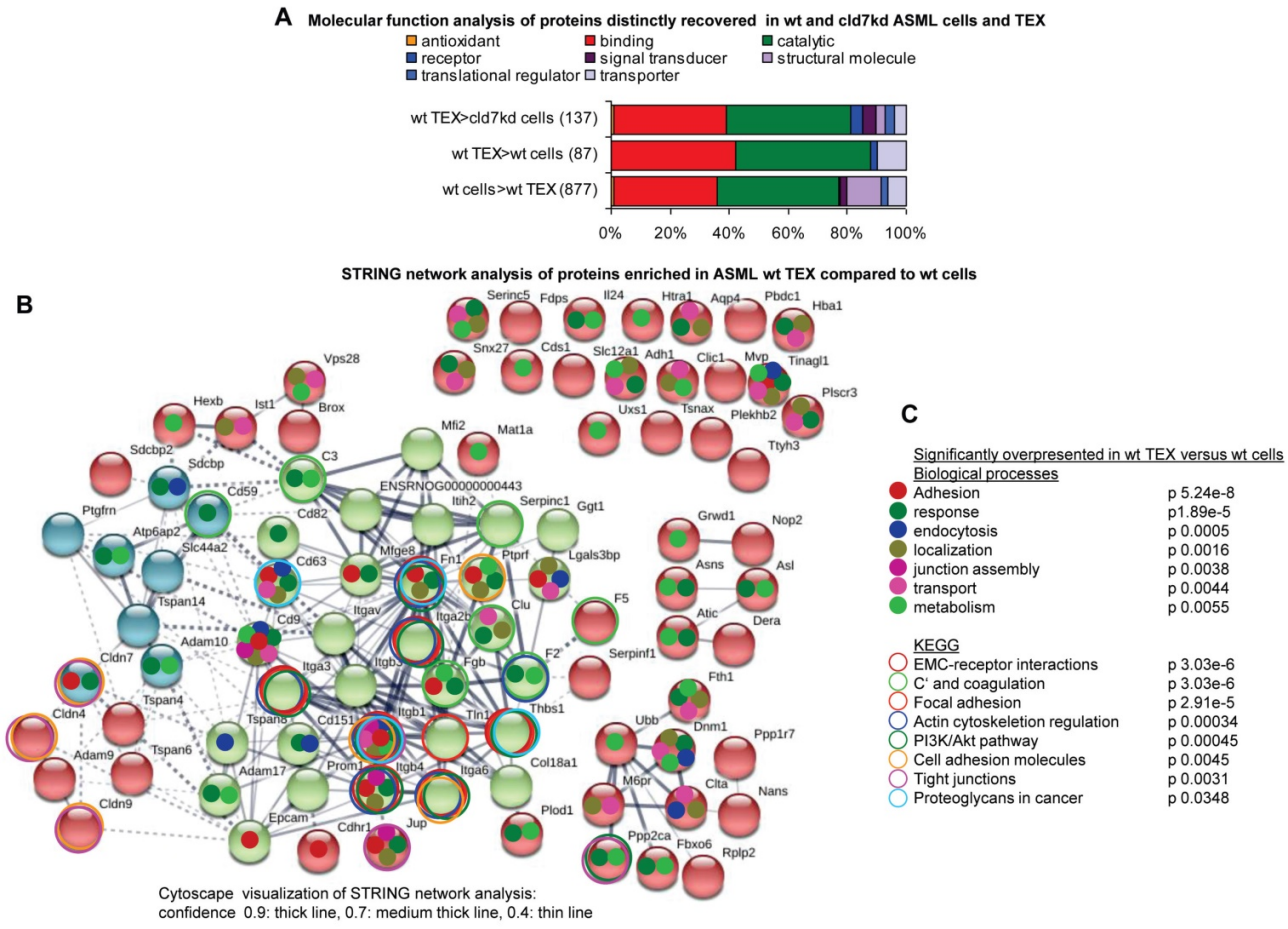
** Functional annotation and connectivity of proteins distinctly recovered in ASML cells versus TEX**. (A) Panther/Reactome analysis of molecular functions of proteins expressed at a higher level in wt cells than TEX or wt TEX than wt and cld7kd cells; (B) STRING network analysis of proteins expressed at a higher level in wt TEX than cells; thickness of the connecting lines correspond to the confidence level; full names of synonyms in Table S6. (C) Overrepresentation in wt TEX compared to cells according to biological processes and KEGG analysis. Overrepresentation according to biological function is indicated in (B) by a color-matching dot and according to KEGG analysis by a color-matching ring. Overrepresentation analysis indicated TEX being derived from TEM and TJ. Though connectivity of TEX-enriched proteins was stronger for GEM-derived/palmitoylated cld7 (integrins, tetraspanins), the overrepresentation of several clds strengthens cld7 recruitment from different cellular compartments.

In concern about the impact of CIC-TEX on cld7kd cells, proteins selectively enriched in ASML-wt TEX are particularly important. ASML-wt TEX showed loss or reduced recovery of 887 proteins compared to wt cells; 87 proteins were enriched in wt TEX compared to wt cells and 137 proteins compared to cld7kd cells (Table S1I-S1K, Fig. 2A). STRING network analysis revealed some connectivity of proteins upregulated in ASML-wt TEX compared to cells. Integrins and tetraspanins forming a core centre point towards the dominance of GEM-derived TEX. However, junction associated proteins like clds as well as transporters are also enriched in TEX compared to cells (Fig. 2B). Searching for significantly overrepresented nodes according to biological processes revealed that proteins engaged in adhesion, response to stimuli, endocytosis, localization in the cell, junction assembly, transport and metabolism also are enriched in TEX compared to cells. KEGG analysis indicated a considerable number of proteins overrepresented in TEX being engaged in ECM-receptor interactions, complement activation and coagulation, focal adhesion, actin cytoskeleton regulation, cell adhesion, TJ formation, proteoglycan regulation and the PI3K/Akt pathway, which all are cancer-related activities (Fig. 2C). However, tetraspanins and associated molecules, complement components and MFGE8 (Lactadherin), to name a few; are known to be enriched in exosomes 39, suggesting cld7-independent TEX enrichment. Finally, we searched for the functions of proteins enriched in TEX compared to cld7kd cells, as the potential impact of CIC-TEX on non-metastatic cld7kd cells is of particular interest. Mostly signaling molecules, but also proteins contributing to transcription/translation, trafficking/transport and metabolism are enriched in wt TEX compared to cld7kd cells (Fig. S1F).

To confirm the validity of cld7 contributing to TEX biogenesis and a suggested impact of wt TEX on cld7kd cells the proteome analysis was repeated with wt and cld7kd SW948 cells and TEX (Table S2A-S2D). Similar to ASML cells/TEX, the cld7kd was more efficient in cells than TEX, cld7 recovery being reduced in SW948-cld7kd cells by >90% and in TEX by >60% (Fig. S2A), which indicates a selective enrichment of cld7 in TEX. In wt compared to cld7kd cells, 143 proteins were recovered at a higher level. Otherwise, 103 proteins were higher in cld7kd than wt cells (Fig. S2B). In SW948-wt and -cld7kd TEX 824 and 882, respectively, proteins were recovered, 51 being higher in wt TEX and 79 higher in cld7kd TEX (Fig. S2C). Expectedly, protein recovery differed between cells and TEX (Table S2E-S2G, Fig. S2D). The majority of proteins reduced in SW948-cld7kd cells is engaged in transcription/translation and signal transduction, reduced recovery in cld7kd cells being confirmed for selected proteins by flow-cytometry (Fig. S2E, S2F). Distinct to cells, but similar to ASML TEX, mostly signal transduction proteins differed between wt and cld7kd TEX. This accounted for proteins with higher and lower expression in cld7kd TEX (Fig. S2G). Reactome analysis indicated overrepresentation predominantly of signaling and transporter/trafficking molecules in wt TEX compared to wt and cld7kd cells, many of these molecules being constitutive Exo components like tetraspanins and associated integrins, FPRP (Prostaglandin F2 receptor inhibitor), ADAM (Disintegrin and metalloproteinase domain-containing protein)10, DPP (Dipeptidyl peptidase)4 and EpCAM, MFGE8, TSG (Tumor-susceptibility gene)101, Annexins, and the majority of proteins involved in vesicle trafficking and release, like Rab35, Rab6B, several solute carrier (SLC) transporters and vacuolar sorting proteins (VSP). High recovery of Ephrins and Ephrin receptors in TEX also was repeatedly described 40. Notably, some junction components are also enriched in TEX compared to cells, e.g. several cld, JAM (Junctional adhesion molecule)1 and JUP (Junction plakoglobin) (Fig. S2H). Wt TEX proteins selectively exceeding the level in wt cells are predominantly engaged in transcription/translation. This differed significantly from the 24 proteins selectively higher in wt TEX than cld7kd cells, signaling molecules including tetraspanins and trafficking/transport molecules being mostly affected (Fig. S2I). Importantly, exemplified by flow-cytometry, the expression level of some proteins strongly differed between TEX and cells (Fig. S2J).

Briefly, signal transducing and transporter molecules are highly enriched in TEX compared to cells. However, differences due to palmitoylation-competent versus palmitoylation-deficient cld7 are weaker in TEX than cells.

### Selective contributions of GEM- versus non-GEM located cld7 to protein recruitment into TEX

To pursue the question of a cld7-selective contribution to TEX loading, ASML-wt and -cld7mP cells and TEX were precipitated with anti-cld7 comparing the pattern of coimmunoprecipitating molecules. According to the mild lysis condition, the precipitates include loosely attached molecules. Precipitates were lysed, separated by SDS gel and analyzed by mass spectrometry (nanoLC-ESI-MS/MS on an LTQ orbitrap).

**Figure 3:**
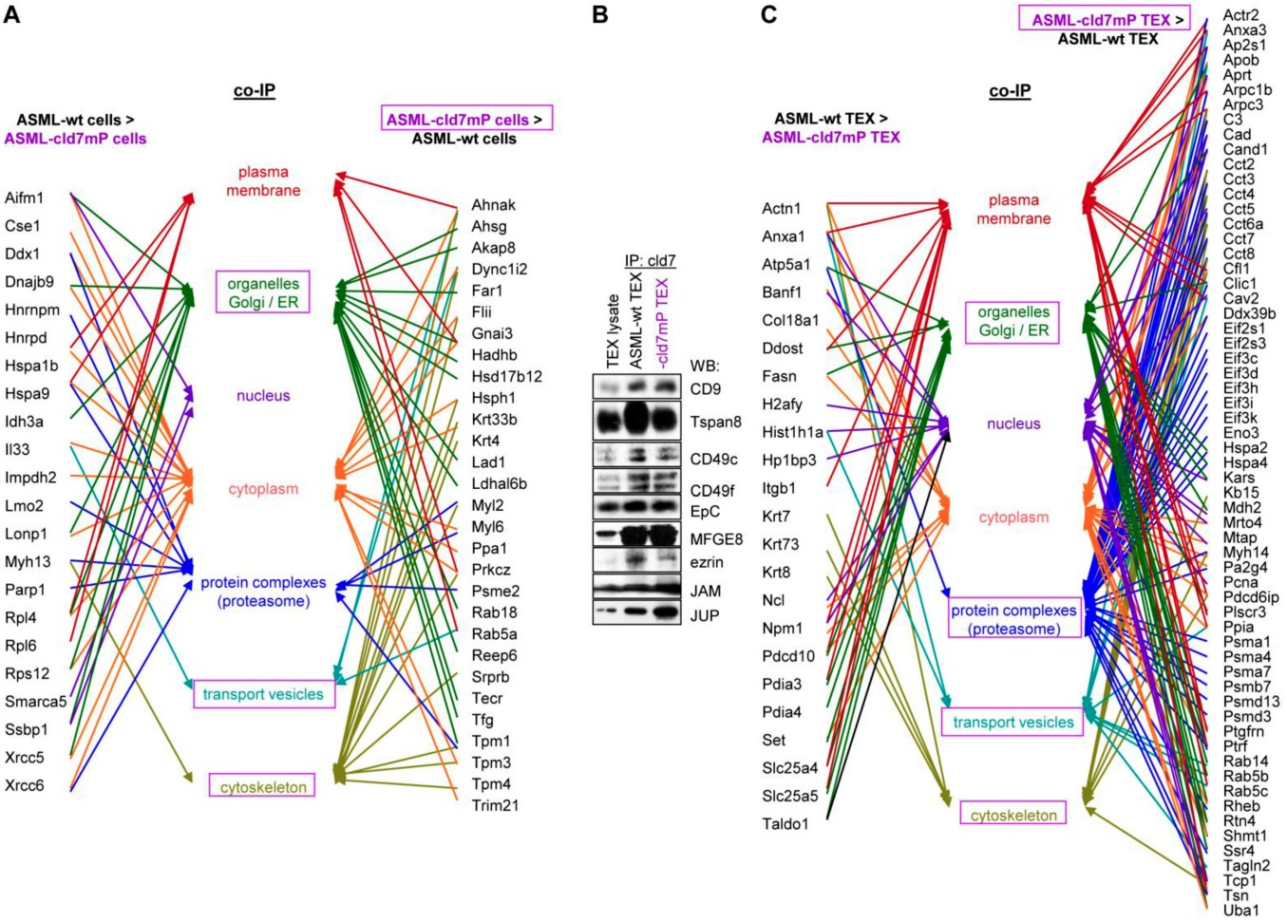
** Cellular locations of claudin7 coimmunoprecipitating proteins**. Lysates of ASML-wt and -cld7mP reconstituted cells and TEX were precipitated with anti-cld7. Precipitates were subjected to NanoLC-ESI-MS/MS proteome analysis. (A) Cellular location of coimmunoprecipitating molecules depending on cld7 palmitoylation in cells; (B) ASML-wt and -cld7mP TEX were precipitated with anti-cld7 and blotted with the indicated antibodies; the precipitates and the lysate control of ASML-wt cells are shown; (C) TEX recovery depending on cld7 palmitoylation of coimmunoprecipitating molecules according to the location in cells; (A,C) preferential locations on cld7mP cells and TEX coimmunoprecipitates are framed in violet; full names of synonyms in Table S6. The difference in cld7- versus cld7mP-coimmunoprecipitating molecules in TEX suggests a broader range of proteins being recruited towards ILV by non-palmitoylated than palmitoylated cld7, which is supported by the location of the coimmunoprecipitating molecules in organelles, protein complexes, and transport vesicles. The interpretation was corroborated by NanoLC-ESI-MS/MS proteome analysis of anti-EpCAM precipitates (data not shown).

First to note, more proteins coimmunoprecipitate with cld7 in ASML-wt TEX (213) than cells (110). This likely depends on the TEX membrane lipid composition that supports palmitoylated or myristoylated protein attachment (Fig. S3A). In ASML-wt cells, 76 molecules and in cld7mP cells 71 molecules coimmunoprecipitated with anti-cld7 (Fig. S3B, Table S3A-S3C). Instead, in ASML-wt TEX 67 proteins and in ASML-cld7mP TEX 117 proteins coimmunoprecipitated with cld7, indicating a shift towards TEX delivery in cld7mP cells. Furthermore, adhesion molecules were reduced and structural molecules were overrepresented in cld7-coimmunoprecipitates of wt TEX compared to cells (Fig. S3B, S3C, Table S3D-S3F). The location of molecules coimmunoprecipitating with cld7 (the cld7-EpCAM complex) versus cld7mP differed only with respect to an abundance of cytoskeleton molecules preferentially associating with cld7mP (Fig. 3A). This difference was largely waved in TEX precipitates, both wt and cld7mP TEX precipitates containing cytoskeleton proteins. Instead, TEX-cld7mP abundantly precipitated with protein complexes, mostly of the proteasome, and junction proteins, whereas Tspan8, CD49c and ezrin were enriched in wt-TEX precipitates (Fig. 3B,3C).

**Figure 4:**
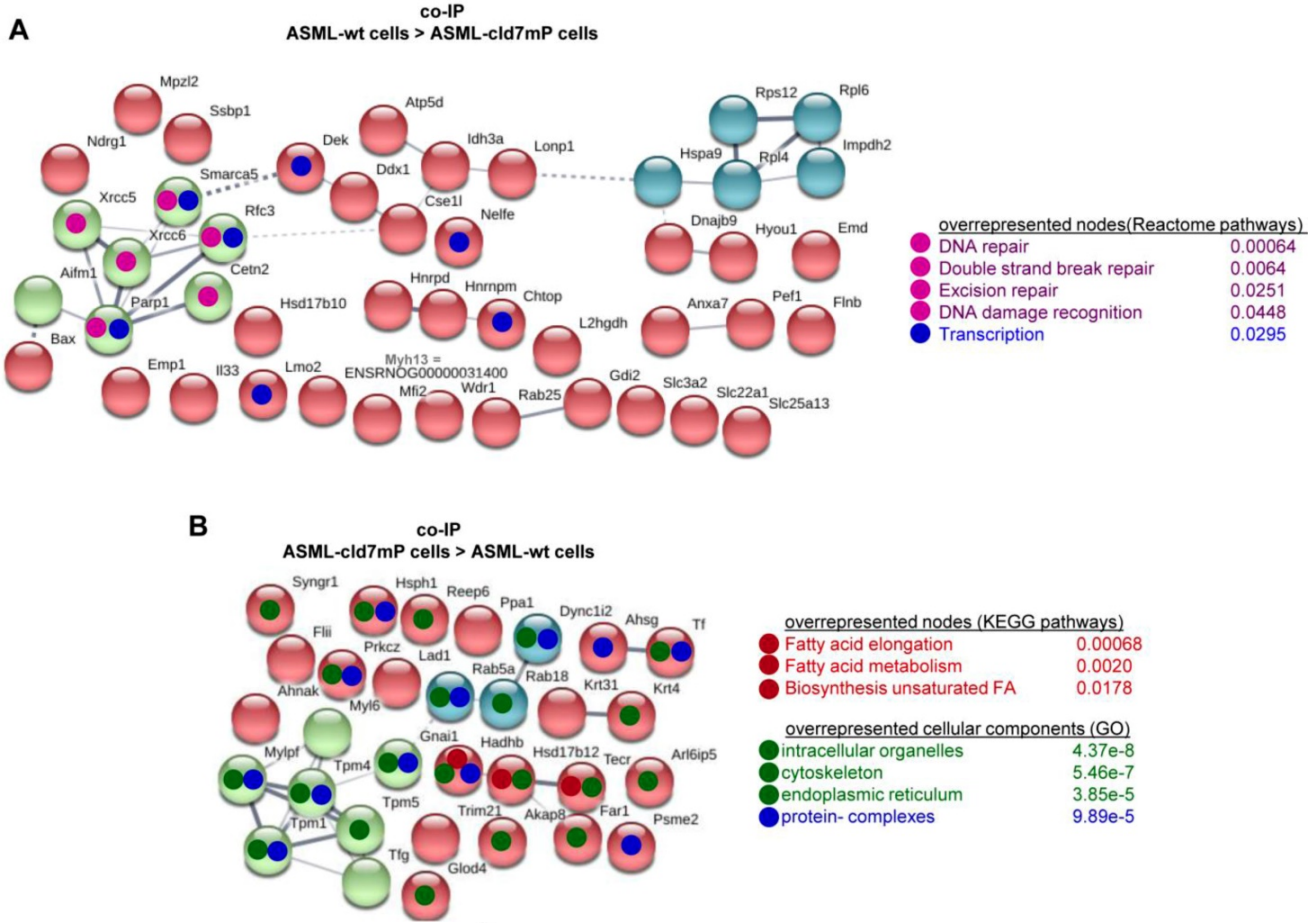

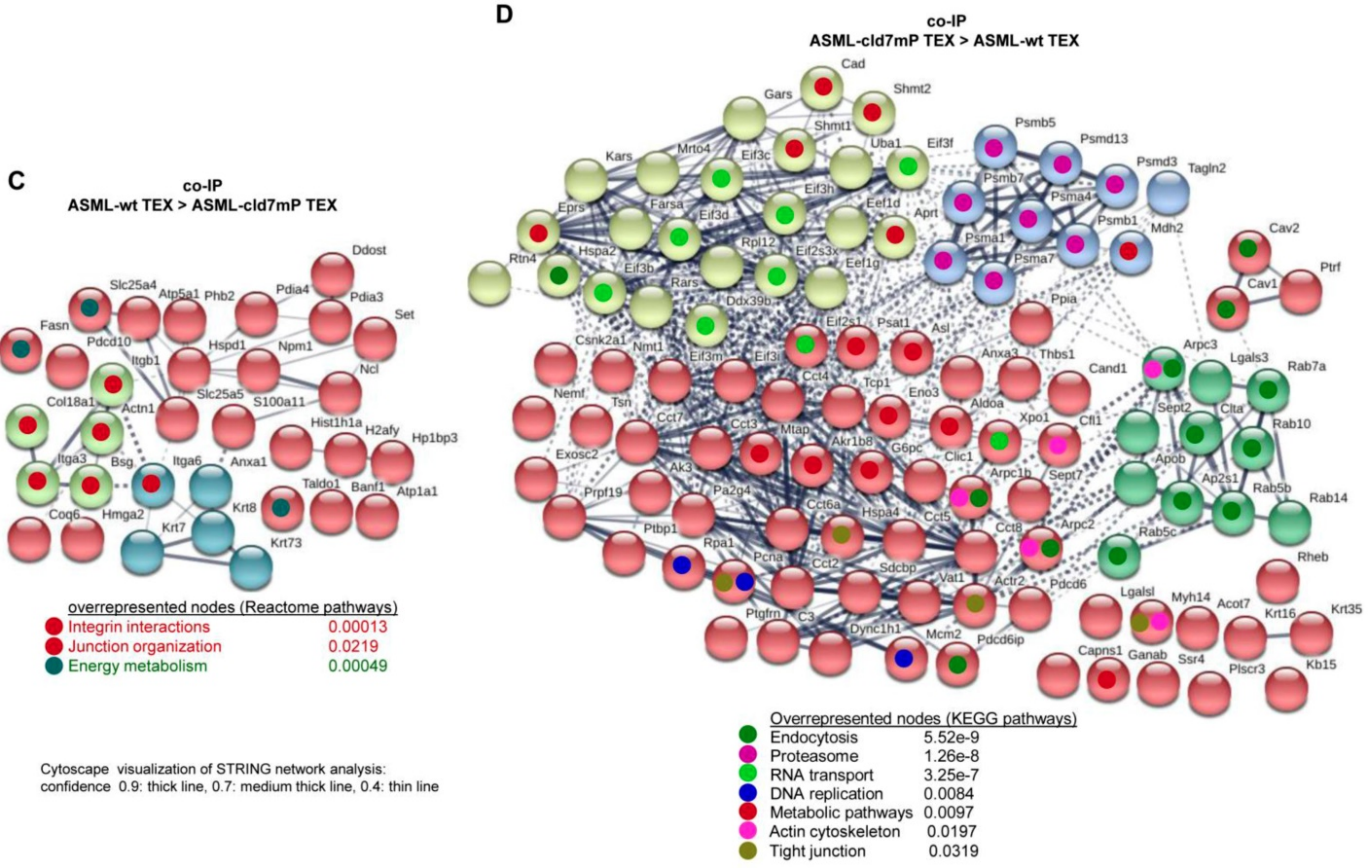
** Major activities of cld7-associated proteins in cells and TEX**. Lysates of ASML-wt and -cld7mP reconstituted cells and TEX were precipitated with anti-cld7. Precipitates were subjected to NanoLC-ESI-MS/MS proteome analysis. Distinctly recovered protein in coimmunoprecipitates of wt and cld7mP cells and TEX were subjected to STRING network analysis. Connectivity and major activities of overrepresented proteins in (A) wt or (B) cld7mP cells and (C) wt or (D) cld7mP TEX are shown. (A-D) The thickness of the connecting lines correspond to the confidence level; overrepresentation according to Reactome pathways, KEGG pathways or cellular components is indicated by a color-matching dot; full names of synonyms in Table S6. Connectivity and major activities strikingly differ in dependence on cld7 palmitoylation. This accounts particularly for TEX, where several connectivity clusters were only recovered in coimmunoprecipitates with non-palmitoylated cld7, suggesting a broader range of proteins being recruited into TEX by non-palmitoylated cld7.

Importantly, STRING network analysis uncovered striking differences in the function of proteins coimmunoprecipitating with wt versus cld7mP in cells and TEX. Proteins overrepresented in coimmunoprecipitates in ASML-wt cell lysates were mostly engaged in repair (Fig. 4A), while proteins overrepresented in coimmunoprecipitates of ASML-cld7mP cell lysates were engaged in fatty acid biosynthesis/metabolism. There was also an overrepresentation of organelles, the cytoskeleton, the ER and protein complexes (Fig. 4B). On the other hand, extracellular matrix (ECM) and metabolism organizing proteins were overrepresented in wt-TEX precipitates (Fig. 4C). Network analysis of cld7mP-TEX precipitates revealed 4 independent clusters containing proteins engaged in endocytosis, proteasome degradation, RNA transport and metabolic pathways. The latter cluster also contained proteins involved in DNA replication and TJ components. Many of these proteins are directly or indirectly involved in exosome biogenesis (Fig. 4D).

Thus, in cells the majority of cld7-coimmunoprecipitating molecules are associated with palmitoylated, GEM-located cld7, whereas in TEX significantly more proteins are associated with non-palmitoylated cld7. These proteins are frequently not plasma membrane-integrated, indicating TEX recruitment of proteins that associate with cytoplasmic, non-palmitoylated cld7. The exclusive recovery of TJ components in cld7mP cell and TEX precipitates and the strong enrichment of proteasome components in cld7mP coimmunoprecipitating proteins in TEX confirm the distinct palmitoylated versus non-palmitoylated cld7 recruitment and indicate a considerable contribution of recycling cld7 to the TEX armament.

Briefly, cld7 contributes to shaping TEX in ASML and SW948. Distinct to cells, where mostly GEM-located, palmitoylated cld7 accounts for differences in the protein profile, in TEX cytoplasmic, non-palmitoylated cld7 is decisive.

### Cld7 and miRNA recruitment into TEX

**Figure 5:**
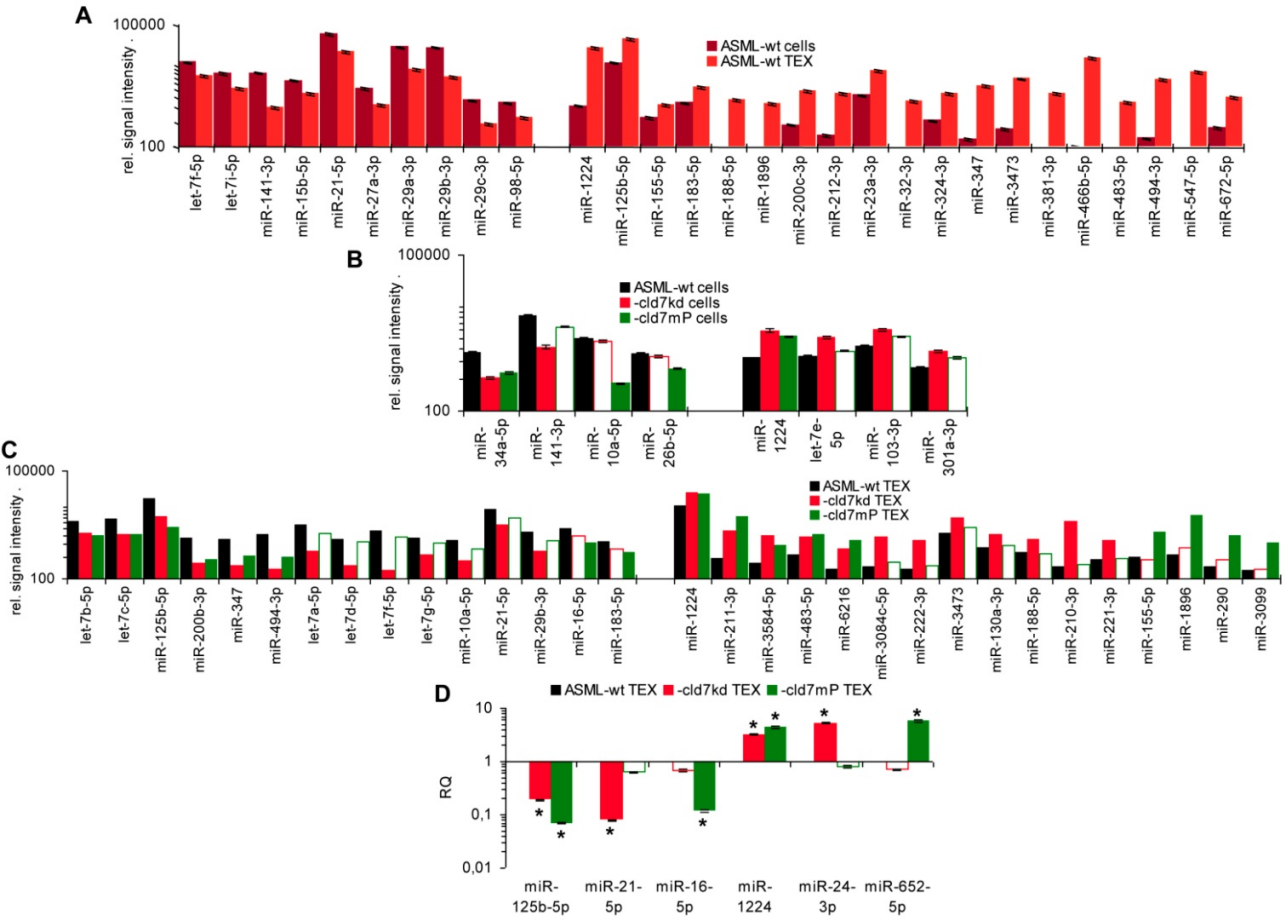
** Cld7-dependent miRNA recovery in TEX**. MiRNA recovery (Exicon version 7 microRNA) in ASML-wt, -cld7kd and cld7mP cells and TEX was evaluated by STAR aligner version 2.5.2a. Distinct recovery (≥2-fold difference) in (A) wt cells versus wt TEX, (B) wt versus cld7kd and cld7mP cells and (C) wt versus cld7kd and cld7mP TEX is shown; (D) Confirmation of distinct miRNA recovery in TEX by qRT-PCR, RQ values are shown, significant differences: *. Cld7 does not significantly affect miRNA transcription, processing or loading into ILV, from 801 miRNA in ASML-wt cells only 4 being distinctly down- or upregulated in cld7kd and/or cld7mP cells. Instead cld7 contributes to the travel of MVB, 16 miRNA, respectively, 18 miRNA from 766 miRNA in wt TEX being recovered at a higher, respectively, lower level in cld7kd and/or cld7mP TEX.

As the rules for recruiting miRNA into ILV differ from that of proteins, we finally analyzed, whether cld7 affects miRNA recruitment. Differences in the miRNA profile between wt cells and TEX confirmed selective miRNA recruitment into TEX 28, 19 miRNA being increased and 10 miRNA being reduced in ASML-wt TEX compared to cells (Fig.5A). Thus, we asked for a potential contribution of cld7. Two from 800 miRNA in ASML-wt cells were found at a reduced level in ASML-cld7kd cells and 3 in ASML-cld7mP cells; 4 miRNA were recovery at a higher level in ASML-cld7kd and 1 at a higher level in ASML-cld7mP than -wt cells (Table S4A, Fig.5B). Instead, 13 miRNA were discovered at a reduced level in ASML-cld7kd TEX and 8 in ASML-cld7mP-TEX; 12 miRNA were high in ASML-cld7kd and 9 in ASML-cld7mP than wt TEX (Table S4B, Fig.5C), confirmed in TEX for selected miRNA by qRT-PCR (Fig.5D). These findings indicated a possible, though minor contribution of cld7 recruiting miRNA into TEX.

**Figure 6:**
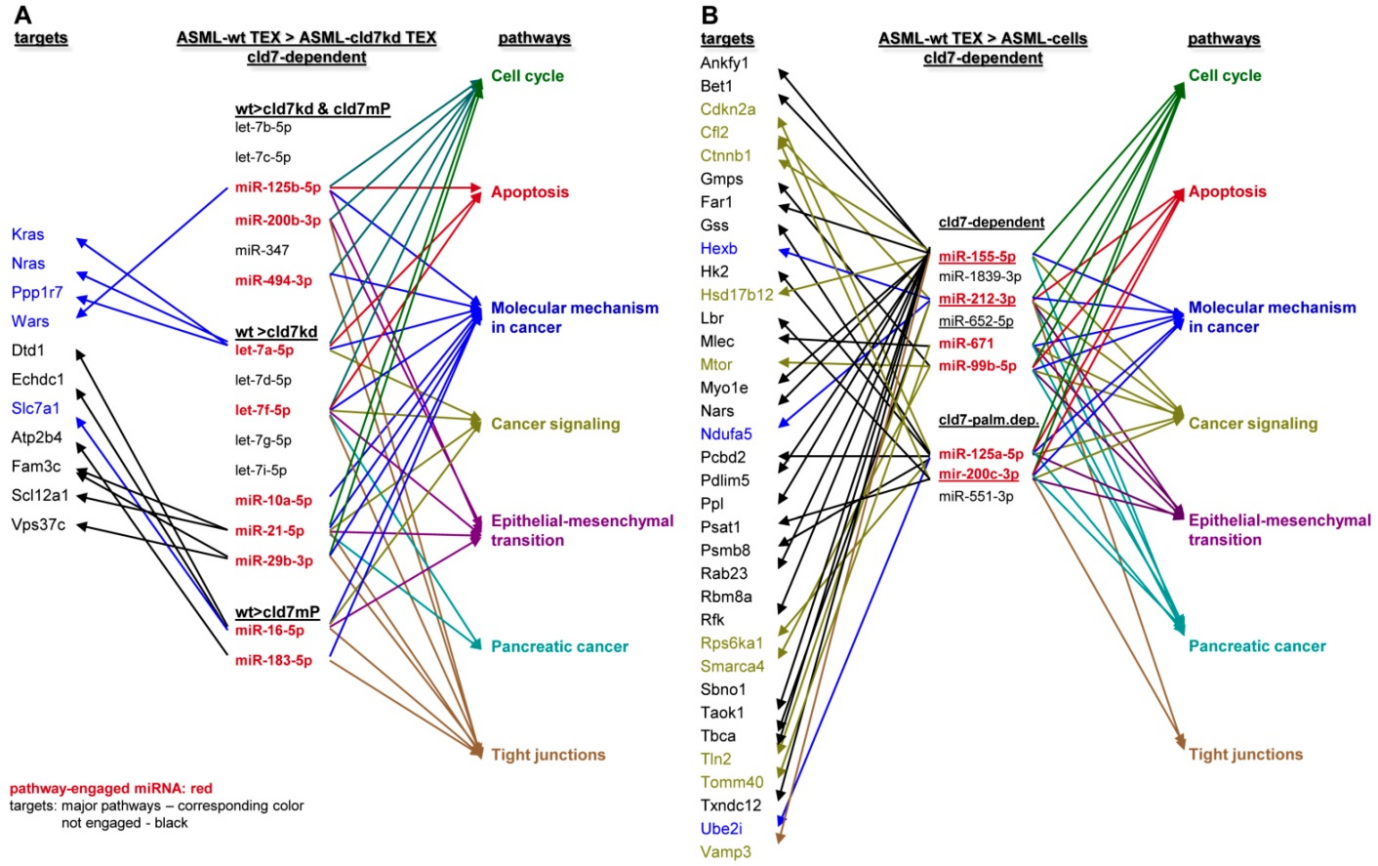
** Functional assignment of miRNA distinctly recovered in TEX in dependence on claudin7**. (A) Predicted targets of cld7-dependent distinctly recovered miRNA were searched for by http://www.microrna.org and http://www.targetscan.org; miRNA having predicted targets engaged in PaCa relevant functions, as revealed by STRING analysis, are indicated in red. In the following selection, PaCa function relevant miRNA higher in wt than cld7kd and/or cld7mP were correlated with reduced mRNA recovery in wt compared to cld7kd/cld7mP TEX. Only 5 mRNA being predicted targets for 3 miRNA are engaged in molecular mechanisms in cancer. (B) The same stepwise coordination as shown in (A) was used to define cld7-dependent miRNA overexpression in TEX versus cells. Six cld7/cld7mP dependent miRNA enriched in TEX compared to cells had predicted targets engaged in PaCa relevant pathways. All 6 miRNA had targets lower in wt TEX than cells depending on cld7/cldmP, which, however only were of relevance for molecular mechanisms in cancer and cancer signaling. (A,B) the same color code was used for the functional assignment of predicted and confirmed target. Confirmed targets that are not engaged in PaCa relevant function are shown in black, full names of synonyms are given in Table S6. Cld7-dependent miRNA recruited into TEX can be engaged in a wide range of cancer cell related activities. However, very few (TEX) or few (cells) predicted targets were recovered at a reduced level in dependence on cld7 or cld7mP expression, frequently being engaged in molecular mechanisms in cancer.

To obtain further hints, we proceeded searching for the recovery of predicted targets and their possible engagement in cancer progression, where the comparison between wt- and cld7kd- or cld7mP-TEX could be indicative for the origin of the miRNA. Predicted targets for 16 miRNA higher in wt than cld7kd and/or cld7mP TEX were searched by miRNA databases (http://www.microrna.org, http://www.targetscan.org). IPA-based STRING pathway analysis revealed that from these 16 miRNA 10 targeted mRNA engaged in cancer relevant pathways, mostly in molecular mechanism in cancer, cell cycle and TJ regulation. IPA was used defining, which of the predicted targets are discovered at a higher level in cld7kd- and cld7mP-TEX. Only for 6 miRNA high in wt-TEX, predicted targets were found at a higher level in cld7kd- and/or cld7mP-TEX. Only 5 from 11 predicted targets were relevant for cancer, being engaged in molecular mechanisms of cancer (Fig.6A). This excludes a major impact of cld7-dependent TEX miRNA on cld7-dependent target modulation within TEX. Following the same procedure, we search for the impact of cld7-dependent high miRNA in TEX on ASML-cld7kd or -cld7mP cells. Six of 9 miRNA had predicted targets engaged in cancer-related pathways. From the predicted targets, 35 were found at a higher level in ASML-cld7kd and/or -cld7mP cells, of which 13 are engaged in molecular mechanisms in cancer or cancer signaling (Fig.6B). The finding is in line with TEX miRNA possibly affecting target cell mRNA.

MiRNA recovery in SW948-wt and -cld7kd cells and TEX resembled recovery in ASML cells/TEX. From 17 miRNA higher in SW948-wt cells and 21 in TEX, 12 were higher in both wt cells and TEX. From 4 miRNA higher in cld7kd cells, 2/25 were also higher in cld7kd TEX (Table S4C, Fig.S4A,S4B). Notably, miRNA up- or downregulated in HT29-wt and HT29-cld7kd cells 9 and TEX were to >90% identical with those in SW948 cells and TEX (data not shown), indicating differing patterns of recovery not being a peculiarity of a specific line. From the 21 miRNA higher in wt than cld7kd TEX only 4 (let-7i-5p, miR-31-5p, -4763-3p, -8089) have 18 predicted targets that expression is higher in cld7kd than wt cells. However, all of these proteins are engaged in colorectal cancer-related activities, predominantly in cancer signaling (Fig.S4C). Controlling whether the miRNA higher in wt- than cld7kd-TEX target RNA/proteins downregulated in SW948-wt TEX, revealed that from 63 proteins higher in cld7kd than wt TEX only 15 were predicted targets of 9 miRNA higher in wt than cld7kd TEX. Except for 4 (BMP <Bone morphogenetic protein>7, MAP4K <Mitogen-activated protein kinase kinase>4, MET <Hepatocyte growth factor receptor>, Rho <Rho-related GTP-binding protein>) these predicted targets were not engaged in CoCa-related activities (Fig.S4D). Confirming the analysis of ASML-TEX, predicted targets of 4 miRNA high in wt TEX are engaged in CoCa-relevant activities and showed reduced expression in wt cells. On the other hand, only 4 predicted targets higher in cld7kd than wt TEX are potentially relevant for CoCa progression. This argues against miRNA that recruitment into TEX is cld7-dependent exerting a major impact on cld7kd tumor cells.

**Figure 7:**
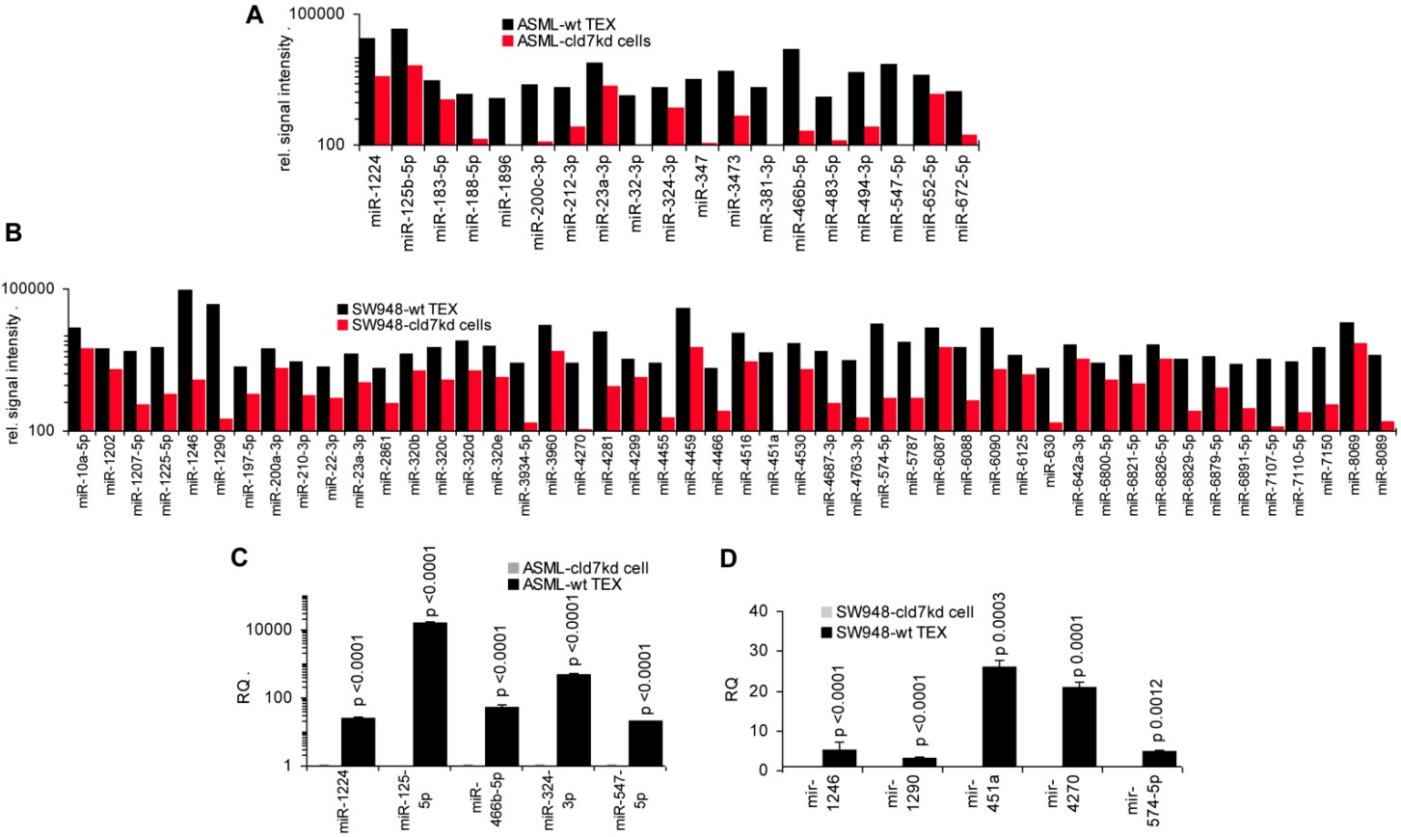

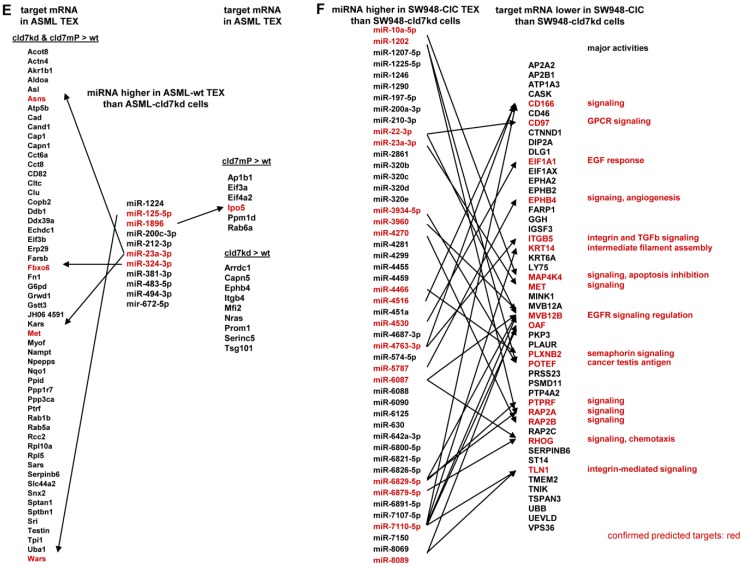
** The potential impact of TEX miRNA on cld7kd cells**. Recovery of miRNA was compared in ASML-wt and SW948 wt TEX with the recovery in cld7kd cells. (A,B) Significantly higher (>2-fold signal strength) miRNA recovery in ASML-wt and SW948-wt TEX than -cld7kd cells and (C,D) confirmation for selected miRNA by qRT-PCR; RQ values are shown and p values are indicated. (E) IPA analysis was used for correlating proteins recovered at a higher level in cld7kd and cld7mP TEX with predicted targets (http://www.microrna.org and http://www.targetscan.org) of miRNA enriched in ASML-wt TEX as described in Fig.6; (F) miRNA higher in SW948-CIC TEX than -cld7kd cells was correlated with lower recovery of predicted targets in SW948-CIC than -cld7kd cells, major activities (STRING analysis) of predicted targets are indicated. (full names of synonyms in Table S6). Independent of cld7 expression, there is a very poor correlation between miRNA and mRNA/protein in TEX (ASML), indicating that mRNA in TEX may only exceptionally be silenced by TEX miRNA. On the other hand, a considerable number of miRNA are enriched in wt TEX compared to cld7kd cells and could potentially affect the target cell mRNA profile. In addition, a high number of predicted mRNA/proteins are lower in SW948-wt than -cld7kd cells and thus could be targeted by miRNA high in SW948-wt TEX, the functional analysis suggesting an impact on cancer-promoting signaling pathways.

To support this hypothesis, we controlled, whether miRNA recovered at a higher level in wt-/CIC-TEX than cld7kd cells would affect predicted targets relevant for tumor progression. In ASML-wt TEX 19 miRNA and in SW948-wt TEX 48 miRNA were higher than in cld7kd cells, which was confirmed for selected miRNA by qRT-PCR (Fig.7A-7D).

Analyzing in the ASML model, whether higher miRNA in wt TEX than cld7kd cells is accompanied by low recovery of predicted targets in TEX revealed that this is not the case. Only 5 predicted targets (4 in cld7kd and cld7mP TEX, 1 in cld7mP TEX) of 4 of 11 miRNA higher in wt-TEX showed reduced expression in wt-TEX (Fig.7E), confirming TEX miRNA not affecting mRNA within TEX. High miRNA in TEX affecting before delivery the mRNA in the donor cell would imply reduce mRNA in wt cells with high miRNA recovery in wt TEX. This could not be excluded. From the 48 miRNA higher in SW948-CIC TEX than SW948-cld7kd cells, 17 had predicted targets with lower expression in CIC than SW948-cld7kd cells, the predicted targets playing central roles in signal transduction (Fig.7F).

Taken together, changes in the miRNA profile of cld7kd and cld7mPalm cells and TEX argue against cld7 playing a major role in miRNA processing or loading into ILV. Nonetheless, non-palmitoylated cld7 might assist the transport from MVB into TEX. Furthermore, high miRNA in TEX affecting target cell mRNA could, at least, not be excluded.

## Discussion

Cld7, a CoCIC and PaCIC biomarker 9,22, is TJ integrated 12 or associated with EpCAM and Tspan8 in internalization prone GEM 8. CIC predominantly act by message transfer via CIC-TEX into Non-CIC and host cells 30-32 and both, TJ- and GEM-derived cld7 are recovered in TEX 10. We here elaborated, whether palmitoylated and/or non-palmitoylated cld7 contributes to TEX assembly and whether this has an impact on Non-CIC. Proteome and miRNA analyses suggested major, but distinct contributions of palmitoylated and non-palmitoylated cld7 to protein recruitment. An active contribution to miRNA processing and ILV-loading is unlikely, but cld7 might assist the miRNA transfer from TEX into targets.

### Comments on the tumor models

We want to mention two technical aspects of the presented experiments.

GEM-derived, palmitoylated cld7 and possibly TJ-derived, non-palmitoylated cld7 are recovered in TEX 10,21,22. Approaching the question on the pathways of GEM- versus non-GEM-derived cld7 integration into TEX, ASML-cld7kd cells were transfected with cld7mP. However, due to technical difficulties collecting sufficient quantities of the two TEX populations, we did not separately collect TEX containing palmitoylated or non-palmitoylated cld7.

The majority of experiments with human cld7 were performed with SW948- and HT29-wt and -cld7kd cells 9 and TEX. The data being presented only for SW948 cells and TEX in the sake for clarity of presentation, it should be noted that results of the proteome and miRNA analyses were largely overlapping strengthening the general validity of cld7-promoted activities.

### GEM- and suggested TJ-associated cld7 are distinctly recruited into TEX and account for selective TEX compositions

Hints towards the engagement of palmitoylated versus non-palmitoylated cld7 to protein recruitment into TEX were obtained comparing wt versus cld7kd and cld7mP cells and TEX and by proteome analysis of proteins coimmunoprecipitating with cld7 in wt versus cld7mP cells and TEX.

Abundant recovery of heterogeneous ribonucleoproteins and histone components in TEX was repeatedly described 36-38. Recruitment of heterogeneous ribonucleoproteins, part of the spliceosome complex, may proceed during RNA processing 39-41. Integration of these proteins into TEX was, at least partly, cld7-independent. Some minor differences between recruitment in the absence of cld7 versus cld7mP could indicate that non-palmitoylated cld7 becomes enriched in TEX via vesicles delivered from the endoplasmic reticulum (ER) or the Golgi, an on/off shuttle of attached proteins between Golgi and ER being documented 42.

Another class of strongly exosome-enriched proteins is tetraspanins 43,44. Abundant recruitment into exosomes includes directly tetraspanin-associated transmembrane proteins and transmembrane proteins loosely associated via their location in GEM or via association with first rank tetraspanin partners as well as cytosolic proteins attached to the inner membrane of GEM by palmitoylation or myristoylation 45,46. Finally, cytoskeleton and cytoskeleton linker proteins, which bind to tetraspanin-associated molecules are also recruited during exosome biogenesis 47,48. There is no indication for cld7 being engaged in recruiting GEM components into TEX. In ASML and SW948 wt-TEX only radixin and tubulin subunits and in ASML wt-TEX additionally the α2 integrin chain and CD166 are found at a higher level than in cld7kd-TEX. Instead, proteins engaged in intracellular vesicle-mediated transport, in membrane trafficking and the transport between intracellular organelles are strikingly enriched in wt- compared to cld7kd-TEX. The distinct accumulation of transporters in wt-, cld7kd- and cld7mP-TEX points towards preferential recruitment via cld7mP, the assumption being supported by differences in proteins coimmunoprecipitating with cld7 versus cld7mP. First, coimmunoprecipitation with cytoskeletal proteins was especially seen in cld7mP cell lysates and a high number of complex-associated, mostly proteasome-incorporated proteins precipitated with cld7mP in TEX. Second, the difference between cld7 and cld7mP became even more striking, when searching for connectivity of the coimmunoprecipitating molecules. In wt compared to cld7mP cells repair mechanisms were overrepresented, while in cld7mP cells fatty acid synthesis/metabolism and intracellular proteins and organelles were enriched. A linkage between exosomes as lipid transporters and an impact on cancer progression was repeatedly described 49-51. It is also known that cld, including cld7 are engaged in lipid transport 22,52, and that this feature is transferred into exosomes 10. In wt- compared to cld7mP-TEX coimmunoprecipitates unraveled a preferential linkage in wt TEX to integrins and interacting proteins, arguing for a reduction of membrane-associated proteins in cld7mP-TEX. The high number of proteins coimmunoprecipitating preferentially with cld7mP in TEX are more difficult to correlate with selective functions. Beside proteasome and TJ components, endocytosis-engaged molecules preferentially coimmunoprecipitated with cld7mP. TJ-integrated cld internalization requires phosphorylation and is suggested to follow a specific endocytosis pathway 53,54. First to note, enrichment of cld was only seen in coimmunoprecipitation of cld7mP TEX, pointing towards cld7mP being at least partially derived from TJ. In line with this, TJ cld1 contributes to hepatitis C virus uptake and is recovered in intracellular vesicles, the transport of the internalized cld1-hepatitis C virus transfer requiring CD81 55, which was also recovered in coimmunoprecipitates with cld7mP. Furthermore, proteins engaged in endocytosis including transporters, proteasome components and metabolic pathway components are overrepresented. Taken together, these findings strengthen our assumption that during the intracellular passage of TJ-derived cld7 distinct partners become engaged in driving cld7 into TEX. Though further experiments are required to elucidate the pathway of TJ-derived cld7 in TEX, there is compelling evidence for non-palmitoylated cld7 furnishing TEX with components that might particularly contribute to uptaken TEX being transferred to and digested in the proteasome as well as to transcription activation and RNA translation.

Finally, it should be mentioned that the recruitment of signaling molecules into TEX largely was independent of cld7 or cld7mP. Prominent components are integrins, tetraspanins, RTK, Ephrins/Ephrin receptors and cytosolic signaling molecules like scr, fyn, lyn and a wide range of GTPases, all repeatedly described contributing to the crosstalk between TEX and the host, including the ECM, stromal cells and endothelial cells as well as to increase tumorigenicity, epithelial-mesenchymal transition and metastatic aggressiveness 10,35,46,50,51,56-61.

Taking into account the monoclonality of ASML cells 62, our findings sustain the diversity of Exo derived from a single cell 10,63 and strengthen independent recruitment of GEM- and likely TJ-derived cld7 into TEX. Obviously, endosome trafficking proceeds along distinct routes and is guided by different transporters/transporter complexes. The protein profile of palmitoylated cld7 containing TEX largely overlaps with that of GEM-derived TEX. The association of tetraspanins with integrins and the location in GEM, which harbor signaling molecules, the slight dominance of kinases in palmitoylated versus cld7mP-containing TEX could well expand the range of communications, the enrichment of RTK as well as membrane attached and cytosolic signaling molecules being suggested playing a decisive role in CIC-TEX modulating host tissue and Non-CIC 58. Non-palmitoylated, likely TJ-derived cld7 actively contributes to the recruitment of cld, proteasome subunits, vesicle transporters, anion and lipid transporters and RNA translation 64-66. Thus, the suggested TJ-derived cld7-dependent TEX populations expand the range of activities, whereby CIC-TEX could affect host and non-metastatic tumor cells. Finally, we suggest lipid analysis of TEX containing non-palmitoylated cld7 to provide further hints for their origin from TJ, which are enriched in sphingomyelin with long-chain fatty acids and cholesterol, depletion of cholesterol abolishing the formation of TJ 67,68.

### Cld7 and miRNA recruitment into TEX

Most amply demonstrated for proteins and miRNA 37,69, all components of Exo are function competent 31. Thus, we wondered whether cld7 might also contribute to miRNA recruitment.

Very few miRNA were distinctly recovered in ASML and SW948 cells depending on the presence of (palmitoylated) cld7. As elaborated for ASML TEX, recruitment of miR-200c-3p was palmitoylated cld7-dependent, recruitment of an additional 3 miRNA (miR-155-5p, miR-121-3p, miR-652-5p) was cld7-dependent irrespective of palmitoylation. In concern about a contribution of cld7 to miRNA recruitment into TEX, a significant number of miRNA is distinctly recovered in wt- versus cld7kd- or cld7mP-TEX without corresponding changes in cells. We interpret these findings that cld7/cld7mP contributes to MVB transport, but is not decisive for ILV-loading. Steps/molecular complexes of MVB transport that are supported by palmitoylated or non-palmitoylated cld7 require further elaboration.

Irrespective of miRNA recruitment, there was no evidence that TEX miRNA affected TEX mRNA/protein. This accounted for predicted target mRNA/protein of miRNA high in TEX. In addition, from 70 proteins higher in cld7kd- and/or cld7mP- than wt-TEX, only 5 were predicted targets of 4 miRNA lower in cld7kd- than wt-TEX. Instead, from 49 proteins with lower expression in wt- than cld7kd-cells, 17 are predicted targets of miRNA higher in wt-TEX than cld7kd-cells, the predicted targets mostly being engaged in central signaling activities. The finding is in line with the suggested impact of TEX miRNA on target cell mRNA 70,71.

In brief, mostly non-palmitoylated cld7 contributes to the transport of miRNA-loaded MVB into TEX. The target proteins of these miRNA are not reduced in TEX, indicating that miRNA recruitment was independent of mRNA/protein recruitment and that TEX miRNA does not affect mRNA within TEX. Instead, predicted mRNA targets of abundant TEX miRNA were reduced in wt- compared to cld7kd-cells, which suggests TEX miRNA supporting “tumor competence” of cld7kd-cells 72. However, functional in vitro and in vivo studies on the impact of TEX-cld7 on cld7kd targets revealed a minor contribution of TEX miRNA compared to TEX proteins 73.

## Material and methods

Tumor lines: The human CoCa lines SW948 and SW948-cld7kd 9,74 and the rat PaCa line ASML, ASML-cld7kd and ASML-cld7mP 22,62 are maintained in RPMI1640/10%FCS/glutamine/ antibiotics, medium containing 0.5mg/ml G418 for maintenance of the kd lines and additionally 120µg/ml hygromycin for ASML-cld7mP 22. SW948 and SW948-cld7kd cells were recently authenticated by STR loci for full matching with the parental cell line (DSMZ, Braunschweig, Germany). Cells were regularly checked for mycoplasma contamination by a fluorescence detection Kit (Thermo Fisher, Germany).

Antibodies are listed in Table S5A.

CIC enrichment and TEX preparation: SW948-CIC are enriched by spheroid growth 9. After 3 rounds of spheroid growth, cells are harvested for TEX collection. The monoclonal ASML line displaying CIC features 62, wt cell-derived TEX were considered as CIC-TEX. Culture supernatants (2x48h in serum-free medium) were cleared (2x10min, 500g, 1x20min, 2000g, 1x30min, 10000g) and filtered (0.22µm). Filtered supernatants were centrifuged (120min, 100000g), the pellet was washed (PBS, 120min, 100000g), resuspended in 40% sucrose, overlaid by a 30%-5% discontinuous sucrose gradient and centrifuged (16h, 100000g), collecting 12 fractions of 320µl. TEX are enriched in light density fractions d1.15-1.56g/ml (fractions 1-4) [75, Suppl.MM]. Protein concentrations were determined by Bradford.

Immunoprecipitation (IP) and WB: Cells and TEX were lysed in HEPES buffer/1%Lubrol/1mM PMSF/1mM NaVO_4_/10mM NaF/protease inhibitor mix (30min, 4°C), mild lysis conditions were used for avoiding the destruction of loosely attached protein complexes. Lysates (cells 30µg, TEX: 10µg) were centrifuged (13000g, 10min, 4°C), mixed with antibody (1h, 4°C) and incubated with ProteinG-Sepharose (1h). Washed complexes were dissolved in Laemmli buffer. For WB, lysed cells and TEX were dissolved in Laemmli buffer and resolved on 10%-12% SDS-PAGE. After protein transfer, blocking, blotting with antibodies, blots were developed with enhanced chemiluminescence reagent.

Protein elution, tryptic digestion, mass spectrometry and database searches: Cell and TEX lysates and dissolved immunoprecipitates were separated by 1D SDS gel electrophoresis. After staining with Coomassie lanes were cut into ten slices. After tryptic digestion, peptides were analyzed by nanoLC-ESI-MS/MS and subjected to quantification [76, Suppl.MM].

miRNA: TEX were pretreated with RNase. Following the supplier's suggestion (Qiagen, Hildesheim, Germany), the miRNeasy Minikit was used to extract cell and TEX miRNA.

Microarray miRNA analysis: miRNA analysis of cells and TEX was performed at the Core facility of EMBL, Heidelberg, using the Agilent microRNA microarray evaluating quadruplicates of three independent preparations. Data are deposited at GEO (human miRNA: http://www.ncbi.nlm.nih.gov/geo/query/acc.cgi? acc=GSE119031, GSE119032, -GSE 11903, rat miRNA: http://www.ncbi.nlm.nih.gov/geo/query/acc.cgi? acc=GSE120185). Mean values of normalized data (Agilent Feature Extraction Software, STAR aligner version 2.5.2a) were compared. Differential recovery was defined by ≥2-fold changes in signal strength.

Proteome and miRNA analysis: PANTHER (http://pantherdb.org), KEGG (http://www.kegg.jp), Reactome (https://reactome.org), andSTRING (http://string-db.org) databases were used for pathway analysis. IPA was used for miRNA analysis and for correlating miRNA with protein expression after predicted targets were selected by miRNA databases (http://www.microrna.org, http://www.targetscan.org).

Real-time PCR (qRT-PCR): Real-time polymerase chain reaction (PCR) was performed using a standard TaqMan PCR kit protocol on an Applied Biosystems 7900HT Sequence Detection System (Applied Biosystems). Primers are listed in Table S5B**.** Small nuclear snRNA U6 was used as internal control [77, Suppl.MM].

Flow-cytometry of cells followed routine procedures. TEX (10-15µg) were incubated with 1µl aldehyde-sulfate latex beads (LB) (4µm) (Invitrogen) in PBS/1% BSA (90min, 20°C, shaking). After centrifugation, free binding sites were blocked (100mM glycine in PBS, 1h). TEX-coated LB were washed (PBS/1% BSA). Staining of 1µl TEX-coated LB followed the protocol for cell staining. For intracellular staining, cells/TEX were fixed and permeabilized. Samples were analyzed in a FACSCalibur using the CellQuest program.

Statistics: IBM SPSS software (IBM, New York, NY, USA) was used for statistical evaluation. In vitro assays were run in triplicates and repeated at least three times. P-values are derived from two-tailed Student's *t* test, analysis of variance. If not indicated otherwise, p-values <0.05 were considered significant.

## Conclusion

The CIC marker cld7 is abundantly recovered in PaCIC- and CoCIC-TEX, which transfer CIC messages into host cells and non-CIC. As TEX-cld7 can be derived from GEM or TJ, defining the contribution to TEX biogenesis became important. Palmitoylated cld7 assists the recruitment of GEM-associated proteins including membrane-attached cytoskeletal components and signaling molecules. Non-palmitoylated, likely TJ-derived cld7 most prominently recruits transporters and assists miRNA-loaded vesicle transport. Thus, TEM- and TJ-derived cld7 distinctly, but additively affect TEX composition. Awareness of distinct TEX populations delivered by a single cell only recently received attention, but essentially needs to be taken into account optimizing therapeutic strategies. The information on cld7-dependent TEX assembly provides a solid ground elaborating at the molecular level optimized strategies to interfere with TEX-cld7 metastasis-promoting activities.

## Supplementary Material

Supplementary figures and tables.

## Abbreviations

CIC: cancer-initiating cell
cld7: claudin7
CoCa: colorectal cancer
ECM: extracellular matrix
EE: early endosomes
Exo: exosomes
GEM: glycolipid-enriched membrane domain
ILV: intraluminal vesicles
IP: immunoprecipitation
kd: knockdown
ko: knockout
LB: latex beads
mP: mutated palmitoylation site
MVB: multivesicular body
PaCa: pancreatic cancer
TEX: tumor-derived microvesicles
TJ: tight junction
WB: Western blot
wt: wild-type. Full names of synonyms are shown in alphabetic order in Table S6
