# Supplementary material for: Distinct Origin of Claudin7 in Early Tumor Endosomes Affects Exosome Assembly
Source: Int J Biol Sci. 2019 Aug 19;15(10):2224–39. doi: 10.7150/ijbs.35347 (PMC6775303; doi:10.7150/ijbs.35347)
Supplement: Supplementary file 1 — Supplementary figures and tables. [file ijbsv15p2224s1.pdf]

Supplementary data

Distinct origin of Claudin7 in early tumor endosomes affects exosome assembly

Daisuke Kyuno[a,b], Nathalie Bauer[a], Martina Schnölzer[c], Jan Provaznik[d], Eduard Ryschich[a], Thilo Hackert[a], Margot Zöller[a]

[a] Department of General, Visceral and Transplantation Surgery, University of Heidelberg, Germany, [b] Department of Surgery, Surgical Oncology and Science, Sapporo Medical University, Sapporo, Japan, [c] Functional Proteome Analysis, DKFZ, Heidelberg, Germany, [d] Gene Core Center, EMBL, Heidelberg, Germany

Supplementary Tables

Table S1 Distinct recovery of proteins in ASML-wt versus -claudin7kd / -claudin7mP cells and TEX
Table S2 Distinct recovery of proteins in SW948-wt versus -claudin7kd cells and TEX
Table S3 Claudin7 protein complexes in cells and TEX
Table S4 Claudin7-dependent miRNA recovery in cells and TEX
Table S5 Antibodies and Primers
Table S6 Full names of synonyms (alphabetic)

Supplementary Figures

Figure S1 Functional activities of proteins distinctly recovered in ASML-wt, -cld7kd and -cld7mP cells and TEX
Figure S2 The impact of cld7 on the protein profile of colorectal cancer cells and TEX and functional activities of proteins distinctly recovered in cells and TEX.
Figure S3 Overview on molecular functions of proteins coimmunoprecipitating with claudin7 in ASML-wt and -cld7mP cells and TEX
Figure S4 Cld7-dependent recruitment of miRNA into colorectal cancer TEX

Supplementary Methods

Exosome preparation
Mass spectrometry and database searches
Real time PCR

Table S1

Distinct recovery of proteins in ASML-wt versus -cld7kd / -cld7mPalm cells and TEX

Table S1A

Proteins preferentially recovered in ASML-wt cells

	ASML-wt		-cldkd		-cld7mPalm		
Synonym	Prot.M	Pept.M	Prot.M	Pept.M	Prot.M	Pept.M	Protein Description
Stmn1	95	32	4	3	10	5	Stathmin
Mob1a	91	26			1	1	MOB kinase activator 1A
Atp1b1	71	18	4	2			Sodium/potassium-transporting ATPase ß1
ASCT2	134	17	4	2	2	1	Alanine/serine/cysteine/threonine transporter 2
Fancd2	125	17			1	1	Fanconi anemia group D2 protein homolog
Immt	38	17					MICOS complex subunit Mic60
Tubb3	149	17					Tubulin beta-3 chain
Smc1a	27	16			4	2	Structural maintenance of chromosomes 1A
Ppl	20	14	1	1			Periplakin
Itga6	19	12	4	2	5	3	Integrin, alpha 6
Itgb1	19	12	2	2	7	4	Integrin beta 1
Lrp1	19	12	7	3	8	4	LDL receptor related protein 1
Trip12	19	10	2	1	3	2	thyroid hormone receptor interactor 12
Acsl4	13	9	4	2			acyl-CoA synthetase long chain family memb. 4
Far1	49	9	3	2	3	2	Fatty acyl-CoA reductase 1
Ppp1cc	22	9					Serine/threonine-protein phosphatase
Rab31	23	9					Ras-related protein Rab-31
LOC102554637	18	8	4	1	2	1	anionic trypsin-2-like
LOC103694878	23	8	3	2			glutathione S-transferase theta-2-like
Pecr	20	8					Peroxisomal trans-2-enoyl-CoA reductase
Cbr4	13	7					Carbonic reductase 4
Dazap1	22	7					DAZ associated protein 1
Rps6ka3	9	6					Ribosomal protein S6 kinase
Hdac1	8	6					Histone deacetylase 1
Krt20	24	6					Keratin, type I cytoskeletal 20
Pola1	9	6					DNA polymerase alpha catalytic subunit
Hcfc1	8	5					host cell factor C1
Ogt	9	5					O-linked N-acetylglucosamine transferase
LOC100359539	8	5					ribonucleotide reductase M2 polypeptide
LOC100912445	7	5					leucyl-cystinyl aminopeptidase-like
Mlec	9	5	1	1			Malectin
Sarnp	7	5			1	1	SAP domain-containing ribonucleoprotein
Ccs	9	5					Copper chaperone for superoxide dismutase
Eno2	43	5					Gamma-enolase
Sfn	13	5					stratifin
Usp15	12	5					Ubiquitin carboxyl-terminal hydrolase
Abcb7	7	4	2	1			ATP-binding cassette sub-family B member 7
Akr1c12l1	6	4					Aldo-keto reductase family 1, C12-like 1
Arfip1	6	4			1	1	ADP-ribosylation factor interacting protein 1
Arid1a	4	4					AT-rich interaction domain 1A
Ccdc132	5	4	1	1			Syndetin
Cnpy3	6	4					canopy FGF signaling regulator 3
Gnai3	5	4					Guanine nucleotide binding protein α inhib. 3
Hibadh	6	4					3-hydroxyisobutyrate dehydrogenase
Ldhb	33	4					L-lactate dehydrogenase B chain
Gls	7	4					Glutaminase kidney isoform
Ltn1	5	4	2	1			listerin E3 ubiquitin protein ligase 1
Smarcc1	6	4					matrix assoc., actin dep. regulator chromatin c1

Table S1B

Proteins preferentially recovered in ASML-wt and ASML-cld7mPalm cells

	ASML-wt		-cldkd		-cld7mPalm		
Synonym	Prot.M	Pept.M	Prot.M	Pept.M	Prot.M	Pept.M	Protein Description
Eef1a1	395	20			431	17	Elongation factor 1-alpha 1
Rpl7	15	9			9	5	60S ribosomal protein L7
Rap1a	22	8			19	6	Ras-related protein Rap-1A
Rab6a	24	7			13	4	Ras-related protein Rab-6A
Gdi1	8	5			7	4	Rab GDP dissociation inhibitor alpha
Itgb4	8	5			5	4	Integrin beta 4
Sec23a	9	5			7	4	Sec23 homolog A, coat complex II component
Anlnl1	7	4			4	3	anillin, actin binding protein-like 1
Prpsap2	5	4	2	1	3	3	Pyrophosphate synthase-assoc. protein2
Rab35	31	4			19	3	Ras-related protein Rab-35
RGD1564698	11	4			8	3	similar to ribosomal protein S10
Serpinb6	5	4	2	1	12	9	serpin family B member 6

Table S1C

Proteins preferentially recovered in ASML-wt and ASML-cld7kd cells

	ASML-wt		-cldkd		-cld7mPalm		
Synonym	Prot.M	Pept.M	Prot.M	Pept.M	Prot.M	Pept.M	Protein Description
Hdlbp	32	17	21	11	10	4	High density lipoprotein bind. protein (Vigilin)
Eif4a2	50	15	44	10			Eukaryotic initiation factor 4A-II
Ppidl1	25	12	23	11			peptidylprolyl isomerase D-like 1
Eif2s3	18	10	14	9			Eukaryotic translate. initiation factor2 subunit 3
Arf3	38	10	61	13			ADP-ribosylation factor 3
Cdk1	19	10	24	11			Cyclin-dependent kinase 1
Lmna	14	10	15	10	6	3	Lamin A
Rps2	19	10	13	7			40S ribosomal protein S2
Rps15a	18	6	32	7			40S ribosomal protein S15a
Sec24a	7	6	15	7			SEC24 homolog A, COPII coat complex comp.
Ssb	9	6	6	4			Lupus La protein homolog
Cotl1	9	5	5	5			Coactosin-like protein
Abce1	5	5	3	2			ATP-binding cassette, sub-family E member 1
Ddx5	9	5	7	5			DEAD (Asp-Glu-Ala-Asp) box polypeptide 5
Mcu	9	5	6	4	2	1	mitochondrial calcium uniporter
Rpl10	13	5	15	5			60S ribosomal protein L10
Tubb1	29	5	33	4			tubulin beta 1 class VI
Rpl13a	8	4	8	5	2	1	60S ribosomal protein L13a
Tpm4	7	4	7	5			Tropomyosin alpha-4 chain

Table S1D

Proteins enriched in ASML-wt compared to -cld7kd and -cld7mP cells

Cargo concentration in the ER (R-RNO-5694530)

Sec24b	Protein Sec24b
Ctsz	Cathepsin Z
Sec24c	LOC685144 protein;Sec24c
Mia3	Protein Mia3

ER to Golgi Anterograde Transport (R-RNO-199977)

Q4AEF8	Coatomer subunit gamma-1;Copg1
Golgb1	Protein Golgb1
Sec24b	Protein Sec24b
Ctsz	Cathepsin Z
Sec24c	LOC685144 protein;Sec24c
Mia3	Protein Mia3
Napa	Alpha-soluble NSF attachment protein;Napa
Cope	Coatomer protein complex, subunit epsilon

Intra-Golgi and retrograde Golgi-to-ER traffic (R-RNO-6811442)

Golga4	Golgin subfamily A member 4
Q4AEF8	Coatomer subunit gamma-1;Copg1
Rab6a	Ras-related protein Rab-6A
Vamp4	Protein Vamp4
Kif4a	Protein Kif4a
Trip11	Protein Trip11
Napa	Alpha-soluble NSF attachment protein;Napa
Cope	Coatomer protein complex, subunit epsilon

Transport to the Golgi and subsequent modification (R-RNO-948021)

Q4AEF8	Coatomer subunit gamma-1;Copg1
Golgb1	Protein Golgb1
Sec24b	Protein Sec24b
Ctsz	Cathepsin Z
Sec24c	LOC685144 protein;Sec24c
Mia3	Protein Mia3
Napa	Alpha-soluble NSF attachment protein;Napa
Cope	Coatomer protein complex, subunit epsilon;Cope

Membrane Trafficking (R-RNO-199991)

Golga4	Golgin subfamily A member 4;
Q4AEF8	Coatomer subunit gamma-1;Copg1
Rab6a	Ras-related protein Rab-6A
Vamp4	Protein Vamp4
Kif4a	Protein Kif4a
Golgb1	Protein Golgb1
Ap2a1	Adaptor protein complex AP-2, alpha 1 subunit;Ap2a1
Sec24b	Protein Sec24b
Ctsz	Cathepsin Z;Ctsz
Sec24c	LOC685144 protein;Sec24c
Trip11	Protein Trip11
Rala	Ras-related protein Ral-A;Rala
Mia3	Protein Mia3
Napa	Alpha-soluble NSF attachment protein;Napa
Tf	Serotransferrin;Tf
Cope	Coatomer protein complex, subunit epsilon;Cope

Vesicle-mediated transport (R-RNO-5653656)

Golga4	Golgin subfamily A member 4;Golga4
Q4AEF8	Coatomer subunit gamma-1;Copg1
Rab6a	Ras-related protein Rab-6A
Vamp4	Protein Vamp4
Kif4a	Protein Kif4a
Golgb1	Protein Golgb1
Ap2a1	Adaptor protein complex AP-2, alpha 1 subunit;Ap2a1
Sec24b	Protein Sec24b
Ctsz	Cathepsin Z;Ctsz
Sec24c	LOC685144 protein;Sec24c
Trip11	Protein Trip11
Rala	Ras-related protein Ral-A;Rala
Lrp1	Protein Lrp1
Mia3	Protein Mia3
Napa	Alpha-soluble NSF attachment protein;Napa

Tf	Serotransferrin;Tf
Cope	Coatomer protein complex, subunit epsilon;Cope
**Asparagine N-linked glycosylation** (R-RNO-446203)	
Q4AEF8	Coatomer subunit gamma-1;Copg1
Golgb1	Protein Golgb1
Sec24b	Protein Sec24b
Ctsz	Cathepsin Z;Ctsz
Sec24c	LOC685144 protein;Sec24c
Mlec	Malectin;Mlec
Mia3	Protein Mia3
Napa	Alpha-soluble NSF attachment protein;Napa
Cope	Coatomer protein complex, subunit epsilon;Cope
**Post-translational protein modification** (R-RNO-597592)	
Q4AEF8	Coatomer subunit gamma-1;Copg1
rCG_63409	Proteasome subunit beta type;rCG_63409
Golgb1	Protein Golgb1
Sec24b	Protein Sec24b
Ctsz	Cathepsin Z;Ctsz
Sec24c	LOC685144 protein;Sec24c
Usp9x	Protein Usp9x
Smc1a	Structural maintenance of chromosomes protein 1A;Smc1a
Psmb5	Proteasome subunit beta type-5;Psmb5
Mlec	Malectin;Mlec
Ogt	UDP-N-acetylglucosamine--peptide N-acetylglucosaminyltransferase 110 kDa subunit;Ogt
Hcfc1	Protein Hcfc1
Usp15	Ubiquitin carboxyl-terminal hydrolase 15;Usp15;ortholog
Mia3	Protein Mia3;Mia3;ortholog
Napa	Alpha-soluble NSF attachment protein;Napa
Tp53bp1	Protein Tp53bp1
Cope	Coatomer protein complex, subunit epsilon;Cope
**Metabolism of proteins** (R-RNO-392499)	
Q4AEF8	Coatomer subunit gamma-1;Copg1
rCG_63409	Proteasome subunit beta type;rCG_63409
Golgb1	Protein Golgb1
Sec24b	Protein Sec24b
Ctsz	Cathepsin Z;Ctsz
Sec24c	LOC685144 protein;Sec24c
Usp9x	Protein Usp9x
Gnai3	Guanine nucleotide-binding protein G(k) subunit alpha;Gnai3
Smc1a	Structural maintenance of chromosomes protein 1A;Smc1a
Psmb5	Proteasome subunit beta type-5;Psmb5
Mlec	Malectin;Mlec
Ogt	UDP-N-acetylglucosamine--peptide N-acetylglucosaminyltransferase 110 kDa subunit;Ogt
Hcfc1	Protein Hcfc1
Usp15	Ubiquitin carboxyl-terminal hydrolase 15;Usp15
Eif2s3	Eukaryotic translation initiation factor 2 subunit 3, X-linked;Eif2s3
Mia3	Protein Mia3
Napa	Alpha-soluble NSF attachment protein;Napa
Tp53bp1	Protein Tp53bp1;Tp53bp1;ortholog
Cope	Coatomer protein complex, subunit epsilon;Cope
**Metabolism** (R-RNO-1430728)	
Gls	Glutaminase kidney isoform, mitochondrial;Gls
Ckb	Choline/ethanolamine kinase;Chkb
Akr1c12l1	Aldo-keto reductase family 1, member C12-like 1;Akr1c12l1
Acox1	Peroxisomal acyl-coenzyme A oxidase 1;Acox1
LOC102554637	Protein LOC102554637
rCG_63409	Proteasome subunit beta type;rCG_63409
Gls	Glutaminase liver isoform, mitochondrial;Gls2
Ap2a1	Adaptor protein complex AP-2, alpha 1 subunit;Ap2a1
Ckb	Creatine kinase B-type;Ckb
Hk2	Hexokinase-2;Hk2
Cbr4	Carbonyl reductase family member 4;Cbr4
A0A0G2K099	Uncharacterized protein;unassigned
Pdhb	Pyruvate dehydrogenase E1 component subunit beta, mitochondrial;Pdhb
Esyt1	Extended synaptotagmin-1;Esyt1

Table S3D continued

Ldhb	L-lactate dehydrogenase B chain;Ldhb
Psmb5	Proteasome subunit beta type-5;Psmb5
Idh3g	Isocitrate dehydrogenase [NAD] subunit gamma 1, mitochondrial;Idh3g
Pecr	Peroxisomal trans-2-enoyl-CoA reductase;Pecr
Far1	Fatty acyl-CoA reductase 1;Far1
LOC100359539	Protein LOC100359539
Ppp1cc	Serine/threonine-protein phosphatase PP1-gamma catalytic subunit;Ppp1cc
Eno2	Gamma-enolase;Eno2
Lrp1	Protein Lrp1
Hibadh	3-hydroxyisobutyrate dehydrogenase, mitochondrial;Hibadh
Acsl4	Long-chain-fatty-acid--CoA ligase 4;Acsl4
Akr7a2	Aflatoxin B1 aldehyde reductase member 2;Akr7a2

Table S1E

Proteins preferentially recovered in ASML-wt TEX

	ASML-wt TEX		-cld7kd TEX		-cld7mPalm TEX		
**Synonym**	**Prot.M**	**Pept.M**	**Prot.M**	**Pept.M**	**Prot.M**	**Pept.M**	**Protein Description**
CD166	34	12	1	1			CD166, activated leukoc. adhesion molecule, Alcam
Clic1	25	7					chloride intracellular channel 1
Ctps1	13	4					CTP synthase 1
Dcps	31	9	5	2	5	2	decapping enzyme, scavenger
Eif2s1	41	11	13	4	19	6	eukaryotic translation initiation factor 2 subunit 1
Eif2s3x	28	6					eukaryotic translation initiation factor 2, subunit 3
Hist1h2b	279	8					histone H2B
Ide	52	11	14	4	17	5	insulin-degrading enzyme
Itga2	13	8	2	2			integrin alpha-2
Kb15	41	5					keratin, type II cytoskeletal cochleal
Krt6	70	12	35	3	39	3	keratin, type II cytoskeletal 6A
LOC100910554	53	5					histone H2A type 1-like
LOC102552480	255	6					histone H2B type 2-E
Muc4	15	9	2	1			mucin 4
Myo10	54	10					myosin-10
Psmd12	24	13	9	4	6	4	26S proteasome non-ATPase regulatory subunit 12
Ras	38	6					ras protein
rCG33456c	72	18					rCG33456, isoform CRA_c
Rdx	102	9					radixin
Rpa1	19	8	1	1	2	1	replication protein A 70 kDa DNA-binding subunit
Tuba8	79	5					tubulin alpha-8 chain

Table S1F

Proteins preferentially recovered in ASML-wt and ASML-cld7mPalm TEX

	ASML-wt TEX		-cld7kd TEX		-cld7mPalm TEX		
**Synonym**	**Prot.M**	**Pept.M**	**Prot.M**	**Pept.M**	**Prot.M**	**Pept.M**	**Protein Description**
Hspa1a	304	24			541	29	dnaK-type molecular chaperone hsp72-ps1
Lta4h	15	7			73	15	Leukotriene A-4 hydrolase
Mdh2	79	12			114	14	malate dehydrogenase, mitochondrial
rCG26466	25	5			19	3	rCG26466, isoform CRA_b
Rpl7	13	8			22	8	ribosomal protein L7
Tsnax	29	11	7	5	21	10	translin-associated protein X

Table S1G

Proteins preferentially recovered in ASML-wt and ASML-cld7kd TEX

	ASML-wt TEX		-cld7kd TEX		-cld7mPalm TEX		
**Synonym**	**Prot.M**	**Pept.M**	**Prot.M**	**Pept.M**	**Prot.M**	**Pept.M**	**Protein Description**
Actn1	46	13	43	11			alpha-actinin-1
Eif3t	33	8	47	10			eukaryotic translation initiat. factor 3, subunit 10 theta
Ggt1	11	4	13	5			gamma-glutamyl transpeptidase
H2afx	96	6	82	6			H2A histone family, member X
Hsp71	725	34	582	29			heat shock protein71
Krt14	45	10	32	6			keratin, type I cytoskeletal 14
Krt16	34	8	18	3			keratin, type I cytoskeletal 16
Krt17	45	12	34	9			keratin, type I cytoskeletal 17
Krt42	41	11	24	5			keratin, type I cytoskeletal 42
Krt5	57	12	33	6			keratin, type II cytoskeletal 5
Lgals3bp	52	11	88	14	2	1	galectin-3-binding protein
Psat1	8	3	9	5			phosphoserine aminotransferase
PVR	8	5	4	2			tumor-associated glycoprotein E4, poliovirus receptor
rCG30479	11	5	18	6			rCG30479, isoform CRA_a
rCG47614	84	4	165	4			rCG47614, isoform CRA_f
Rtn4	8	3	12	6			reticulon-4
S100a14	8	5	5	3			S100 calcium binding protein A14
Tuba1b	129	12	186	13			tubulin alpha

Table S1H
Proteins enriched in ASML-wt compared to -cld7kd and -cld7mP TEX

**ER to Golgi Anterograde Transport** (R-RNO-199977)	
Copb1	Coatomer subunit beta;Copb1
Sptbn1	Protein Sptbn1
Sptan1	Spectrin alpha chain, non-erythrocytic 1;Sptan1
Uso1	General vesicular transport factor p115;Uso1
Ykt6	Synaptobrevin homolog YKT6
Ctsz	Cathepsin Z;Ctsz
Sec13	Protein SEC13
**Transport to the Golgi and subsequent modification** (R-RNO-948021)	
Copb1	Coatomer subunit beta;Copb1
Sptbn1	Protein Sptbn1
Sptan1	Spectrin alpha chain, non-erythrocytic 1;Sptan1
Uso1	General vesicular transport factor p115;Uso1
Ykt6	Synaptobrevin homolog YKT6
Ctsz	Cathepsin Z;Ctsz
Sec13	Protein SEC13 homolog
**Membrane Trafficking** (R-RNO-199991)	
Copb1	Coatomer subunit beta;Copb1
Sptbn1	Protein Sptbn1
Sptan1	Spectrin alpha chain, non-erythrocytic 1;Sptan1
Uso1	General vesicular transport factor p115;Uso1
Ykt6	Synaptobrevin homolog YKT6
Rab18	Ras-related protein Rab-18;Rab18
Ctsz	Cathepsin Z;Ctsz
Dnm1	Dynamin-1;Dnm1
Snx2	Protein Snx2
Cltc	Clathrin heavy chain 1;Cltc
Sec13	Protein SEC13
**Vesicle-mediated transport** (R-RNO-5653656)	
Copb1	Coatomer subunit beta;Copb1
Sptbn1	Protein Sptbn1
Sptan1	Spectrin alpha chain, non-erythrocytic 1;Sptan1
Uso1	General vesicular transport factor p115;Uso1
Ykt6	Synaptobrevin homolog YKT6
Rab18	Ras-related protein Rab-18;Rab18
Ctsz	Cathepsin Z;Ctsz
Dnm1	Dynamin-1;Dnm1
Snx2	Protein Snx2
Cltc	Clathrin heavy chain 1;Cltc
Sec13	Protein SEC13

Table S1I

Higher protein recovery in ASML cells versus TEX

	ASML-wt cells		ASML-wt TEX			ASML-wt cells		ASML-wt TEX	
**Synonym**	**Prot.M**	**Pept.M**	**Prot.M**	**Pept.M**	**Synonym**	**Prot.M**	**Pept.M**	**Prot.M**	**Pept.M**
Ahnak	3921	581			Ptgr1	154	37	10	5
Dync1h1	1988	478			Actn2	152	41		
Hsp90ab1	1557	138			Nnt	152	51		
Actb	1540	72			Tubb3	149	17		
Flna	1499	312	165	38	P4hb	147	59		
Myh9	1293	252	315	55	LOC100363782	145	23		
Fasn	1262	228	28	9	Ywhaq	145	42		
Sptan1	905	313	80	28	AI314180	144	78		
Sptbn1	855	268	20	11	Arf4	142	28		
Tubb4b	722	77			ASCT2	140	20		
Tubb5	714	77			Hyou1	138	55	3	1
Actn1	588	144	43	11	Arhgdia	136	31		
Iqgap1	588	202	87	31	Rpn1	134	44		
Hspd1	576	102			Snd1	134	60	40	11
Actc1	563	39			Trim28	134	43		
Myof	559	222	67	29	Gnb2l1	133	49		
Myh10	494	217	179	38	Ywhaz	133	42	38	4
Tuba4a	471	59			Golgb1	132	82		
Rps27a	469	26			Atic	130	65		
Tln1	455	151			Uggt1	130	70		
Flnb	451	172	6	3	Pdia3	129	47	4	3
Igf2r	408	145	21	14	Ahcy	128	53		
Ubr4	374	188			Hnrpk protein	127	38		
Tubb6	364	50			JH06_4067	127	34		
Akap9	340	180			Fancd2	126	18		
Actbl2	326	27			Mthfd1l	126	47		
Eif5a	322	33			Ppib	124	39	14	5
Gcn1l1	319	120			Sec31a	124	53		
Gstp1	316	33			HUWE1	123	57		
Akr1b8	299	55	11	4	Arf5	122	33		
Eprs	289	121	76	15	Kif5b	120	63		
Plec	277	148	5	4	Rock2	119	40	2	2
Fam49b	275	37			Gart	117	59		
Tpr	266	82			Iars	115	58		
P97621	261	10			Macf1	113	60		
Pdcd6	258	89			Pdia4	111	45		
Copa	255	72	8	4	Ncl	110	42	2	2
Slc25a4	253	37			Rps3	109	44		
Slc25a5	249	39			Stmn1	109	40		
Tmed10	230	26			Uso1	108	42	4	3
Ywhae	228	53			Prmt1	107	35		
Chd4	215	81	5	2	Lrrc59	106	27		
Atp5a1	213	64	4	3	Rab8a	106	16		
Lrpprc	208	111			Fabp5	105	21		
Mthfd1	206	64			Usp9x	105	55		
Hsph1	205	66			Hnrnpa2b1	103	32		
Pgam1	197	50			Ap2b1	102	37		
Stip1	195	68			Aldh2	100	43		
Rnf213	192	108			Psmd1	100	50	16	8
Tagln2	189	31	9	4	LOC102551071	99	24		
Vcp	188	73			LOC681718	99	28		
Vdac1	188	43	7	4	Psat1	99	46	9	5
Eif4g1	187	76			Rpn2	98	32		
Txn	179	17			Smc3	97	50		
Atp2a2	174	45	6	2	Esyt1	96	43		
Trap1	174	47			Lrba	95	41		
Rdx	171	48	102	9	Map4	95	40		
Slc25a3	167	29			RGD1561636	95	16		
Birc6	158	64			Tars	95	50	3	3
Gm11214	155	6			Mob1a	94	28		
Arf3	154	33			RpS12	94	19		
Gars	154	63			Paics	93	49		

Table S1J

Higher protein recovery in ASML-wt TEX than cells

	ASML-wt TEX		ASML-wt cells			ASML-wt TEX		ASML-wt cells	
Synonym	Prot.M	Pept.M	Pept.M	Synonym	Synonym	Prot.M	Pept.M	Pept.M	Synonym
LOC100910366	982	35			Fdps	50	10		
Mvp	747	99	31	16	Serinc5	43	7		
Mfge8	494	64			Il24	42	13		
Akr2	466	40			Cldn7	41	6		
Ptgfrn	446	91			Cldn4	40	6		
Htra1	426	48			Atp6ap2	39	14		
CD9	416	30	21	3	Dera	39	14		
JH06 5301	376	51			F5	36	15		
Sdcbp	337	16			Mfi2	33	17		
Mat1a	330	39			Sdcbp2	32	7		
Ubb	316	14			Slc44a2	31	9		
Asns	240	32	8	3	Plod1	29	9		
Clu	239	34			rCG30479	29	11		
rCG47614	229	8			CD59	28	9		
Asl	226	34	5	3	Tsnax	28	15	9	3
Tspan8	222	16	5	3	Clic1	25	7		
Itga6	199	82	28	17	Fbxo6	25	12	2	2
Itgb1	189	63	28	18	transmembr.pr.	25	6		
Ppp1r7	152	35	12	7	Tsta	25	10	2	2
Ubc	145	7			CD151	24	8		
Lgals3bp	142	26			Ggt1	24	9		
CD82	140	25			JH06 4591	24	11		
C3	134	22			M6pr	24	7		
Fn1	133	57			Thbs1	24	11	3	2
JH06 2394	132	31			Brox	22	6		
Itga3	126	48	23	9	Col18a1	22	8		
rCG27764	123	33			LOC100911244	22	6		
LOC100910688	121	22			rCG38382	22	12		
Grwd1	118	29			Tinagl1	21	10		
rCG58353	118	17			Dnm1	18	6		
Ist1	117	20			Pbdc1	17	6		
CD63	114	18			Tspan4	17	6		
Epcam	99	15	4	3	Adh1	16	9		
Itih2	95	6			Aqp4	16	6		
Prom1	93	31			Uxs1	16	7		
Adam10	85	21			Cldn9	14	6		
Tspan6	85	12			LOC102551877	14	7		
F2	84	15			rCG24688	14	6		
rCG55067	84	13			rCG30666	14	9		
Plekhb2	82	25			rCG55135	14	7		
Serpinf1	81	6			Itgav	11	6		
Plscr3	79	14			Hba1	10	2		
Cds1	75	11			Jam	10	6		
Itgb4	74	42	13	8	Ptprf	10	6		
rCG33456	72	18			Adam17	9	6		
Fth1	69	12			Adh3	9	6		
Nop2	67	16			Clta	9	6		
rCG24640	65	17			Adam9	8	6		
Ppp2ca	61	11			Cdhr1	8	6		
Rplp2	60	19			Itgb3	8	6		
LOC102551657	58	15			Jup	8	6		
Ttyh3	53	6			C4	7	6		
rCG42612	52	12			Itga2b	7	6		
Serpinc1	52	11			Fgb	6	6		
Vps28	52	15	2	2	Slc12a1	6	3		
Tspan14	51	13							

Table S1K

Higher protein recovery in ASML-wt TEX than ASML-cld7kd cells

	ASML-wt TEX		-cld7kd cells	
**Synonym**	**Prot.M**	**Pept.M.**	**Prot.M**	**Pept.M.**
Hspa1a	304	24	41	4
Hist1h2b	279	8		
LOC102552480	255	6		
Ptgfrn	180	30		
Eno1	158	15	38	7
Mvp	152	29	8	4
Htra1	151	16		
JH06 5301	119	16		
Mfge8	115	20		
Eef1a1	110	12		
Sdcbp	98	5		
Anxa6	90	26	10	4
Sept7	82	8	18	4
Itgb1	81	19	2	2
Eif3l	74	14	18	7
CD9	72	6	8	3
Itga6	70	20	4	2
Krt6	70	12	31	4
Tspan8	64	5	1	1
Myo10	54	10		
C3	51	8		
Ftl1	50	8		
Epcam	46	6		
Psmd6	45	18	7	4
Krt17	45	12	12	5
Ist1	44	8		
Itga3	44	13	3	2
F2	42	6		
Eif3m	42	11	6	3
Krt42	41	11		
Kb15	41	5		
Mapk1	41	10	7	5
Ras	38	6		
Itih2	37	2		
Idh1	37	11	6	3
Asl	36	8	1	1
CD166	34	12		
Krt16	34	8		
Eif3t	33	8		
JH06 2394	32	8		
Fth1	32	4		
Anxa3	32	12	14	5
Dcps	31	9	1	1
Psmd7	31	10	9	5
Fdps	30	6		
Serpinf1	30	2		
H2afy	29	6		
Tsnax	29	11	3	1
Eif2s3x	28	6		
Sept9	26	6	3	3
Clic1	25	7		
Apob	25	8	4	2
Cops3	25	10	6	4
LOC102551657	24	5		
CD63	24	4		
Krt15	22	4		
Adam10	21	7		
Il24	21	5		
Lypla1	21	4	2	1
Cops6	21	9	5	3
Ppp2ca	20	6		
Eef1e1	20	5		
Ehd3	20	3		
Krt76	20	3		
Hba2	20	2		
Cops4	20	11	7	5
Asns	19	5	2	1
Rpa1	19	8	4	3
LOC100910688	18	5		
Fn1	17	8		
Prom1	17	8		
CD59	17	3		
Clic1b	17	3		
Serpinb6	17	6	2	1
Psmb5	17	8	5	2
Thbs1	16	6		
Cds1	16	4		
Muc4	15	9		
Top2a	15	5	2	1
LOC100910308	14	5		
Plekhb2	14	5		
Itga2	13	8		
Rpl7	13	8		
Dctn3	13	4		
Lum	13	2		
Brox	12	4		
Cldn4	12	2		
Hexb	12	4	2	1
Fam175b	11	5		
Ggt1	11	4		
Tspan14	11	4		
Tspan1	11	2		
Slc44a1	11	4	2	1
Itgb4	10	8		
LOC102551877	10	6		
Rplp2	10	5		
Plscr3	10	3		
Hba1	10	2		
Tspan4	10	2		
Ttyh3	10	2		
Coro1c	10	5	1	1

Table S2

Distinct recovery of proteins in SW948-wt versus -cld7kd cells and TEX

Suppl.Table 2A

Higher recovery of proteins in SW948-wt than -cld7kd cells

	SW948-wt cells		-cld7kd cells		
**Synonym**	**Prot.M**	**Pept.M**	**Prot.M**	**Pept.M**	**Protein Description**
**RRBP1**	35	34	5	5	Ribosome-binding protein 1
**IGF2R**	38	32	8	8	Cation-independent mannose-6-phosphate receptor
**NUMA1**	30	27	7	7	Nuclear mitotic apparatus protein 1
**TPR**	30	25	6	6	Nucleoprotein TPR
**ITPR3**	27	24	5	4	Inositol 1,4,5-trisphosphate receptor type 3
**HNRNPUL2**	19	18	1	1	Heterogeneous nuclear ribonucleoprotein U-like protein 2
**CTNNB1**	19	16	2	2	Catenin beta-1
**ATP2A2**	17	15			Sarcoplasmic/endoplasmic reticulum calcium ATPase 2
**MATR3**	18	15	1	1	Matrin-3
**HNRNPL**	15	14	2	2	Heterogeneous nuclear ribonucleoprotein L
**MYO1B**	14	14	3	3	Unconventional myosin-Ib
**HNRNPR**	13	13			Heterogeneous nuclear ribonucleoprotein R
**MYH11**	16	13			Myosin-11
**HNRNPA3**	12	12	2	3	Heterogeneous nuclear ribonucleoprotein A3
**SFPQ**	12	12	5	4	Splicing factor, proline- and glutamine-rich
**STT3A**	14	12	5	4	Oligosaccharyltransferase complex, catalytic subunit
**UGT1A6**	13	12			UDP-glucuronosyltransferase 1-6
**USO1**	14	12	3	4	General vesicular transport factor p115
**SMARCA4**	15	11	4	3	Transcription activator BRG1
**ACO2**	13	10	1	1	Aconitate hydratase, mitochondrial
**AKR1C1**	13	10			Aldo-keto reductase family 1 member C1
**CIT**	11	10	2	1	Citron Rho-interacting kinase
**DDX17**	12	10	2	1	Probable ATP-dependent RNA helicase DDX17
**HNRNPC**	10	10	1	1	Heterogeneous nuclear ribonucleoproteins C1/C2
**JUP**	12	10	2	1	Junction plakoglobin
**LBR**	10	10	2	3	Lamin-B receptor
**OGDH**	11	10	2	2	2-oxoglutarate dehydrogenase, mitochondrial
**OLFM4**	10	10			Olfactomedin-4
**PRKCSH**	11	10	3	3	Glucosidase 2 subunit beta
**SMC1A**	14	10	5	3	Structural maintenance of chromosomes protein 1A
**STT3B**	12	10	3	3	Oligosaccharyltransferase complex, catalytic subunit
**COTL1**	10	9	3	3	Coactosin-like protein
**DDX5**	12	9			Probable ATP-dependent RNA helicase DDX5
**DDX5**	12	9	2	3	Probable ATP-dependent RNA helicase DDX5
**LAMA5**	11	9	1	1	Laminin subunit alpha-5
**MVP**	13	9	2	2	Major vault protein
**MYO1C**	11	9	2	2	Unconventional myosin-Ic
**PRMT1**	11	9	3	3	Protein arginine N-methyltransferase 1
**YBX1**	10	9	3	3	Nuclease-sensitive element-binding protein 1
**AKR1C2**	12	8			Aldo-keto reductase family 1 member C2
**FLNC**	9	8			Filamin-C
**MTHFD1L**	9	8	2	2	Monofunctional C1-tetrahydrofolate synthase, mitochondrial
**TRRAP**	9	8			Transformation/transcription domain-associated protein
**ERLIN2**	8	7			Erlin-2
**HSD17B11**	7	7	1	1	Estradiol 17-beta-dehydrogenase 11
**HTT**	9	7	3	2	Huntingtin
**PABPC4**	9	7			Polyadenylate-binding protein 4
**PLXNB2**	8	7			Plexin-B2
**PNPT1**	9	7	3	2	Polyribonucleotide nucleotidyltransferase 1, mitochondrial
**RALY**	8	7			RNA-binding protein Raly
**RIF1**	8	7	1	1	Telomere-associated protein RIF1
**UGT1A7**	7	7			UDP-glucuronosyltransferase 1-7
**ARMC10**	6	6	2	2	Armadillo repeat-containing protein 10
**CD166**	6	6	1	1	CD166 antigen ALCAM
**CDH1**	6	6	2	2	Cadherin-1
**CUL4A**	6	6	2	2	Cullin-4A
**EGFR**	7	6	1	1	Epidermal growth factor receptor
**EPHX1**	6	6			Epoxide hydrolase 1
**FTH1**	7	6	1	1	Ferritin heavy chain
**HACD3**	6	6	1	1	Very-long-chain (3R)-3-hydroxyacyl-CoA dehydratase 3

Table S2A cont.

Synonym	SW948-wt cells Prot.M	Pept.M	-cld7kd cells Prot.M	Pept.M	Protein Description
**DIE**	8	6	2	2	Insulin-degrading enzyme
**ITGB1**	8	6	2	1	Integrin beta-1
**LAMC1**	6	6	1	1	Laminin subunit gamma-1
**MRPL17**	7	6	2	1	39S ribosomal protein L17, mitochondrial
**NDUFS5**	7	6			NADH dehydrogenase [ubiquinone] iron-sulfur protein 5
**NUP210**	8	6	1	1	Nuclear pore membrane glycoprotein 210
**PYCR1**	6	6	1	0	Pyrroline-5-carboxylate reductase 1, mitochondrial
**REEP6**	6	6	1	1	Receptor expression-enhancing protein 6
**SEPT7**	6	6	2	2	Septin-7
**TM9SF2**	10	6	2	2	Transmembrane 9 superfamily member 2
**UBE2L3**	6	6	2	2	Ubiquitin-conjugating enzyme E2 L3
**ARPC1B**	5	5	2	1	Actin-related protein 2/3 complex subunit 1B
**DPEP1**	6	5			Dipeptidase 1
**EPHB2**	5	5	1	1	Ephrin type-B receptor 2
**ERO1A**	8	5	1	1	ERO1-like protein alpha
**FANCI**	6	5			Fanconi anemia group I protein
**HK2**	6	5	2	1	Hexokinase-2
**HM13**	7	5			Minor histocompatibility antigen H13
**HNRNPH2**	8	5			Heterogeneous nuclear ribonucleoprotein H2
**HNRNPH3**	6	5			Heterogeneous nuclear ribonucleoprotein H3
**HSPB1**	5	5			Heat shock protein beta-1
**ILF2**	5	5	1	1	Interleukin enhancer-binding factor 2
**LAMB1**	8	5	1	1	Laminin subunit beta-1
**LSM4**	6	5	2	1	U6 snRNA-associated Sm-like protein LSm4
**MRPL19**	6	5	2	1	39S ribosomal protein L19, mitochondrial
**MRPL22**	6	5	1	1	39S ribosomal protein L22, mitochondrial
**MRPL43**	6	5	2	1	39S ribosomal protein L43, mitochondrial
**MUC13**	5	5	1	1	Mucin-13
**NDUFS2**	6	5	2	1	NADH dehydrogenase [ubiquinone] iron-sulfur protein 2, mitochondrial
**PCBP3**	5	5			Poly(rC)-binding protein 3
**PFN2**	5	5	1	1	Profilin-2
**PUS7**	5	5			Pseudouridylate synthase 7 homolog
**SEC63**	6	5	2	1	Translocation protein SEC63 homolog
**SELENOF**	5	5			Selenoprotein F
**SLC35A4**	6	5			SLC35A4 upstream open reading frame protein
**SRRT**	8	5	2	1	Serrate RNA effector molecule homolog
**SRSF3**	5	5			Serine/arginine-rich splicing factor 3
**STK26**	7	5	1	1	Serine/threonine-protein kinase 26
**DNPH1**	4	4	1	1	2'-deoxynucleoside 5'-phosphate N-hydrolase 1
**DUT**	4	4	1	1	Deoxyuridine 5'-triphosphate nucleotidohydrolase, mitochondrial
**FRIL**	5	4			Ferritin light chain FTL
**GFPT2**	5	4			Glutamine--fructose-6-phosphate aminotransferase [isomerizing] 2
**GHITM**	4	4	1	1	Growth hormone-inducible transmembrane protein
**GOLGA4**	6	4	1	1	Golgin subfamily A member 4
**H2AV**	4	4			Histone H2A.V H2AFV
**HARS**	5	4	1	1	Histidine--tRNA ligase, cytoplasmic
**HNRNPAB**	5	4			Heterogeneous nuclear ribonucleoprotein A/B
**ILF3**	5	4			Interleukin enhancer-binding factor 3
**ITPR2**	6	4			Inositol 1,4,5-trisphosphate receptor type 2
**KDM1A**	6	4			Lysine-specific histone demethylase 1A KDM1A
**KRAS**	4	4			GTPase KRas
**LCN2**	5	4	1	1	Neutrophil gelatinase-associated lipocalin
**LNPEP**	4	4			Leucyl-cystinyl aminopeptidase
**MBOAT7**	4	4	1	1	Lysophospholipid acyltransferase 7
**OAT**	5	4			Ornithine aminotransferase, mitochondrial
**PPM1G**	4	4	1	1	Protein phosphatase 1G
**PRPS1**	5	4			Ribose-phosphate pyrophosphokinase 1

Table S2A cont.

	SW948-wt cells		-cld7kd cells		
**Synonym**	**Prot.M**	**Pept.M**	**Prot.M**	**Pept.M**	**Protein Description**
**PTCD3**	4	4	1	1	Pentatricopeptide repeat domain-containing protein 3, mitochondrial
**RAB25**	4	4			Ras-related protein Rab-25
**SPTBN2**	11	4			Spectrin beta chain, non-erythrocytic 2
**SRSF1**	4	4			Serine/arginine-rich splicing factor 1
**TMED3**	4	4			Transmembrane emp24 domain-containing protein 3
**TRIP11**	5	4			Thyroid receptor-interacting protein 11
**WFS1**	5	4	1	1	Wolframin
**ABCC3**	4	3			Canalicular multispecific organic anion transporter 2
**ACTR1A**	3	3			Alpha-centractin
**ADD1**	4	3			Alpha-adducin
**ARL8A**	4	3			ADP-ribosylation factor-like protein 8A
**ATXN10**	3	3			Ataxin-10
**CSKP**	3	3			Peripheral plasma membrane protein CASK CASK
**CYP51A1**	6	3			Lanosterol 14-alpha demethylase
**ERH**	3	3			Enhancer of rudimentary homolog
**ERMP1**	4	3			Endoplasmic reticulum metallopeptidase 1
**FSCN1**	4	3			Fascin
**HMGCS1**	3	3			Hydroxymethylglutaryl-CoA synthase, cytoplasmic
**HNRNPA0**	4	3			Heterogeneous nuclear ribonucleoprotein A0
**HNRNPDL**	3	3			Heterogeneous nuclear ribonucleoprotein D-like
**LSM1**	3	3			U6 snRNA-associated Sm-like protein LSm1
**MRPL28**	4	3			39S ribosomal protein L28, mitochondrial
**PEA-15**	3	3			Astrocytic phosphoprotein
**POLR2B**	3	3			DNA-directed RNA polymerase II subunit RPB2
**PTDSS1**	4	3			Phosphatidylserine synthase 1
**RAB6B**	5	3			Ras-related protein Rab-6B
**RBBP9**	4	3			Putative hydrolase RBBP9
**RBMX**	3	3			RNA-binding motif protein, X chromosome
**RHOB**	4	3			Rho-related GTP-binding protein RhoB
**SAFB**	5	3			Scaffold attachment factor B1
**SELH**	3	3			Selenoprotein H
**SLC16A3**	4	3			Monocarboxylate transporter 4
**SNX2**	3	3			Sorting nexin-2
**TRA2B**	3	3			Transformer-2 protein homolog beta
**TROVE2**	3	3			60 kDa SS-A/Ro ribonucleoprotein

Table S2B

Higher recovery of proteins in SW948-cld7kd than -wt cells

	SW948-wt cells		-cld7kd cells		
**Synonym**	**Prot.M**	**Pept.M**	**Prot.M**	**Pept.M**	**Protein Description**
**AHNAK**	21	14	84	83	Neuroblast differentiation-associated protein AHNAK
**CNOT1**	3	2	14	14	CCR4-NOT transcription complex subunit 1
**ECM29**	9	6	19	19	Proteasome-associated protein ECM29 homolog
**IQGAP2**			13	13	Ras GTPase-activating-like protein IQGAP2
**PA2G4**	7	5	11	13	Proliferation-associated protein 2G4
**ACTN2**			11	12	Alpha-actinin-2
**PSAT1**	5	5	11	12	Phosphoserine aminotransferase
**ATP5EP2**	3	2	10	10	ATP synthase subunit epsilon-like protein, mitochondrial
**DNAJC13**			9	10	DnaJ homolog subfamily C member 13
**DNM2**	6	3	8	9	Dynamin-2
**OSBP**	5	4	9	9	Oxysterol-binding protein 1
**RL7**	1	1	9	9	60S ribosomal protein L7 RPL7
**HSPA1L**			9	9	Heat shock 70 kDa protein 1-like
**KRT4**	6	4	9	9	Keratin, type II cytoskeletal 4
**EIF3L**	3	2	9	8	Eukaryotic translation initiation factor 3 subunit L
**TLN2**			7	8	Talin-2
**PFAS**	5	3	8	8	Phosphoribosylformylglycinamidine synthase
**ANXA11**	3	2	7	7	Annexin A11
**KRT14**			7	7	Keratin, type I cytoskeletal 14
**PC**	3	2	7	7	Pyruvate carboxylase, mitochondrial
**EIF3M**	4	3	6	7	Eukaryotic translation initiation factor 3 subunit M
**LTA4H**	4	3	6	7	Leukotriene A-4 hydrolase
**EIF3I**	6	2	7	7	Eukaryotic translation initiation factor 3 subunit I
**LAP2A**	2	1	6	7	Lamina-associated polypeptide 2, isoform alpha
**PAK1**			6	7	Serine/threonine-protein kinase PAK 1
**PPP2CA**	5	3	6	7	Serine/threonine-protein phosphatase 2A catalytic subunit alpha isoform
**AP3B1**	5	3	7	6	AP-3 complex subunit beta-1
**ASS1**			6	6	Argininosuccinate synthase
**CUL1**	2	2	6	6	Cullin-1
**CUL3**	2	1	6	6	Cullin-3
**IPO4**	4	2	5	6	Importin-4
**KIF13B**	6	3	5	6	Kinesin-like protein KIF13B
**NANS**	3	3	6	6	Sialic acid synthase
**POF1B**	2	1	5	6	Protein POF1B
**RPS11**	6	3	6	6	40S ribosomal protein S11
**RPS3A**	2	2	6	6	40S ribosomal protein S3a
**RPS4X**	5	3	6	6	40S ribosomal protein S4, X isoform
**TKFC**	3	3	5	6	Triokinase/FMN cyclase
**UBR5**	1	1	6	6	E3 ubiquitin-protein ligase UBR5
**ABCE1**	4	2	6	5	ATP-binding cassette sub-family E member 1
**AKR1A1**	2	2	5	5	Alcohol dehydrogenase [NADP(+)]
**COASY**	3	2	5	5	Bifunctional coenzyme A synthase
**FUBP3**	3	2	5	5	Far upstream element-binding protein 3
**NAP1L4**	4	2	5	5	Nucleosome assembly protein 1-like 4
**PDLIM1**	2	1	5	5	PDZ and LIM domain protein 1
**PYCR2**	2	2	5	5	Pyrroline-5-carboxylate reductase 2
**RPA1**			6	5	Ribose-5-phosphate isomerase
**RPL10**			5	5	60S ribosomal protein L10
**RPL10A**	1	1	6	5	60S ribosomal protein L10a
**RPL23A**	2	1	5	5	60S ribosomal protein L23a
**RPL6**			6	5	60S ribosomal protein L6
**PPP1CB**			5	5	Serine/threonine-protein phosphatase PP1-beta catalytic subunit
**TXNL1**	2	2	5	5	Thioredoxin-like protein 1
**UBE2O**			6	5	(E3-independent) E2 ubiquitin-conjugating enzyme
**ACSL3**	4	2	4	5	Long-chain-fatty-acid--CoA ligase 3
**ADH5**	2	1	6	5	Alcohol dehydrogenase class-3
**AIP**	2	1	4	5	AH receptor-interacting protein
**APEX1**	4	1	6	5	DNA-(apurinic or apyrimidinic site) lyase
**C12orf10**			4	5	Chromosome C12 orf10
**DDAH2**	1	1	4	5	N(G),N(G)-dimethylarginine dimethylaminohydrolase 2
**LAP2B**	3	2	4	5	Lamina-associated polypeptide 2, isoforms beta/gamma
**POTEF**			5	5	POTE ankyrin domain family member F

Table S2B cont.

	SW948-wt cells		-cld7kd cells		
**Synonym**	**Prot.M**	**Pept.M**	**Prot.M**	**Pept.M**	**Protein Description**
**RPL26**			5	5	60S ribosomal protein L26
**RPL4**			4	5	60S ribosomal protein L4
**USP47**			6	5	Ubiquitin carboxyl-terminal hydrolase 47
**WARS**	3	2	4	5	Tryptophan--tRNA ligase, cytoplasmic
**CNN2**			5	4	Calponin-2
**DNAJB1**	1	1	4	4	DnaJ homolog subfamily B member 1
**EIF3E**			4	4	Eukaryotic translation initiation factor 3 subunit E
**FAM120A**			5	4	Constitutive coactivator of PPAR-gamma-like protein 1
**GRHPR**			3	4	Glyoxylate reductase/hydroxypyruvate reductase
**HDHD3**			4	4	Haloacid dehalogenase-like hydrolase domain-containing protein 3
**RAD23B**			3	4	UV excision repair protein RAD23 homolog B
**RPL18A**			3	4	60S ribosomal protein L18a
**RPL30**	2	1	3	4	60S ribosomal protein L30
**RPL7A**			5	4	60S ribosomal protein L7a
**RPL9**			3	4	60S ribosomal protein L9
**SPRYD4**	2	1	3	4	SPRY domain-containing protein 4
**AK1**			3	3	Adenylate kinase isoenzyme 1
**ALDOC**			3	3	Fructose-bisphosphate aldolase C
**GAPVD1**			3	3	GTPase-activating protein and VPS9 domain-containing protein 1
**HSPA4L**			3	3	Heat shock 70 kDa protein 4L
**KRT77**			3	3	Keratin, type II cytoskeletal 1b
**NTMT1**			3	3	N-terminal Xaa-Pro-Lys N-methyltransferase 1
**PPP2R1B**			3	3	Serine/threonine-protein phosphatase subunit A beta
**PYGL**			3	3	Glycogen phosphorylase, liver form
**RPL13**			3	3	60S ribosomal protein L13
**RPL13A**			3	3	60S ribosomal protein L13a
**RPL15**			3	3	60S ribosomal protein L15
**RPL36A**			3	3	60S ribosomal protein L36a
**RPL36L**			3	3	60S ribosomal protein L36a-like
**SEC13**			3	3	Protein SEC13 homolog
**A2M**			3	3	Alpha-2-macroglobulin
**ANKFY1**			3	3	Rabankyrin-5
**ANXA7**			2	3	Annexin A7
**C9orf64**			2	3	UPF0553 protein C9orf64
**CKB**			2	3	Creatine kinase B-type
**DCTD**			3	3	Deoxycytidylate deaminase
**DYNLL2**			3	3	Dynein light chain 2, cytoplasmic
**EFL1**			3	3	Elongation factor-like GTPase 1
**GDA**			2	3	Guanine deaminase
**HSPB11**			2	3	Intraflagellar transport protein 25 homolog
**MAP2K1**			2	3	Dual specificity mitogen-activated protein kinase kinase 1
**NIPSNAP3A**			3	3	Protein NipSnap homolog 3A
**PDAP1**			2	3	28 kDa heat- and acid-stable phosphoprotein
**PDCD4**			2	3	Programmed cell death protein 4
**PEPD**			3	3	Xaa-Pro dipeptidase
**PTBP3**			2	3	Polypyrimidine tract-binding protein 3
**RPL14**			2	3	60S ribosomal protein L14
**RPL28**			3	3	60S ribosomal protein L28
**RPL3**			2	3	60S ribosomal protein L3
**RPL34**			3	3	60S ribosomal protein L34
**RPL35A**			3	3	60S ribosomal protein L35a
**RPL36**			2	3	60S ribosomal protein L36
**RPL37A**			3	3	60S ribosomal protein L37a
**RPLP2**			2	3	60S acidic ribosomal protein P2
**STXBP2**			3	3	Syntaxin-binding protein 2
**TCERG1**			3	3	Transcription elongation regulator 1

Table S2C

Higher recovery of proteins in SW948-wt than SW948-cld7kd TEX

	SW948-wt TEX		-cld7kd TEX		
**Synonym**	**Prot.M**	**Pept.M**	**Prot.M**	**Pept.M**	**Protein Description**
**ADA**	5	3			Adenosine deaminase
**AKR1C3**	15	5			Aldo-keto reductase family 1 member C3
**ANXA6**	15	7	6	3	Annexin A6
**AOC1**	20	12	8	5	Amiloride-sensitive amine oxidase
**ACTR1B**	16	6	7	3	Beta-centractin
**APEH**	7	3			Acylamino-acid-releasing enzyme
**ARPC1B**	8	4			Actin-related protein 2/3 complex subunit 1B
**ATP6V1B2**	9	4			V-type proton ATPase subunit B, brain isoform
**CAPN1**	6	4			Calpain-1 catalytic subunit
**COPS5**	12	8	8	4	COP9 signalosome complex subunit 5
**CSNK2B**	6	4	1	1	Casein kinase II subunit beta
**DDX39B**	6	3			Spliceosome RNA helicase DDX39B
**EIF4E2**	6	3			Eukaryotic translation initiation factor 4E type 2
**EPS8L3**	49	15	4	3	Epidermal growth factor receptor kinase substrate 8-like protein 3
**GC**	13	6	8	3	Vitamin D-binding protein
**GLB1**	31	6	6	3	Beta-galactosidase
**GLG1**	8	5	4	2	Golgi apparatus protein 1
**GSN**	10	6	5	2	Gelsolin
**GSS**	11	7	4	3	Glutathione synthetase
**H2AFY**	17	6	2	1	Core histone macro-H2A.1
**HSD17B4**	10	6	1	1	Peroxisomal multifunctional enzyme type 2
**HSPA4**	10	7	10	3	Heat shock 70 kDa protein 4
**IGF2R**	42	25	19	12	Cation-independent mannose-6-phosphate receptor
**KRT19**	16	6	10	3	Keratin, type I cytoskeletal 19
**MCM2**	3	3			DNA replication licensing factor MCM2
**NAGLU**	6	4	1	1	Alpha-N-acetylglucosaminidase
**NARS**	17	10	6	3	Asparagine--tRNA ligase
**NEU1**	10	4			Sialidase-1
**NMT1**	6	4			Glycylpeptide N-tetradecanoyltransferase 1
**NONO**	8	4			Non-POU domain-containing octamer-binding protein
**OLFM4**	8	6			Olfactomedin-4
**PAICS**	18	10	10	5	Multifunctional protein ADE2
**PARP4**	15	8	2	1	Poly [ADP-ribose] polymerase 4
**PDXK**	5	4	2	1	Pyridoxal kinase
**PGM1**	5	3			Phosphoglucomutase-1
**PPP2CA**	10	5	4	2	Serine/threonine-protein phosphatase 2A alpha
**PSME1**	9	4	1	1	Proteasome activator complex subunit 1
**PYGL**	26	7			Glycogen phosphorylase, liver form
**QSOX1**	45	11	15	3	Sulfhydryl oxidase 1
**RAB6B**	50	6			Ras-related protein Rab-6B
**RDX**	82	10			Radixin
**RNPEP**	13	6	4	3	Aminopeptidase B
**RPS27A**	315	7	3	1	Ubiquitin-40S ribosomal protein S27a
**TARS**	10	7	2	2	Valine--tRNA ligase
**TSTA3**	18	5	2	1	GDP-L-fucose synthase
**TUBA1A**	160	16			Tubulin alpha-1A chain
**TUBB2A**	90	15			Tubulin beta-2A chain
**UBE2V1**	6	4			Ubiquitin-conjugating enzyme E2 variant 1
**XPO1**	43	21	22	10	Exportin-1

Table S2D

Higher recovery of proteins in SW948-cld7kd TEX than SW948-wt TEX

	SW948-wt TEX		-cld7kd TEX		
**Synonym**	**Prot.M**	**Pept.M**	**Prot.M**	**Pept.M**	**Protein Description**
**AARS**			5	3	Alanine--tRNA ligase
**ACE**			5	3	Angiotensin-converting enzyme
**ACP2**	8	3	11	6	Lysosomal acid phosphatase
**AP2B1**	12	2	49	16	AP-2 complex subunit beta
**AREG**	7	1	26	4	Amphiregulin
**ATP1A3**			118	15	Sodium/potassium-transporting ATPase subunit alpha-3
**ATP2B4**			13	3	Plasma membrane calcium-transporting ATPase 4
**BET1L**			7	4	BET1-like protein
**BMP4**			7	4	Bone morphogenetic protein 4
**BMP7**			9	4	Bone morphogenetic protein 7
**CAPN5**	9	4	56	13	Calpain-5
**CASK**	26	8	102	25	Peripheral plasma membrane protein CASK
**CD166**	8	4	30	12	CD166 antigen
**CD276**			7	4	CD276 antigen
**CD97**	3	2	15	7	CD97 antigen
**CHMP2B**	2	1	9	4	Charged multivesicular body protein 2b
**CLDN1**			8	3	Claudin domain-containing protein 1
**CTNND1**	4	1	39	13	Catenin delta-1
**DIP2A**			24	9	Disco-interacting protein 2 homolog A
**DIP2C**			17	3	Disco-interacting protein 2 homolog C
**DLG1**	14	6	32	13	Disks large homolog 1
**DNM1L**			8	3	Dynamin-1-like protein
**DPP7**			7	3	Dipeptidyl peptidase 2
**DYNLT1**	1	1	6	4	Dynein light chain Tctex-type 1
**EIF1AX**	4	2	18	7	Eukaryotic translation initiation factor 1A, X-chromosomal
**EPB41L1**	2	1	7	4	Band 4.1-like protein 1
**EPHA2**	28	4	77	15	Ephrin type-A receptor 2
**EPHB4**			60	7	Ephrin type-B receptor 4
**EPRS**	3	3	9	6	Bifunctional glutamate/proline--tRNA ligase
**FARP1**	4	1	28	12	FERM, RhoGEF and pleckstrin domain-containing protein 1
**FARP2**			6	4	FERM, RhoGEF and pleckstrin domain-containing protein 2
**FKBP4**	6	3	14	6	Peptidyl-prolyl cis-trans isomerase FKBP4
**FLNA**			5	3	Filamin-A
**GGH**	3	2	10	6	Gamma-glutamyl hydrolase
**GNA11**	1	1	10	4	Guanine nucleotide-binding protein subunit alpha-11
**HEPH**	2	1	13	4	Hephaestin
**HRSP12**			7	4	Ribonuclease UK114
**ITGB5**	24	9	49	21	Integrin beta-5
**KRT14**			16	6	Keratin, type I cytoskeletal 14
**KRT6A**			25	6	Keratin, type II cytoskeletal 6A
**KRT77**			27	3	Keratin, type II cytoskeletal 1b
**LIPH**	7	4	12	8	Lipase member H
**LRRC57**			3	3	Leucine-rich repeat-containing protein 57
**LY75**	5	3	19	9	Lymphocyte antigen 75
**MAN2B1**			5	3	Lysosomal alpha-mannosidase
**MAP4K4**			67	11	Mitogen-activated protein kinase kinase kinase kinase 4
**MET**	4	3	12	6	Hepatocyte growth factor receptor
**MINK1**	28	9	115	22	Misshapen-like kinase 1
**MVB12A**	8	4	24	8	Multivesicular body subunit 12A
**MVB12B**	12	5	64	13	Multivesicular body subunit 12B
**MYOF**			6	4	Myoferlin
**NCBP1**			5	3	Nuclear cap-binding protein subunit 1
**PA2G4**	15	3	22	6	Proliferation-associated protein 2G4
**PKP3**	11	4	50	11	Plakophilin-3
**PLAUR**	3	2	9	5	Urokinase plasminogen activator surface receptor
**PLXNB2**	7	3	22	13	Plexin-B2
**POTEF**			118	6	POTE ankyrin domain family member F

Table S2D cont.

	SW948-wt TEX		-cld7kd TEX		
**Synonym**	**Prot.M**	**Pept.M**	**Prot.M**	**Pept.M**	**Protein Description**
**PRPF19**	8	2	15	5	Pre-mRNA-processing factor 19
**PRSS23**	4	3	30	8	Serine protease 23
**PSAT1**	1	1	6	4	Phosphoserine aminotransferase
**PSMC2**	1	1	8	5	26S protease regulatory subunit 7
**PSMD5**			4	3	26S proteasome non-ATPase regulatory subunit 5
**PTP4A2**			8	5	Protein tyrosine phosphatase type IVA 2
**PTPRA**			3	3	Receptor-type tyrosine-protein phosphatase alpha
**PTPRF**	20	13	69	29	Receptor-type tyrosine-protein phosphatase F
**RAP2A**	23	6	57	13	Ras-related protein Rap-2a
**RHOF**			5	3	Rho-related GTP-binding protein RhoF
**RHOG**	10	4	20	9	Rho-related GTP-binding protein RhoG
**ROR1**			9	3	Inact. tyrosine-protein kinase transmembrane receptor ROR1
**RPL35A**			8	4	60S ribosomal protein L35a
**RPS17**	2	1	8	4	40S ribosomal protein S17
**SARS**	8	3	8	6	Serine--tRNA ligase, cytoplasmic
**SDF4**			6	3	45 kDa calcium-binding protein
**SERPINB6**	5	4	20	8	Serpin B6
**SLC43A1**			15	3	Large neutral amino acids transporter small subunit 3
**ST14**	39	9	81	19	Suppressor of tumorigenicity 14 protein
**TLN1**			9	7	Talin-1
**TMEM2**	4	2	17	10	Transmembrane protein 2
**TNIK**	31	10	108	22	TRAF2 and NCK-interacting protein kinase
**TSPAN3**	7	1	27	4	Tetraspanin-3
**TUBA1C**			124	16	Tubulin alpha-1C chain
**UBB**			423	9	Polyubiquitin-B
**UBE2V2**			10	5	Ubiquitin-conjugating enzyme E2 variant 2
**UEVLD**	5	3	16	8	Ubiquitin-conjugating enzyme E2 variant 3
**VPS36**			7	6	Vacuolar protein-sorting-associated protein 36
**ZDHHC5**			4	3	Palmitoyltransferase ZDHHC5

Table S2E

Higher protein recovery in SW948-wt TEX than SW948-wt and -cld7kd cells

	SW948-wt cells		-cld7kd cells		SW948-wt TEX		
**Synonym**	**Prot M**	**Pept M**	**Prot M**	**Pept M**	**Prot M**	**Pept M**	**Protein Description**
A2MG			12	4	96	8	Alpha-2-macroglobulin
ACTL6A	4	2	4	2	9	5	Actin-like protein 6A
ACTR1B					16	6	Beta-centractin
ADAM10					199	21	Disintegrin and metalloproteinase domain-containing prot.10
ADAM9					15	8	Disintegrin and metalloproteinase domain-containing prot.9
ANXA6					15	7	Annexin A6
AOC1					20	12	Amiloride-sensitive amine oxidase
AP2A2					11	5	AP-2 complex subunit alpha-2
AP2M1					16	9	AP-2 complex subunit mu
APOB					75	13	Apolipoprotein B-100
ARRDC1					115	10	Arrestin domain-containing protein 1
ATP1B1	4	2	4	1	27	7	Sodium/potassium-transporting ATPase subunit beta-1
ATP6AP2					10	6	Renin receptor
ATP6V0D1					18	9	V-type proton ATPase subunit d 1
ATP6V1D					22	7	V-type proton ATPase subunit D
BROX					20	10	BRO1 domain-containing protein BROX
CASK	4	3			26	8	Peripheral plasma membrane protein CASK
CD55					27	9	Complement decay-accelerating factor
CD63	3	2	1	1	135	9	CD63 antigen
CD81	22	4	15	3	380	8	CD81 antigen
CD82					12	5	CD82 antigen
CD9	10	3	9	2	199	8	CD9 antigen
CEMIP					48	23	Cell migration-inducing and hyaluronan-binding protein
CHMP1B					13	6	Charged multivesicular body protein 1b
CHMP2A			1	1	28	8	Charged multivesicular body protein 2a
CIB1					24	10	Calcium and integrin-binding protein 1
CLD2	2	1			33	5	Claudin-2
CLDN3	10	3	5	3	97	6	Claudin-3
CLU					13	5	Clusterin
CO3					108	12	Complement C3
COPS4			2	2	12	7	COP9 signalosome complex subunit 4
COPS5	2	1	4	2	12	8	COP9 signalosome complex subunit 5
CSNK1A1					10	6	Casein kinase I isoform alpha
CXADR					27	10	Coxsackievirus and adenovirus receptor
DIP2B					196	44	Disco-interacting protein 2 homolog B
DLG1	2	1			14	6	Disks large homolog 1
DNAJA1	2	2	4	3	12	7	DnaJ homolog subfamily A member 1
DPP4	4	2	1	1	93	31	Dipeptidyl peptidase 4
DSG2	1	1	3	2	116	24	Desmoglein-2
EFNB1					28	6	Ephrin-B1
EFNB2					21	6	Ephrin-B2
EHD1	4	3	3	2	40	21	EH domain-containing protein 1
EHD4			4	3	37	17	EH domain-containing protein 4
EPCAM	35	7	36	7	288	14	Epithelial cell adhesion molecule
EPHB2	7	5	2	1	59	16	Ephrin type-B receptor 2
EPHB3					48	12	Ephrin type-B receptor 3
EPS8L3			2	2	49	15	Epidermal growth factor receptor kinase substrate 8 3
F2					32	7	Prothrombin
FA5					23	5	Coagulation factor V
FAT1	23	12	8	7	86	27	Protocadherin Fat 1
FPRP					365	43	Prostaglandin F2 receptor negative regulator
FTL	6	4	2	1	42	9	Ferritin light chain
FYN					15	5	Tyrosine-protein kinase Fyn
GAA	2	1	2	2	43	11	Lysosomal alpha-glucosidase
GLB1			4	2	31	6	Beta-galactosidase
GNAI3					24	5	Guanine nucleotide-binding protein G(k) subunit alpha
GNAS	4	2	1	1	19	6	Guanine nucleotide-binding protein G(s) subunit alpha XLas
GNB1	3	1	6	4	33	12	Guanine nucleotide-binding protein G(I)/G(S)/G(T) subu. beta1
GNB2			4	3	34	11	Guanine nucleotide-binding protein G(I)/G(S)/G(T) subu. beta-2
GNB4	3	1			14	5	Guanine nucleotide-binding protein subunit beta-4
GOLGA7	2	1	1	1	20	5	Golgin subfamily A member 7

Table S2E cont.

Synonym	SW948-wt cells Prot M	Pept M	-cld7kd cells Prot M	Pept M	SW948-wt TEX Prot M	Pept M	Protein Description
GPA33	4	2	4	2	80	10	Cell surface A33 antigen
GPRC5A					33	5	Retinoic acid-induced protein 3
GPRC5C					16	5	G-protein coupled receptor family C group 5 member C
H2AFY					17	6	Core histone macro-H2A.1
HIST1H2BB	28	3	18	3	103	6	Histone H2B type 1-B
HIST1H2BC	29	3	20	3	110	7	Histone H2B type 1-C/E/F/G/I
HIST3H3	8	2	9	3	60	6	Histone H3.1t
IGSF3					34	18	Immunoglobulin superfamily member 3
IGSF8					117	19	Immunoglobulin superfamily member 8
IST1					72	13	IST1 homolog
ITGA2					29	17	Integrin alpha-2
ITGA3					50	14	Integrin alpha-3
ITGA6	61	22	5	3	230	45	Integrin alpha-6
ITGAV	2	1	4	2	82	34	Integrin alpha-V
ITGB1	9	6	4	2	70	22	Integrin beta-1
ITGB5					24	9	Integrin beta-5
ITIH2					55	6	Inter-alpha-trypsin inhibitor heavy chain H2
ITIH3					40	5	Inter-alpha-trypsin inhibitor heavy chain H3
JAM1					28	8	Junctional adhesion molecule A F11R
JUP	20	9	1	1	100	19	Junction plakoglobin
LGALS3BP					148	20	Galectin-3-binding protein
LSR					36	8	Lipolysis-stimulated lipoprotein receptor
LYN					27	8	Tyrosine-protein kinase Lyn
MAN1A1					38	10	Mannosyl-oligosaccharide 1,2-alpha-mannosidase IA
MELTF					11	6	Melanotransferrin
MEP1A					166	21	Meprin A subunit alpha
MFGE8					76	17	Lactadherin
MINK1	3	2			28	9	Misshapen-like kinase 1
MITD1					26	10	MIT domain-containing protein 1
MUC13	16	5	7	2	174	11	Mucin-13
MVB12B					12	5	Multivesicular body subunit 12B
MVP	16	9	6	3	393	44	Major vault protein
NCSTN			2	1	14	6	Nicastrin
PARP4	9	2	3	2	15	8	Poly [ADP-ribose] polymerase 4
PDCD6IP	19	8	34	17	370	42	Programmed cell death 6-interacting protein
PLEKHB2					23	5	Pleckstrin homology domain-containing family B member 2
PLOD1					10	5	Procollagen-lysine,2-oxoglutarate 5-dioxygenase 1
PLPP2					15	5	Phospholipid phosphatase 2
PLSCR1					53	7	Phospholipid scramblase 1
PLSCR3					22	7	Phospholipid scramblase 3
PPP1R7	4	2	7	4	79	18	Protein phosphatase 1 regulatory subunit 7
PROM1					67	20	Prominin-1
PSMA3	13	7	24	7	48	14	Proteasome subunit alpha type-3
PSMB7	6	3	7	4	20	9	Proteasome subunit beta type-7
PSMB8	6	3	4	2	27	11	Proteasome subunit beta type-8
PSMD6	13	6	14	6	44	19	26S proteasome non-ATPase regulatory subunit 6
PTP4A2	8	4	4	2	11	8	Protein tyrosine phosphatase type IVA 2
QDPR	6	3	4	2	22	7	Dihydropteridine reductase
QPCT					12	6	Glutaminyl-peptide cyclotransferase
QSOX1					45	11	Sulfhydryl oxidase 1
RAB35	24	3	23	3	64	8	Ras-related protein Rab-35
RAB6B	16	3			50	6	Ras-related protein Rab-6B
RALA	7	4	12	4	51	10	Ras-related protein Ral-A
RALB	5	3	9	3	48	9	Ras-related protein Ral-B
RAP1A					68	11	Ras-related protein Rap-1A
RAP2A					23	6	Ras-related protein Rap-2a
RAP2B	4	3	1	1	29	7	Ras-related protein Rap-2b
RAP2C					40	9	Ras-related protein Rap-2c
RRAS	5	2	2	1	16	5	Ras-related protein R-Ras
RSU1	2	1	7	2	10	5	Ras suppressor protein 1
S100-A14	8	4	2	2	15	8	Protein
SCARB2	3	2	6	3	28	6	Lysosome membrane protein 2

Table S2E cont.

Synonym	SW948-wt cells Prot M	Pept M	-cld7kd cells Prot M	Pept M	SW948-wt TEX Prot M	Pept M	Protein Description
SDCBP					454	16	Syntenin-1
SDCBP2					85	13	Syntenin-2
SERINC5					68	8	Serine incorporator 5 SERINC5
SERPINE2					213	20	Glia-derived nexin SERPINE2
SLC1A5	25	6	23	6	145	13	Neutral amino acid transporter B(0)
SLC29A1					31	7	Equilibrative nucleoside transporter 1
SLC2A1	19	4	6	2	130	8	Solute carrier family 2, facilitated glucose transporter member 1
SLC44A1					100	12	Choline transporter-like protein 1
SLC44A2					60	12	Choline transporter-like protein 2
SMPDL3B					18	7	Acid sphingomyelinase-like phosphodiesterase 3b
SRC	6	3	1	1	14	6	Proto-oncogene tyrosine-protein kinase Src
ST14					39	9	Suppressor of tumorigenicity 14 protein
STOM					32	9	Erythrocyte band 7 integral membrane protein
STX12					9	6	Syntaxin-12
STX3	3	2	1	1	26	9	Syntaxin-3
STX4					14	6	Syntaxin-4
STXBP2			5	4	15	9	Syntaxin-binding protein 2
TGFBI					44	16	Transforming growth factor-beta-induced protein ig-h3
THBS1					13	9	Thrombospondin-1
TMEM106B					15	5	Transmembrane protein 106B
TMPRSS4					23	8	Transmembrane protease serine 4
TNIK	2	1	3	1	31	10	TRAF2 and NCK-interacting protein kinase
TOM1L1					38	11	TOM1-like protein 1
TSG101					82	15	Tumor susceptibility gene 101 protein
TSPAN6					61	6	Tetraspanin-6
TSPAN8	2	1	1	1	85	7	Tetraspanin-8
TTYH3					63	7	Protein tweety homolog 3
TUBA1A					160	16	Tubulin alpha-1A chain
TUBB1					12	5	Tubulin beta-1 chain
UXS1					7	5	UDP-glucuronic acid decarboxylase 1
VPS25	4	2	7	4	22	7	Vacuolar protein-sorting-associated protein 25
VPS28					42	13	Vacuolar protein sorting-associated protein 28 homolog
VPS37B					28	6	Vacuolar protein sorting-associated protein 37B
VPS4B					10	6	Vacuolar protein sorting-associated protein 4B
YES1					14	5	Tyrosine-protein kinase Yes

Table S2F

Higher protein recovery in SW948-wt TEX than SW948-wt cells

	SW948-wt cells		SW948-wt TEX		
**Synonyme.**	**Prot M**	**Pept M**	**Prot M**	**Pept M**	**Protein Description**
ANXA11	3	2	66	17	Annexin A11
ANXA2	15	8	116	23	Annexin A2
ANXA7			10	6	Annexin A7
ASS1			27	9	Argininosuccinate synthase
ATP5L	30	6	133	24	ATP synthase subunit g, mitochondrial
COPE	6	3	19	8	Coatomer subunit epsilon
DHX9	14	6	34	16	ATP-dependent RNA helicase A
DNAJC13			7	5	DnaJ homolog subfamily C member 13
DNPEP	1	1	17	5	Aspartyl aminopeptidase
DPEP1	11	5	96	17	Dipeptidase 1
EIF3F	6	3	19	10	Eukaryotic translation initiation factor 3 subunit F
EIF3F			30	6	Eukaryotic translation initiation factor 3 subunit F
EIF3L	4	2	58	12	Eukaryotic translation initiation factor 3 subunit L
EIF3M	5	3	24	12	Eukaryotic translation initiation factor 3 subunit M
EIF4E	1	1	13	5	Eukaryotic translation initiation factor 4E
FLOT1			64	23	Flotillin-1
FLOT2			52	23	Flotillin-2
GARS			102	20	Glycine--tRNA ligase
HBA1	2	1	133	5	Hemoglobin subunit alpha
HIST1H2AD			62	5	Histone H2A type 1-D
ITGB4	19	9	128	32	Integrin beta-4
NANS	6	3	26	8	Sialic acid synthase
NT5C3A	2	2	108	23	Cytosolic 5'-nucleotidase 3A
PSMB6	6	2	23	7	Proteasome subunit beta type-6
PYGB	6	5	71	21	Glycogen phosphorylase, brain form
PYGL			26	7	Glycogen phosphorylase, liver form
RAB22A			20	6	Ras-related protein Rab-22A
RAB5A	8	4	36	8	Ras-related protein Rab-5A
RACK1	12	7	163	18	Receptor of activated protein C kinase 1
RANGAP1	2	2	82	10	Ran GTPase-activating protein 1
RPL10A	2	1	34	7	60S ribosomal protein L10a
RPL12	5	3	28	6	60S ribosomal protein L12
RPL18	1	1	33	7	60S ribosomal protein L18
RPL6			22	10	60S ribosomal protein L6
RPLP0	6	4	139	13	60S acidic ribosomal protein P0
RPLP2			23	5	60S acidic ribosomal protein P2
RPS7	1	1	15	5	40S ribosomal protein S7
RPSA	3	3	159	11	40S ribosomal protein SA
SEPT9	9	3	20	8	Septin-9
SLC7A1	1	1	25	5	High affinity cationic amino acid transporter 1
SYNGR2	4	2	31	5	Synaptogyrin-2
WARS	3	2	34	7	Tryptophan--tRNA ligase, cytoplasmic

Table S2G

Higher protein recovery in SW948-wt TEX than SW948-cld7kd cells

	SW948-cld7kd cells		SW948-wt TEX		
**Synonym**	**Prot M**	**Pept M.**	**Prot M**	**Pept M.**	**Protein Description**
ACTR1A			11	5	Alpha-centractin
CDH1	6	3	21	8	Cadherin-1
CTNNB1	4	3	70	20	Catenin beta-1
FTH1	11	4	102	11	Ferritin heavy chain
IGF2R	30	12	42	25	Cation-independent mannose-6-phosphate recept.
KRAS			15	5	GTPase KRas
LAMTOR1	7	3	15	6	Ragulator complex protein LAMTOR1
LIN7C			19	9	Protein lin-7 homolog C
OLFM4			8	6	Olfactomedin-4
PRMT1	18	4	59	11	Protein arginine N-methyltransferase 1
PSMD7	10	5	38	11	26S proteasome non-ATPase regulatory subunit 7
PTPRF			20	13	Receptor-type tyrosine-protein phosphatase F
PYCRL	2	1	12	5	Pyrroline-5-carboxylate reductase 3
RAB25			12	5	Ras-related protein Rab-25
RAP1B	16	6	73	12	Ras-related protein Rap-1b
RDX			82	10	Radixin
REEP6	3	2	8	5	Receptor expression-enhancing protein 6
RHOB			16	5	Rho-related GTP-binding protein RhoB
SLC16A3			36	5	Monocarboxylate transporter 4
SLC3A2	45	12	169	24	4F2 cell-surface antigen heavy chain
TM9SF2	7	3	43	10	Transmembrane 9 superfamily member 2
TSPAN14			61	9	Tetraspanin-14
TSPAN15			38	6	Tetraspanin-15
TSTA3	3	2	18	5	GDP-L-fucose synthase

Table S3

Claudin protein complexes in cells and TEX

Table S3A

Association with palmitoylated and non-palmitoylated cld7 in cells

	cld7 IP				
	ASML-wt cells		-cld7mP cells		
**synonym**	**Prot.M**	**Pept.M**	**Prot.M**	**Pept.M**	**Protein Description**
Bcap31	11	5	16	9	B-cell receptor-associated protein 31
Cabp1	10	8	10	10	calcium binding protein
Cisd2	2	2	6	4	CDGSH iron-sulfur domain-cont. protein 2
Copa	127	83	164	127	coatomer protein complex subunit alpha
Ddx5	18	15	8	7	DEAD box helicase 5
Eef1a1	93	48	61	55	elongation factor 1-alpha, beta, delta, gamma
Erp29	6	4	5	3	endoplasmic reticulum resident protein 29
Ewsr1	13	11	29	21	RNA-binding protein EWS
Fkbp12	4	2	5	4	FK506-binding protein 12
Hspa1a	23	18	16	11	heat shock protein 70
Hspe1	11	4	13	8	chaperonin 10
Myl3	29	17	43	30	myosin light chain 3
Myo1b	51	41	93	75	myosin I heavy chain
Rab2a	12	9	14	11	Rab-2A
Rpl10	9	6	7	5	60S ribosomal protein L10
Rpl17	5	3	6	6	60S ribosomal protein L17
Rplp0	20	16	19	16	60S acidic ribosomal protein P0
Rps17	13	4	8	5	40S ribosomal protein S17
Rps2	19	10	11	7	ribosomal protein S2
Rps21	7	4	12	9	40S ribosomal protein S21
Rps26	3	3	5	4	40S ribosomal protein S26
Rps3a	22	11	22	10	40S ribosomal protein S3a
Rps9	12	7	8	5	Ribosomal protein L9
Rpsa	16	12	15	12	40S ribosomal protein SA
Sp120	22	12	16	10	serine protease 120
Sqrdl	23	17	20	17	sulfide:quinone oxidoreductase
Testin	129	74	106	66	testin
Ubc	35	12	36	14	ubiquitin C

Table S3B

Association with palmitoylation-competent cld7 in cells

	cld7 IP				
	ASML-wt cells		-cld7mP cells		
**synonym**	**Prot.M**	**Pept.M**	**Prot.M**	**Pept.M**	**Protein Description**
Aifm1	8	4	1	1	apoptosis-inducing factor
Cse1	25	16			exportin-2
Dnajp9	38	22	3	2	dnaJ homolog subfamily C member 9
Hnrnpm	24	19			heterogeneous nuclear ribonucleoprotein M
Hnrpd	9	8	3	2	RNA binding protein p42 AUF1
Hspa1b	124	101			heat shock protein 70kd 1B
Hspa9	14	11	6	4	heat shock protein 90, alpha
Idh3a	8	5			isocitrate dehydrogenase subunit alpha
Il33	10	7			interleukin-33
Impdh2	34	23			inosine-5'-monophosphate dehydrogenase 2
Impdh2	34	23	2	1	inosine-5'-monophosphate dehydrogenase 2
Ionp1	8	6			lon protease homolog
Lmo2	26	16			LIM domain 2
Myh13	16	12			myosin-13
Parp1	19	13			poly [ADP-ribose] polymerase 1
Rpl4	13	9	4	2	60S ribosomal protein L4
Smarca5	7	5			SWI/SNF-related matrix-associated actin-dep. regulator of chromatin A5
Ssbp1	16	12			single-stranded DNA-binding protein, mitochondrial precursor
Xrcc5	5	4			X-ray repair cross-complementing protein 5
Xrcc6	8	5			X-ray repair complementing defective repair

Table S3C

Association with palmitoylation-deficient cld7 in cells

	cld7 IP				
	ASML-wt cells		-cld7mP cells		
**synonym**	**Prot.M**	**Pept.M**	**Prot.M**	**Pept.M**	**Protein Description**
Ahnak	4	1	56	32	neuroblast differentiation-associated protein AHNAK
Ahsg	2	1	30	23	alpha-2-HS-glycoprotein
Far1			12	9	fatty acyl-CoA reductase 1
Flii	4	2	14	9	flightless-1 homolog
Gnai3	15	14	30	29	guanine nucleotide-binding protein G(k) subunit alpha
Hadhb			10	6	hydroxyacyl-CoA dehydrogenase, beta subunit
Hsd17b12			4	4	estradiol 17-beta-dehydrogenase 12
Hsph1			8	6	heat shock protein 105 kDa
Krt33b			11	8	keratin, type I cuticular Ha3-II
Krt4			36	26	keratin, type II cytoskeletal 4
Lad1			11	7	ladinin 1
Ldhal6b			5	4	L-lactate dehydrogenase A-like 6B
Myl2			137	114	myosin light chain 2
Myl6l			204	163	myosin light polypeptide 6
Ppa1			4	4	pyrophosphatase 1
Prkcz	14	6	20	15	protein kinase C zeta
Rab18	6	1	18	14	ras-related protein Rab-18
Rab5a			8	6	ras-related protein Rab-5A
Reep6	2	1	6	6	receptor expression-enhancing protein 6
Tecr	1	1	12	5	very-long-chain enoyl-CoA reductase
Tfg	12	7	51	26	Trk-fused gene
Trim21	3	2	64	53	E3 ubiquitin-protein ligase TRIM21

Table S3D

Co-immunoprecipitation with palmitoylated and non-palmitoylated cld7 in TEX

	anti-cld7 IP				
	ASML-wt TEX		-cld7mP TEX		
**Synonym**	**Prot.M**	**Pept.M**	**Prot.M**	**Pept.M**	**Protein Description**
Acot7	25	4	40	6	Acyl-CoA thioesterase 7
Actn4	34	14	27	15	alpha-actinin 4
Actr2	21	6	55	9	Actin-related protein 2
Ahcy	44	10	67	10	Adenosylhomocysteinase
Akr1b1	37	7	60	8	Aldose reductase
Akr1b8	47	10	144	12	aldose reductase-related protein 1
Alb	139	4	187	5	Serum albumin
Aldoa	32	12	25	9	Fructose-bisphosphate aldolase A
Anxa2	113	17	91	15	annexin A2
Anxa5	42	12	45	14	Annexin A5
Ap2s1	5	3	12	4	AP-2 complex subunit sigma
Aprt	16	6	58	8	Adenine phosphoribosyltransferase
Asap1	7	2	8	5	Arf-GAP with SH3 domain, ANK repeat and PH domain-cont. protein 1
Atp5b	27	11	58	16	ATP synthase subunit beta
Atp6ap2	5	3	7	5	Renin receptor
C3	10	4	35	8	Complement C3
Cand1	12	7	6	4	Cullin-associated NEDD8-dissociated protein 1
Cd81	12	3	33	4	CD81 tetraspanin
Cd9	13	4	39	5	CD9 tetraspanin
Cltc	35	18	81	26	Clathrin heavy chain
Dstn	17	3	36	4	Destrin
Eno3	69	5	58	6	Enolase beta
Epcam	10	5	17	5	Epithelial cell adhesion molecule
Ezr	50	8	117	14	Ezrin
Fn1	374	76	211	62	Fibronectin
G6pc	13	6	42	8	Glucose-6-phosphatase
Gapdh	98	11	158	12	Glyceraldehyde-3-phosphate dehydrogenase
Got2	64	9	60	14	Aspartate aminotransferase
Gsn	4	3	16	4	Gelsolin
Hsp90aa1	218	25	514	35	Heat shock protein HSP 90-alpha
Hsp90b1	23	3	50	5	Endoplasmin
Hspa2	110	9	180	10	Heat shock-related 70 kDa protein 2
Hspa5	33	8	57	14	78 kDa glucose-regulated protein
Htra1	103	20	114	27	Serine protease HTRA1
Jup	5	4	5	3	Junction plakoglobin
Kb15	53	9	55	5	keratin, type II cytoskeletal cochleal
Kpnb1	11	2	19	4	Importin subunit beta-1
Krt1	50	8	92	7	Keratin, type II cytoskeletal 1
Krt10	49	10	66	10	Keratin, type I cytoskeletal 10
Krt14	25	7	39	10	Keratin, type I cytoskeletal 14
Krt16	16	4	26	8	keratin, type I cytoskeletal 16
Krt17	41	11	32	13	keratin, type I cytoskeletal 17
Krt18	32	10	20	8	keratin, type I cytoskeletal 18
Krt19	42	15	41	13	keratin, type I cytoskeletal 19
Krt42	16	6	30	11	Keratin, type I cytoskeletal 42
Krt5	42	8	106	11	Keratin, type II cytoskeletal 5
Krt75	35	9	46	6	Keratin, type II cytoskeletal 75
Ldha	163	20	185	18	L-lactate dehydrogenase
Mfge8	45	15	127	21	Lactadherin
Msn	48	8	78	10	Moesin
Myh10	19	10	25	14	Myosin, heavy polypeptide 10
Myh9	36	15	41	16	Myosin, heavy polypeptide 9
Myof	10	3	16	5	Myoferlin
Pfn1	61	7	115	6	Profilin-1
Pgk1	150	17	164	16	Phosphoglycerate kinase 1
Phb2	14	9	10	6	Prohibitin-2
Pkm	247	22	457	32	Pyruvate kinase PKM
Ppia	26	9	31	10	Peptidyl-prolyl cis-trans isomerase A
Prdx2	18	4	22	4	Peroxiredoxin-2
Psma1	20	9	36	8	Proteasome subunit alpha type-1
Psma2	12	6	23	7	Proteasome subunit alpha type-2

Table S3D cont.

	anti-cld7 IP				
	ASML-wt TEX		-cld7mP TEX		
**Synonym**	**Prot.M**	**Pept.M**	**Prot.M**	**Pept.M**	**Protein Description**
Psma3	6	4	23	7	Proteasome subunit alpha type
Psma6	13	6	45	10	Proteasome subunit alpha type-6
Psmb3	26	6	41	6	Proteasome subunit beta type
Psmb7	3	2	11	4	Proteasome subunit beta type-7
Psmb8	10	6	70	11	Proteasome subunit beta type
Psmb9	10	3	12	4	Proteasome subunit beta type
Rab10	27	5	20	3	Ras-related protein Rab-10
Rab11b	12	8	43	10	Ras-related Rab-11B
Rheb	6	3	7	4	GTP-binding protein Rheb
Rpl10a	11	5	29	5	60S ribosomal protein L10a
Sept2	21	5	46	7	Septin-2
Serpinc1	6	4	16	3	Serpin family C member 1
Sptan1	9	6	19	9	Spectrin alpha chain 1
Tkt	24	8	44	11	Transketolase
Tpi1	21	7	34	7	Triosephosphate isomerase 1
Tspan8	14	3	29	5	Tetraspanin 8
Vcp	91	25	134	21	Transitional endoplasmic reticulum ATPase
Vdac1	29	9	23	8	Voltage-dependent anion-selective channel protein 1
Xpo1	7	3	15	6	exportin 1
Ywhag	27	5	25	3	14-3-3 protein gamma

Table S3E

Co-immunoprecipitation with palmitoylation competent cld7 in TEX

	anti-cld7 IP				
	ASML-wt TEX		-cld7mP TEX		
**Synonym**	**Prot.M**	**Pept.M**	**Prot.M**	**Pept.M**	**Protein Description**
Actn1	16	6			alpha-actinin 1
Col18a1	60	15	6	3	Procollagen, type XVIII, alpha 1
Ddost	8	5			Dolichyl-diphosphooligosaccharide--protein glycosyltransferase
Fasn	12	6			Fatty acid synthase
H2afy	122	13	13	5	Core histone macro-H2A.1
Hist1h1a	93	9			Histone H1.1
Hp1bp3	41	9	2	1	Heterochromatin protein 1-binding protein 3
Itga3	17	8	7	4	Integrin, alpha 3
Itga6	17	8	6	4	Integrin, alpha 6
Itgb1	16	10	4	4	Integrin beta 1
Ncl	127	24			Nucleolin
Npm1	69	7	3	2	Nucleophosmin
Pdia4	8	4			disulfide-isomerase A4
Set	34	5	4	2	SET nuclear protooncogene
Taldo1	7	4			Transaldolase 1

Table S3F

Co-immunoprecipitation with non-palmitoylated cld7

	anti-cld7 IP				
	ASML-wt TEX		-cld7mP TEX		
**Synonym**	**Prot.M**	**Pept.M**	**Prot.M**	**Pept.M**	**Protein Description**
Anxa3	3	1	18	7	annexin A3
Apob			15	6	Apolipoprotein B-100
Arpc1b			10	4	Actin-related protein 2/3 complex subunit 1B
Arpc3	4	1	16	7	Actin-related protein 2/3 complex subunit 3
Asl			84	14	Argininosuccinate lyase
Cad			12	4	DNA fragmentation factor subunit beta
Capns1	2	1	10	4	Calpain small subunit 1
Cav2			27	4	Caveolin2
Cct2	12	6	147	19	T-complex protein 1 subunit beta
Cct3	9	4	74	16	T-complex protein 1 subunit gamma
Cct4	7	3	82	14	T-complex protein 1 subunit delta
Cct5	1	1	40	11	T-complex protein 1 subunit epsilon
Cct6a	14	4	77	14	Chaperonin containing Tcp1, subunit 6A
Cct7	2	2	65	15	T-complex protein 1 subunit eta
Cct8	8	5	64	14	Chaperonin subunit theta
Cfl1	1	1	28	6	Cofilin-1
Clic1	3	2	33	7	Chloride intracellular channel protein 1
Ddx39b			13	7	Spliceosome RNA helicase Ddx39b
Eef1d	4	1	30	5	Elongation factor 1-delta
Eif2s1			19	4	Eukaryotic translation initiation factor 2 subunit 1
Eif2s3			16	4	Eukaryotic translation initiation factor 2 subunit 3
Eif3b	6	3	38	8	Eukaryotic translation initiation factor 3 subunit B
Eif3c	1	1	50	13	Eukaryotic translation initiation factor 3 subunit C
Eif3d			12	5	Eukaryotic translation initiation factor 3 subunit D
Eif3f	6	2	28	7	eukaryotic translation initiation factor 3 subunit F
Eif3h	1	1	44	11	Eukaryotic translation initiation factor 3 subunit H
Eif3i			12	5	Eukaryotic translation initiation factor 3 subunit I
Eif3m	8	5	107	12	Eukaryotic translation initiation factor 3 subunit M
Eprs	3	2	33	7	glutamyl-propyl-tRNA synthetase
Farsa	1	1	26	9	Phenylalanine--tRNA ligase alpha subunit
Hspa4	4	3	71	16	Heat shock 70 kDa protein 4
Kars			19	6	Lysine--tRNA ligase
Lgals3	1	1	7	4	Galectin-3
LOC286987			6	4	Hemiferrin
Mdh2	13	3	41	9	Malate dehydrogenase 2
Mrto4			10	4	MRT4 homolog, ribosome maturation factor
Mtap	2	2	21	6	Methythioadenosine phosphorylase
Myh14			14	5	Myosin heavy chain 14
Nemf	3	2	17	5	Nuclear export mediator factor
Pa2g4	5	3	45	9	Proliferation-associated protein 2G4
Pcna	3	2	38	9	Proliferating cell nuclear antigen
Pdcd6	2	1	12	4	Programmed cell death 6
Pdcd6ip	4	2	8	6	Programmed cell death 6-interacting protein
Plscr3			22	6	Phospholipid scramblase 3
Psma4	12	1	40	8	Proteasome subunit alpha type
Psmd13	2	1	16	6	26S proteasome non-ATPase regulatory subunit 13
Psmd3			50	11	Proteasome 26S subunit, non-ATPase, 3
Ptgfrn			38	15	Prostaglandin F2 receptor negative regulator
Ptrf			18	6	Polymerase I and transcript release factor
Rab14	9	3	45	11	Rab14, member RAS oncogene family
Rab5c	5	2	16	7	Rab5c, member RAS oncogene family
Rtn4			14	6	Reticulon 4
Sdcbp	17	3	44	6	Syntenin-1
Sept7			24	4	Septin-7
Shmt1	5	2	79	12	Serine hydroxymethyltransferase 1
Ssr4	3	2	19	6	Translocon-associated protein subunit delta
Tagln2	1	1	105	27	Transgelin-2
Tcp1	19	4	141	20	T-complex protein 1 subunit alpha
Thbs1	2	1	11	5	Thrombospondin 1
Tsn			12	4	translin
Vat1	1	1	24	5	Synaptic vesicle membrane protein VAT-1 homolog

Table S4

Cld7-dependent miRNA recovery in cells and TEX

Table S4A

Cld7 dependent miRNA recovery in cells

name	ASML-wt cells	-cld7kd cells[1]	-cld7mP cells[1]
**miR-34a-5p**	1337	**436**	**538**
**miR-141-3p**	6627	**1705**	4159
**miR-10a-5p**	2420	2162	**339**
**miR-26b-5p**	1287	1098	**653**
**miR-1224**	1031	**3537**	**2681**
**let-7e-5p**	1110	**2615**	1433
**miR-103-3p**	1734	**3691**	2674
**miR-301a-3p**	684	**1378**	1062

Table S4B

Cld7 dependent miRNA recovery in TEX

Name	ASML-wt TEX	-cld7kd TEX[a]	-cld7mP TEX[a]
**let-7b-5p**	3940	**1840**	**1628**
**let-7c-5p**	4653	**1655**	**1660**
**miR-125b-5p**	17652	**5483**	**2713**
**miR-200b-3p**	1365	**280**	**350**
**miR-347**	1236	**224**	**431**
**miR-494-3p**	1776	**180**	**385**
**let-7a-5p**	3250	**567**	1903
**let-7d-5p**	1264	**239**	1117
**let-7f-5p**	2183	**167**	1464
**let-7g-5p**	1377	**454**	979
**miR-10a-5p**	1185	**309**	673
**miR-21-5p**	8288	**3221**	5094
**miR-29b-3p**	2010	**586**	1163
**miR-16-5p**	2488	1625	**1035**
**miR-183-5p**	1096	698	**529**
**miR-1224**	10744	**25080**	**23754**
**miR-211-3p**	363	**2152**	**5280**
**miR-3584-5p**	281	**1589**	**832**
**miR-483-5p**	472	**1472**	**1655**
**miR-6216**	190	**684**	**1124**
**miR-3084c-5p**	210	**1473**	283
**miR-222-3p**	185	**1188**	234
**miR-3473**	1795	**5160**	2700
**miR-130a-3p**	749	**1683**	836
**miR-188-5p**	538	**1215**	502
**miR-210-3p**	217	**4005**	247
**miR-221-3p**	338	**1200**	360
**miR-155-5p**	401	355	**1977**
**miR-1896**	448	753	**5895**
**miR-290**	214	337	**1622**
**miR-3099**	166	180	**1034**

[1] >2-fold differences: bold and underlined

Table S4C

miR recovery in SW948-wt and -cld7kd cells and TEX

Name[2]	wt>cld7kd cells[1] SW948-wt cells	-cld7kd cells	wt>cld7kd TEX[1] SW948-wt TEX	-cld7kd TEX
let-7e-5p	927	471	1307	497
let-7i-5p			3208	1449
miR-10a-5p	9583	5304	14495	5343
miR-1225-5p	1197	585		
miR-1246	3783	1176	94418	44135
miR-1273g-3p	4890	1992		
miR-1290	(597)	(181)	47002	14923
miR-135b-5p	1615	689	1211	569
miR-151b			1170	347
miR-210-3p	7220	548	2916	1074
miR-22-3p	1870	495	2267	520
miR-23a-3p	2504	1072	4279	2006
miR-27a-3p	3703	1408		
miR-29b-3p	30513	14033		
miR-31-5p	1037	444	1477	359
miR-320d			7963	3625
miR-320e			6077	1611
miR-3934-5p	3048	102	2748	660
miR-4299	2287	1316	3193	933
miR-4530	4727	1976		
miR-4455			2632	317
miR-451a			4534	1407
miR-4763-3p	(530)	(188)	3043	1207
miR-574-5p	1011	482	17605	1774
miR-6753-5p			1560	657
miR-8089	4219	104	3934	870

	wt<cld7kd cells[1] SW948-wt cells	-cld7kd cells	wt<cld7kd TEX[1] SW948-wt TEX	-cld7kd TEX
miR-1260a			974	3131
miR-1260b			920	2038
miR-1287-5p			285	4315
miR-146a-5p			871	1883
miR-27b-3p	1031	2366		
miR-3141			301	2311
miR-3162-5p	2125	4573	5165	10986
miR-3665			1728	4103
miR-3937			384	1685
miR-4271			702	4049
miR-4298			446	2510
miR-4430			621	1632
miR-4443			1280	3960
miR-4485-5p			739	2335
miR-4487			199	1952
miR-4497			410	1566
miR-4685-5p			371	5537
miR-483-5p			506	2323
miR-6087	3416	5624	14591	38655
miR-6089	7536	14649	17444	42264
miR-6124			1037	2416
miR-642a-3p	1312	3214		
miR-6786-5p			742	1504
miR-6800-5p			2673	6057
miR-6869-5p	1790	3385	6037	14361
miR-7108-5p			818	1948
miR-7-5p	1109	4222	612	1995

[1] >2-fold difference: bold

[2] underlined: miRNA consistently regulated in SW948-cld7kd cells and TEX

Table S4D

miR recovered at high level in ASML-wt TEX compared to -cld7kd cells

Name	ASML-wt TEX	-cld7kd cells[1]	Name	ASML-wt TEX	-cld7kd cells[1]
**miR-125b-5p**	46218	6457	**miR-671**	537	34
**miR-1224**	28131	3537	**miR-3102**	528	33
**miR-466b-5p**	15829	206	**miR-330-3p**	514	36
**miR-23a-3p**	7314	2217	**miR-451-5p**	504	36
**miR-547-5p**	7072	34	**miR-6216**	497	208
**miR-3473**	4701	449	**miR-30d-5p**	488	100
**miR-494-3p**	4650	255	**miR-99b-5p**	452	202
**miR-652-5p**	3823	1478	**miR-206-3p**	407	44
**miR-347**	3236	105	**miR-1249**	403	44
**miR-183-5p**	2870	1041	**miR-196c-3p**	397	82
**miR-200c-3p**	2374	120	**miR-551b-5p**	394	55
**miR-381-3p**	2123	35	**miR-3573-3p**	382	49
**miR-212-3p**	2119	255	**miR-328a-3p**	365	44
**miR-324-3p**	2008	675	**miR-1839-3p**	340	66
**miR-672-5p**	1633	164	**miR-3562**	335	87
**miR-188-5p**	1409	136	**miR-134-5p**	334	81
**miR-32-3p**	1349	46	**miR-3064-5p**	320	32
**miR-483-5p**	1236	120	**miR-760-5p**	320	33
**miR-1896**	1174	90	**miR-423-5p**	311	61
**miR-300-3p**	970	36	**miR-770-3p**	309	54
**miR-211-3p**	949	207	**miR-328a-5p**	309	74
**miR-1306-3p**	866	72	**miR-3564**	307	44
**miR-466d**	840	36	**miR-139-3p**	293	43
**miR-327**	785	41	**miR-487b-3p**	282	47
**miR-30c-5p**	764	260	**miR-296-5p**	273	44
**miR-3584-5p**	737	315	**miR-598-3p**	272	36
**miR-365-3p**	729	179	**miR-331-3p**	267	81
**miR-465-5p**	697	34	**miR-3588**	262	37
**miR-466c-5p**	659	62	**miR-539-5p**	259	36
**miR-125a-3p**	611	95	**miR-202-3p**	252	85
**miR-150-3p**	599	114	**miR-297**	251	33
**miR-320-3p**	587	74	**miR-3085**	246	41
**miR-3593-3p**	587	117	**miR-34b-5p**	241	78
**miR-762**	570	137	**miR-149-3p**	232	56

[1] miRNA with a signal strength >1000 and an >2-fold difference are underlined

Table S4E

miR recovered at high level in SW948-wt TEX compared to -cld7kd cells

Name	SW948-wt TEX	-cld7kd cells[1]	Name	SW948-wt TEX	-cld7kd cells[1]
miR-1290	47002	181	miR-221-3p	528	50
miR-6893-5p	1021	9	miR-331-3p	525	**50**
miR-451a	4534	50	miR-328-5p	516	**50**
miR-1246	94418	1176	miR-4655-5p	508	50
miR-8089	3934	50	miR-1225-5p	5940	585
miR-3934-5p	2748	**50**	miR-6850-5p	504	50
miR-4455	2632	50	miR-4739	1903	190
miR-22-3p	2267	50	miR-584-5p	388	39
miR-630	2093	48	miR-3679-5p	1324	152
miR-6085	2146	50	miR-4430	621	72
miR-574-5p	17605	482	miR-6891-5p	2456	293
miR-6753-5p	1560	50	miR-4298	446	56
miR-31-5p	1477	50	miR-1268a	1735	218
miR-151a-5p	1450	50	miR-4466	2114	266
miR-6076	1412	50	miR-6090	15292	1967
miR-7107-5p	3301	121	miR-188-5p	942	142
miR-5703	1340	50	miR-4459	38879	5944
let-7e-5p	1307	**50**	miR-4271	702	111
miR-4270	2723	107	miR-6803-5p	1071	183
miR-151b	1170	50	miR-2861	2110	376
miR-642b-3p	1085	50	miR-6812-5p	480	90
miR-30d-5p	982	50	miR-210-3p	2916	548
miR-575	964	50	miR-320c	5777	1180
miR-4534	451	24	miR-1287-5p	285	60
miR-135b-5p	911	**50**	miR-6879-5p	3696	816
miR-4788	879	50	miR-320e	6077	1345
miR-6740-5p	870	50	miR-4505	1413	322
miR-30e-5p	861	50	miR-1229-5p	1462	337
miR-374b-5p	829	**50**	miR-320d	7963	1837
miR-7150	5748	350	miR-1275	1096	254
miR-4763-3p	3043	188	miR-6821-5p	4020	957
miR-6728-5p	803	50	miR-6724-5p	745	182
miR-6510-5p	801	50	miR-23a-3p	4279	1072
miR-183-5p	800	**50**	miR-6756-5p	476	122
miR-134-5p	791	**50**	miR-4516	11330	2897
miR-5787	7396	493	miR-197-5p	2291	587
miR-6786-5p	742	50	miR-638	2397	619
miR-4281	12709	854	miR-4741	859	232
miR-3135b	726	50	miR-4442	933	255
miR-1207-5p	4893	344	miR-4530	7089	1976
miR-8072	704	50	miR-3960	17064	4806
miR-4507	672	50	miR-4443	1280	375
miR-222-3p	667	50	miR-7704	1934	656
miR-4685-5p	371	29	miR-6769b-5p	757	274
miR-6829-5p	3342	260	miR-8069	19048	6909
miR-6088	5617	439	miR-10a-5p	14495	5304
miR-4687-3p	4650	368	miR-1202	5255	1986
miR-6794-5p	624	50	miR-6125	3998	1520
miR-6752-5p	619	50	miR-6087	14591	5624
miR-3195	605	50	miR-200a-3p	5483	2125
miR-4306	604	50	miR-3665	1728	711
miR-150-3p	602	**50**	miR-4299	3193	1316
miR-7110-5p	2858	238	miR-6800-5p	2673	1158
miR-6820-5p	600	50	miR-3196	842	369
miR-2392	578	50	miR-320b	4092	1837
miR-6727-5p	575	50	miR-642a-3p	6723	3214
miR-3656	1796	158	miR-6826-5p	6563	3192
miR-203a-3p	555	50	miR-7108-5p	818	399
miR-30c-5p	554	50	miR-1915-3p	1863	923

[1] miRNA with a signal strength >1000 and an >2-fold difference are underlined

Table S5

Reagents

Table S5A

Antibodies

Antibody	origin	supplier
Actin	mouse	Becton Dickinson, HD, G
hCD104	rabbit	Becton Dickinson, HD, G
rCD104 (B5.5)	mouse	(1)[1]
hCD133	rabbit	Becton Dickinson, HD, G
hCD166	mouse	Becton Dickinson, HD, G
rCD166	mouse	Becton Dickinson, HD, G
hCD29	mouse	Becton Dickinson, HD, G
rCD29	mouse	Becton Dickinson, HD, G
hCD44v6 (vFF18)	mouse	(2)
rCD44v6 (A2.6)	mouse	(1)
hCD49c	mouse	Becton Dickinson, HD, G
hCD49f	mouse	Becton Dickinson, HD, G
rCD49f	mouse	Becton Dickinson, HD, G
hCD9	mouse	Becton Dickinson, HD, G
rCD9 (B2C11)	mouse	ATCC[2]
cld7	guinea pig	(3)
hEGFR	mouse	BioTrend, Cologne, G
hEpC (HEA125)	mouse	(4)
rEpCAM (D5.7)	mouse	(1)
ezrin	rabbit	Becton Dickinson, HD, G
JUP	rabbit	Abcam, Cambridge, England
hJAM	mouse	Becton Dickinson, HD, G
MFGE8	rabbit	Abcam, Cambridge, England
K-ras	mouse	SantaCruz, HD, G
Src	rabbit	Santa Cruz, HD, G
hTspan8 (CO029)	mouse	(5)
rTspan8 (D6.1)	mouse	(1)
dye- or biotin-labeled secondary antibodies / Streptavidin		Dianova, Becton Dickinson, Amersham

[1] References

1. Matzku, S., Wenzel, A., Liu, S., et al. (1989). Antigenic differences between metastatic and nonmetastatic BSp73 rat tumor variants characterized by monoclonal antibodies. Cancer Res. 49, 1294-1299.
2. Seiter, S., Schadendorf, D., Herrmann, K., et al. (1996). Expression of CD44 variant isoforms in malignant melanoma. Clin. Cancer Res. 2, 447-456.
3. Ladwein, M., Pape, U.F., Schmidt, D.S., et al. (2005). The cell-cell adhesion molecule EpCAM interacts directly with the tight junction protein claudin-7. Exp. Cell Res. 309, 345-357.
4. Momburg, F., Moldenhauer, G., Hämmerling, G.J., et al. (1987). Immunohistochemical study of the expression of a Mr 34,000 human epithelium-specific surface glycoprotein in normal and malignant tissues. Cancer Res. 47, 2883-2891.
5. Sela, B.A., Steplewski, Z., and Koprowski, H. (1989). Colon carcinoma-associated glycoproteins recognized by monoclonal antibodies CO-029 and GA22-2. Hybridoma 8, 481-491.

[2] ATCC: American Type Cell Culture Collection

Table S5B
Primers

rno-let-7a-5p SL	GTTGGCTCTGGTGCAGGGTCCGAGGTATTCGCACCAGAGCCAAC AACTAT
rno-let-7a-5p fw	TGTGTTGTGAGGTAGTAGGTTGT
rno-miR-125b-5p SL	GTTGGCTCTGGTGCAGGGTCCGAGGTATTCGCACCAGAGCCAAC TCACAA
rno-miR-125b-5p fw	TGTGTTCCCTGAGACCCTAAC
rno-miR-347 SL	GTTGGCTCTGGTGCAGGGTCCGAGGTATTCGCACCAGAGCCAAC TGGGCG
rno-miR-347 fw	GTGTGTGTCCCTCTGGGT
rno-mir-125b-5p SL	GTTGGCTCTGGTGCAGGGTCCGAGGTATTCGCACCAGAGCCAAC TCACAA
rno-mir-125b-5p fw	TGTGTTCCCTGAGACCCTAAC
rno-miR-21-5p SL	GTTGGCTCTGGTGCAGGGTCCGAGGTATTCGCACCAGAGCCAAC TCAACA
rno -miR-21-5p fw	TGGTTTGGTAGCTTATCAGACTGA
rno-miR-16-5p SL	GTTGGCTCTGGTGCAGGGTCCGAGGTATTCGCACCAGAGCCAAC CGCCAA
rno-miR-16-5p fw	GTTTGGTAGCAGCACGTAAATA
rno-mir-1224 SL	GTTGGCTCTGGTGCAGGGTCCGAGGTATTCGCACCAGAGCCAAC CTCCAC
rno-mir-1224 fw	TTGGTGAGGACTGGGGAG
rno-miR-24-3p SL	GTTGGCTCTGGTGCAGGGTCCGAGGTATTCGCACCAGAGCCAAC CTGTTC
rno-miR-24-3p fw	GGTGTGGCTCAGTTCAGC
rno-miR-652-5p SL	ACAACCCUAGGAGGGGGUGCCAU
rno-miR-652-5p fw	GGTGACAACCCTAGGAGGG
rno-miR-1224 fw	CTATGTGAGGACTGGGGAGG
rno-miR-1224 SL	GTTGGCTCTGGTGCAGGGTCCGAGGTATTCGCACCAGAGCCAACCTCCAC
rno-mir-466b-5p SL	GTTGGCTCTGGTGCAGGGTCCGAGGTATTCGCACCAGAGCCAAC CATGGA
rno-mir-466b-5p fw	GTTTGGTATGTGTGTGTGTATG
rno-mir-324-3p SL	GTTGGCTCTGGTGCAGGGTCCGAGGTATTCGCACCAGAGCCAAC CCAGCA
rno-mir-324-3p fw	TTGTCCACTGCCCCAGGT
rno-mir-547-5p SL	GTTGGCTCTGGTGCAGGGTCCGAGGTATTCGCACCAGAGCCAAC TGGGTG
rno-mir-547-5p fw	GGGTCACTTCAGGATGTAC
hsa-mir-1246 SL	GTTGGCTCTGGTGCAGGGTCCGAGGTATTCGCACCAGAGCCAAC CCTGCT
hsa-mir-1246 fw	GGGGGGGAATGGATTTTTGG
hsa-mir-1290 SL	GTTGGCTCTGGTGCAGGGTCCGAGGTATTCGCACCAGAGCCAAC TCCCTG
hsa-mir-1290 fw	GGGGGGTGGATTTTTGGAT
hsa-mir-451a SL	GTTGGCTCTGGTGCAGGGTCCGAGGTATTCGCACCAGAGCCAAC AACTCA
hsa-mir-451a fw	GTTTGGAAACCGTTACCATTAC
hsa-mir-4270 SL	GTTGGCTCTGGTGCAGGGTCCGAGGTATTCGCACCAGAGCCAAC GCCCTC
hsa-mir-4270 fw	TTGGTCAGGGAGTCAGGG
hsa-mir-574-5p SL	GTTGGCTCTGGTGCAGGGTCCGAGGTATTCGCACCAGAGCCAAC ACACAC
hsa-mir-574-5p fw	GGTGAGTGTGTGTGTGTGA
Universal reverse primer	GTGCAGGGTCCGAGGT

Table S6
Full names of synonyms

**Synonym**	**Full name**
A0A0G2K0V8	Nuclear migration protein nudC like
A2M	Alpha-2-macroglobulin
AARS	Alanine--tRNA ligase
Abcb7	ATP-binding cassette sub-family B member 7
ABCC3	Canalicular multispecific organic anion transporter 2
ABCE1	ATP-binding cassette sub-family E member 1
ABCE1 / D3ZD23	ATP binding cassette subfamily E member 1
ACE	Angiotensin-converting enzyme
ACO2	Aconitate hydratase, mitochondrial
ACOT7	Acyl-CoA thioesterase 7
ACOT8	Acyl-CoA thioesterase 8
ACP2	Lysosomal acid phosphatase
ACSL3	Long-chain-fatty-acid--CoA ligase 3
ACTB	Actin, cytoplasmic
ACTBL2	Actin, beta-like 2
ACTC1	Actin, alpha cardiac muscle 1
ACTL6A	Actin-like protein 6A
ACTN1	alpha-actinin-1
ACTN2	Alpha-actinin-2
ACTN4	Alpha-actinin 4
ACTR1A	ARP1 actin related protein 1 homolog A
ACTR1B	ARP1 actin related protein 1 homolog B
ACTR2	Actin-related protein 2
ADA	Adenosine deaminase
ADAM10	Disintegrin and metalloproteinase domain-containing prot.10
Adam17	Disintegrin and metalloproteinase domain-containing prot.17
ADAM9	Disintegrin and metalloproteinase domain-containing prot.9
ADD1	Alpha-adducin
ADH1	Alcohol dehydrogenase class-1
ADH3	Alcohol dehydrogenase class-3
ADH5	Alcohol dehydrogenase class-5
AHCY	Adenosylhomocysteinase
AHCYL2	Adenosylhomocysteinase like 2
AHNAK	Neuroblast differentiation-associated protein AHNAK
AHSG	Alpha-2-HS-glycoprotein
AI314180 / ECPAS	Ecm29 proteasome adaptor and scaffold
AIFM1	Apoptosis-inducing factor
AIP	AH receptor-interacting protein
AK1	Adenylate kinase isoenzyme 1
AKAP8	A-kinase anchoring protein8
AKAP9	A-kinase anchoring protein9
AKR1A1	Alcohol dehydrogenase [NADP(+)]
AKR1B1	Aldose reductase
AKR1B8	Aldose reductase-related protein 1
AKR1C1	Aldo-keto reductase family 1 member C1
AKR1C12l1	Aldo-keto reductase family 1, C12-like 1
AKR1C2	Aldo-keto reductase family 1 member C2
AKR1C3	Aldo-keto reductase family 1 member C3
AKR2	Ankyrin repeat-containing protein2
AKR7A2	Aldo-keto reductase family 7 member A2
ALB	Serum albumin
ALDH2	Alldehyde dehydrogenase 2 family member
ALDOA	Fructose-bisphosphate aldolase A
ALDOC	Fructose-bisphosphate aldolase C
ANKFY1	Rabankyrin-5
ANLNl1	anillin, actin binding protein-like 1
ANXA1	Annexin A1
ANXA11	Annexin A11
ANXA2	Annexin A2
ANXA3	annexin A3
ANXA5	Annexin A5
ANXA6	Annexin A6
ANXA6	Annexin A6
ANXA7	Annexin A7
AOC1	Amiloride-sensitive amine oxidase
AP1B1	AP-1 complex subunit beta-1
AP2A1	AP-2 complex subunit alpha-1
AP2A2	AP-2 complex subunit alpha-2
AP2B1	AP-2 complex subunit beta
AP2M1	AP-2 complex subunit mu
AP2S1	AP-2 complex subunit sigma
AP3B1	AP-3 complex subunit beta-1
APEH	Acylamino-acid-releasing enzyme
APEX1	DNA-(apurinic or apyrimidinic site) lyase
APOB	Apolipoprotein B-100
APRT	Adenine phosphoribosyltransferase
AQP4	Aquaporin 4
ARCN1	Archain 1

Table S6 continued

Synonym	Full name
AREG	Amphiregulin
ARF3	ADP-ribosylation factor 3
ARF4	ADP-ribosylation factor 4
ARF5	ADP-ribosylation factor 5
ARFIP1	ADP-ribosylation factor interacting protein 1
ARHGDIA	Rho GDP dissociation inhibitor alpha
ARID1A	AT-rich interaction domain 1A
ARL8A	ADP-ribosylation factor-like protein 8A
ARMC10	Armadillo repeat-containing protein 10
ARPC1B	Actin-related protein 2/3 complex subunit 1B
ARPC3	Actin-related protein 2/3 complex subunit 3
ARRDC1	Arrestin domain-containing protein 1
ASAP1	Arf-GAP with SH3 domain, ANK repeat and PH domain-cont. protein 1
ASCL4	acyl-CoA synthetase long chain family memb. 4
ASCT2	Alanine/serine/cysteine/threonine transporter 2
ASL	Argininosuccinate lyase
ASNS	Asparagine synthase
ASS1	Argininosuccinate synthase
ATIC	5-aminoimidazole-4-carboxamide ribonucleotide formyltransferase/IMP cyclohydrolase
ATP1A3	Sodium/potassium-transporting ATPase subunit alpha-3
ATP1B1	Sodium/potassium-transporting ATPase subunit beta-1
ATP1B2	ATPase, Na+/K+ transporting, beta 2 polypeptide
ATP2A2	Sarcoplasmic/endoplasmic reticulum calcium ATPase 2
ATP2B4	Plasma membrane calcium-transporting ATPase 4
ATP5A1	ATP synthase, H+ transporting, mitochondrial F1 complex, alpha subunit 1
ATP5B	ATP synthase subunit beta
ATP5EP2	ATP synthase subunit epsilon-like protein, mitochondrial
ATP5L	ATP synthase subunit g, mitochondrial
ATP5S	ATP synthase, H+ transporting, mitochondrial Fo complex subunit s
ATP6AP2	Renin receptor
ATP6V0D1	V-type proton ATPase subunit d 1
ATP6V1B2	V-type proton ATPase subunit B, brain isoform
ATP6V1D	V-type proton ATPase subunit D
ATXN10	Ataxin-10
BANF1	barrier to autointegration factor 1
BCL2L1	BCL2 like 1
BET1	Bet1 golgi vesicular membrane trafficking protein
BET1L	BET1-like protein
BIRC6	baculoviral IAP repeat containing 6
BMP4	Bone morphogenetic protein 4
BMP7	Bone morphogenetic protein 7
BROX	BRO1 domain-containing protein BROX
BUD31	BUD31 homolog
BZW1	basic leucine zipper and W2 domains 1
BZW2	basic leucine zipper and W2 domains 2
C12orf10	Chromosome C12 orf10
C3	Complement C3
C4	Complement C4
C9orf64	UPF0553 protein C9orf64
CAD	DNA fragmentation factor subunit beta
CAND1	Cullin-associated NEDD8-dissociated protein 1
CAP1	Cyclase associated actin cytoskeleton regulatory protein 1
CAPN1	Calpain-1 catalytic subunit
CAPN5	Calpain-5
CAPNS1	Calpain small subunit 1
CARKD / NAXD	NAD(P)HX dehydratase
CASK	Peripheral plasma membrane protein CASK
CASP3	Caspase 3
CAV2	Caveolin2
CBR4	Carbonic reductase 4
CCDC132	Syndetin
CCND1	Cyclin D1
CCS	Copper chaperone for superoxide dismutase
CCT2	T-complex protein 1 subunit beta
CCT3	T-complex protein 1 subunit gamma
CCT4	T-complex protein 1 subunit delta
CCT5	T-complex protein 1 subunit epsilon
CCT6a	Chaperonin containing Tcp1, subunit 6A
CCT7	T-complex protein 1 subunit eta
CCT8	Chaperonin subunit theta
CD151	Tetraspanin CD151
CD166	CD166 antigen ALCAM
CD276	CD276 antigen
CD46	CD46 molecule
CD55	Complement decay-accelerating factor
CD59	CD59 (blood group) molecule
CD63	Tetraspanin CD63
CD81	Tetraspanin CD81
CD82	Tetraspanin CD82
CD9	Tetraspanin CD9
CD97	CD97 antigen

Table S6 continued

Synonym	Full name
CDH1	Cadherin-1
CDHR1	Cadherin related family member 1
CDK1	Cyclin-dependent kinase 1
CDKN2A	Cyclin dependent kinase inhibitor 2A
CDS1	CDP-diaglycerol synthase 1
CEMIP	Cell migration-inducing and hyaluronan-binding protein
CFL1	Cofilin-1
CFL2	Cofilin-2
CHD4	Chromodomain helicase DNA binding protein4
CHMP1B	Charged multivesicular body protein 1b
CHMP2A	Charged multivesicular body protein 2a
CHMP2B	Charged multivesicular body protein 2b
CHMP3	Charged multivesicular body protein 3
CHMP5	Charged multivesicular body protein 5
CIB1	Calcium and integrin-binding protein 1
CIT	Citron Rho-interacting kinase
CKB	Creatine kinase B-type
CLD2	Claudin-2
CLDN1	Claudin domain-containing protein 1
CLDN2	Claudin-2
CLDN3	Claudin-3
CLDN4	Claudin-4
CLDN7	Claudin-7
CLDN9	Claudin-9
CLIC1	Chloride intracellular channel protein 1
CLIP2	Chloride intracellular channel protein 2
CLTA	Clathrin light chain A
CLTC	Clathrin heavy chain
CLU	Clusterin
CNN2	Calponin-2
CNOT1	CCR4-NOT transcription complex subunit 1
CNPY3	canopy FGF signaling regulator 3
COASY	Bifunctional coenzyme A synthase
COL18A1	Procollagen, type XVIII, alpha 1
COMT	catechol-O-methyltransferase
COPA	Coatomer subunit alpha
COPB2	Coatomer subunit beta 2
COPE	Coatomer subunit epsilon
COPS3	COP9 signalosome complex subunit 3
COPS4	COP9 signalosome complex subunit 4
COPS5	COP9 signalosome complex subunit 5
COPS6	COP9 signalosome complex subunit 6
CORO1C	Coronin 1C
COTL1	Coactosin-like protein
CPSF7	cleavage and polyadenylation specific factor 7
CSD1	copper/zinc superoxide dismutase 1
CSE1	exportin-2
CSKP	Peripheral plasma membrane protein CASK CASK
CSNK1A1	Casein kinase I isoform alpha
CSNK2B	Casein kinase II subunit beta
CTNNB1	Catenin beta-1
CTNND1	Catenin delta-1
CTPS1	CTP synthase 1
CTSZ	Cathepsin Z
CUL1	Cullin-1
CUL3	Cullin-3
CUL4A	Cullin-4A
CXADR	Coxsackievirus and adenovirus receptor
CYB5R3	Cytochrome B5 reductase 3
CYP51A1	Lanosterol 14-alpha demethylase
DAZAP1	DAZ associated protein 1
DCPS	decapping enzyme, scavenger
DCTD	Deoxycytidylate deaminase
DCTN1	Dynactin subunit 1
DCTN3	Dynactin subunit 3
DDAH2	N(G),N(G)-dimethylarginine dimethylaminohydrolase 2
Ddb1	Damage specific DNA binding protein 1
DDOST	Dolichyl-diphosphooligosaccharide--protein glycosyltransferase
DDX1	DEAD-box helicase 1
DDX17	Probable ATP-dependent RNA helicase DDX17
Ddx39a	Spliceosome RNA helicase DDX39A
DDX39B	Spliceosome RNA helicase DDX39B
DDX5	Probable ATP-dependent RNA helicase DDX5
DERA	Deoxyribose-phophate aldolase
DHX9	ATP-dependent RNA helicase A
DIE	Insulin-degrading enzyme
DIP2A	Disco-interacting protein 2 homolog A
DIP2B	Disco-interacting protein 2 homolog B
DIP2C	Disco-interacting protein 2 homolog C
DLG1	Disks large homolog 1

Table S6 continued

Synonym	Full name
DNAJ9	DnaJ homolog subfamily C member 9
DNAJA1	DnaJ homolog subfamily A member 1
DNAJB1	DnaJ homolog subfamily B member 1
DNAJB9	DnaJ homolog subfamily B member 9
DNAJC13	DnaJ homolog subfamily C member 13
DNAJC2	DnaJ homolog subfamily C member 2
DNM1L	Dynamin-1-like protein
DNM2	Dynamin-2
DNMT1	DNA methyltransferase
DNPEP	Aspartyl aminopeptidase
DNPH1	2'-deoxynucleoside 5'-phosphate N-hydrolase 1
DPEP1	Dipeptidase 1
DPP4	Dipeptidyl peptidase 4
DPP7	Dipeptidyl peptidase 2
DRG2	Developmentally regulated GTP binding protein 2
DSG2	Desmoglein-2
DSTN	Destrin
DTD1	D-tyrosyl-tRNA deacylase 1
DUSP3	Dual specificity phosphatase 3
DUT	Deoxyuridine 5'-triphosphate nucleotidohydrolase, mitochondrial
DYNC1H1	dynein cytoplasmic 1 heavy chain 1
DYNC1I2	dynein cytoplasmic 1 intermediate chain 2
DYNLL1	Dynein light chainLC8-type 1
DYNLL2	Dynein light chain 2, cytoplasmic
DYNLT1	Dynein light chain Tctex-type 1
ECHDC1	ethylmalonyl-CoA decarboxylase 1
ECM29	Proteasome-associated protein ECM29 homolog
EEF1A1	Elongation factor 1-alpha 1
EEF1D	Elongation factor 1-delta
EEF1E1	eukaryotic translation elongation factor 1 epsilon 1
EFL1	Elongation factor-like GTPase 1
EFNB1	Ephrin-B1
EFNB2	Ephrin-B2
EGFR	Epidermal growth factor receptor
EHD1	EH domain-containing protein 1
EHD3	EH domain-containing protein 3
EHD4	EH domain-containing protein 4
EIF1AX	Eukaryotic translation initiation factor 1A, X-chromosomal
EIF2S1	Eukaryotic translation initiation factor 2 subunit 1
EIF2S3	Eukaryotic translate. initiation factor2 subunit 3
EIF2S3X	eukaryotic translation initiation factor 2, subunit 3
EIF3A	Eukaryotic translation initiation factor 3 subunit A
EIF3B	Eukaryotic translation initiation factor 3 subunit B
EIF3C	Eukaryotic translation initiation factor 3 subunit C
EIF3D	Eukaryotic translation initiation factor 3 subunit D
EIF3E	Eukaryotic translation initiation factor 3 subunit E
EIF3F	Eukaryotic translation initiation factor 3 subunit F
EIF3H	Eukaryotic translation initiation factor 3 subunit H
EIF3I	Eukaryotic translation initiation factor 3 subunit I
EIF3K	Eukaryotic translation initiation factor 3 subunit K
EIF3L	Eukaryotic translation initiation factor 3 subunit L
EIF3M	Eukaryotic translation initiation factor 3 subunit M
EIF3T	eukaryotic translation initiat. factor 3, subunit 10 theta
EIF4A2	Eukaryotic initiation factor 4A-II
EIF4E	Eukaryotic translation initiation factor 4E
EIF4E2	Eukaryotic translation initiation factor 4E type 2
EIF4G1	Eukaryotic translation initiation factor 4G 1
EIF5A	Eukaryotic translation initiation factor 5A
ELAVL1	ELAV like RNA binding protein 1
EMB	Embigin
ENO1	Enolase 1
ENO2	Enolase gamma
ENO3	Enolase beta
EPB41L1	Band 4.1-like protein 1
EPCAM	Epithelial cell adhesion molecule
EPHA2	Ephrin type-A receptor 2
EPHB2	Ephrin type-B receptor 2
EPHB3	Ephrin type-B receptor 3
EPHB4	Ephrin type-B receptor 4
EPHX1	Epoxide hydrolase 1
EPRS	Bifunctional glutamate/proline--tRNA ligase
EPS8L3	Epidermal growth factor receptor kinase substrate 8-like protein 3
ERH	Enhancer of rudimentary homolog
ERLIN2	Erlin-2
ERMP1	Endoplasmic reticulum metallopeptidase 1
ERO1A	ERO1-like protein alpha
ERP29	Endoplasmic reticulum protein 29
ESYT1	extended synaptotagmin 1
EZR	Ezrin
F2	Prothrombin
F5	Coagulation factor V

Table S6 continued

Synonym	Full name
FABP5	Fatty acid binding protein 5
FAM120A	Constitutive coactivator of PPAR-gamma-like protein 1
FAM175B	family with sequence similarity 175 member B
FAM213A	family with sequence similarity 213, member A
FAM3C	family with sequence similarity 3 member C
FAM49B	family with sequence similarity 49 member B
FANCD2	Fanconi anemia group D2 protein homolog
FANCI	Fanconi anemia group I protein
FAR1	Fatty acyl-CoA reductase 1
FARP1	FERM, RhoGEF and pleckstrin domain-containing protein 1
FARP2	FERM, RhoGEF and pleckstrin domain-containing protein 2
FARSA	Phenylalanine--tRNA ligase alpha subunit
FARSB	Phenylalanine--tRNA ligase beta subunit
FASN	Fatty acid synthase
FAT1	Protocadherin Fat 1
FBXO6	F-box protein 6
FDPS	farnesyl diphosphate synthase
FGB	fibrinogen beta chain
FKBP4	Peptidyl-prolyl cis-trans isomerase FKBP4
FLII	flightless-1 homolog
FLNA	Filamin-A
FLNB	Filamin-B
FLNC	Filamin-C
FLOT1	Flotillin-1
FLOT2	Flotillin-2
FN1	Fibronectin
FPRP	Prostaglandin F2 receptor negative regulator
FRIL	Ferritin light chain FTL
FSCN1	Fascin
FTH1	Ferritin heavy chain
FTL1	Ferritin light chain 1
FUBP3	Far upstream element-binding protein 3
FYN	Tyrosine-protein kinase Fyn
FZD3	Frizzled class receptor 3
G6PC	Glucose-6-phosphatase
G6PD	glucose-6-phosphate dehydrogenase
GAA	Lysosomal alpha-glucosidase
GAPDH	Glyceraldehyde-3-phosphate dehydrogenase
GAPVD1	GTPase-activating protein and VPS9 domain-containing protein 1
GARS	Glycine--tRNA ligase
GART	Phosphoribosylglycinamide formyltransferase
GC	Vitamin D-binding protein
GCA	Grancalcin
GCC2	GRIP and coiled-coil domain containing 2
GCN1(LL1)	GCN1, eIF2 alpha kinase activator homolog
GDA	Guanine deaminase
GDI1	Rab GDP dissociation inhibitor alpha
GFPT2	Glutamine--fructose-6-phosphate aminotransferase [isomerizing] 2
GGH	Gamma-glutamyl hydrolase
GGT1	gamma-glutamyl transpeptidase
GHITM	Growth hormone-inducible transmembrane protein
GLB1	Beta-galactosidase
GLG1	Golgi apparatus protein 1
GLS	Glutaminase kidney isoform
GMPS	guanine monophosphate synthase
GNA11	Guanine nucleotide-binding protein subunit alpha-11
GNAI3	Guanine nucleotide-binding protein G(k) subunit alpha
GNAS	Guanine nucleotide-binding protein G(s) subunit alpha XLas
GNB1	Guanine nucleotide-binding protein G(I)/G(S)/G(T) subu. beta1
GNB2	Guanine nucleotide-binding protein G(I)/G(S)/G(T) subu. beta-2
GNB4	Guanine nucleotide-binding protein subunit beta-4
GNG5	G protein subunit gamma 5
GOLGA4	Golgin subfamily A member 4
GOLGA7	Golgin subfamily A member 7
GOLGB1	Golgin subfamily B member 1
GOT2	Aspartate aminotransferase
GPA33	Cell surface A33 antigen
GPI	Glucose-6-phosphate isomerase
GPRC5A	Retinoic acid-induced protein 3
GPRC5C	G-protein coupled receptor family C group 5 member C
GRB2	Growth factor receptor bound protein 2
GRHPR	Glyoxylate reductase/hydroxypyruvate reductase
GRWD1	Glutamate rich WD repeat containing 1
GSN	Gelsolin
GSS	Glutathione synthetase
GSTP1	Glutathione S-transferase pi 1
GSTT3	Glutathione S transferase T3
H2AFX	H2A histone family, member X
H2AFY	Core histone macro-H2A.1
H2AV	Histone H2A.V H2AFV

Table S6 continued

**Synonym**	**Full name**
HACD3	Very-long-chain (3R)-3-hydroxyacyl-CoA dehydratase 3
HADHB	hydroxyacyl-CoA dehydrogenase, beta subunit
HARS	Histidine--tRNA ligase, cytoplasmic
HBA1	Hemoglobin subunit alpha 1
HBA2	Hemoglobin subunit alpha 2
HCFC1	host cell factor C1
HDAC1	Histone deacetylase 1
HDHD3	Haloacid dehalogenase-like hydrolase domain-containing protein 3
HDLBP	High density lipoprotein bind. protein (Vigilin)
HEPH	Hephaestin
HEXB	Hexosaminidase subunit beta
HIDADH	3-hydroxyisobutyrate dehydrogenase
HIST1H1A	Histone H1.1
HIST1H2AD	Histone H2A type 1-D
HIST1H2B	histone H2B
HIST1H2BB	Histone H2B type 1-B
HIST1H2BC	Histone H2B type 1-C
HIST2AI1	Histone H2A type 1
HIST2BI1	Histone H2B type 1
HIST2H2BE	Histone H2B type 1-E
HIST3H3	Histone H3.1
HISTH2AD	Histone H2A type 1-D
HK2	Hexokinase-2
HM13	Minor histocompatibility antigen H13
HMGCS1	Hydroxymethylglutaryl-CoA synthase, cytoplasmic
HNF1A	HNF1 homeobox A
HNRNP2B1	Heterogeneous nuclear ribonucleoprotein A0
HNRNPA0	Heterogeneous nuclear ribonucleoprotein B1
HNRNPA3	Heterogeneous nuclear ribonucleoprotein A3
HNRNPAB	Heterogeneous nuclear ribonucleoprotein A/B
HNRNPC	Heterogeneous nuclear ribonucleoproteins C1/C2
HNRNPDL	Heterogeneous nuclear ribonucleoprotein D-like
HNRNPH2	Heterogeneous nuclear ribonucleoprotein H2
HNRNPH3	Heterogeneous nuclear ribonucleoprotein H3
HNRNPL	Heterogeneous nuclear ribonucleoprotein L
HNRNPM	heterogeneous nuclear ribonucleoprotein M
HNRNPR	Heterogeneous nuclear ribonucleoprotein R
HNRNPUL2	Heterogeneous nuclear ribonucleoprotein U-like protein 2
HNRPD	RNA binding protein p42 AUF1
HNRPK	Heterogeneous nuclear ribonucleoprotein K
HP1BP3	Heterochromatin protein 1-binding protein 3
HRAS	HRas proto-oncogene, GTPase
HRSP12	Ribonuclease UK114
HSBP1	Heat shock factor binding protein 1
HSD17B10	Hydroxysteroid 17-beta dehydrogenase 10
HSD17B11	Estradiol 17-beta-dehydrogenase 11
HSD17B12	estradiol 17-beta-dehydrogenase 12
HSD17B4	Peroxisomal multifunctional enzyme type 2
HSP4	Heat shock 70 kDa protein D
HSP71	Heat shock protein71
HSP90AA1	Heat shock protein HSP 90-alpha
HSP90B1	Endoplasmin
HSPA1A	DnaK-type molecular chaperone hsp72-ps1
HSPA1B	Heat shock protein 70kd 1B
HSPA1L	Heat shock 70 kDa protein 1-like
HSPA2	Heat shock-related 70 kDa protein 2
HSPA4	Heat shock 70 kDa protein 4
HSPA4L	Heat shock 70 kDa protein 4L
HSPA5	78 kDa glucose-regulated protein
HSPA9	Heat shock protein 90, alpha
HSPB1	Heat shock protein beta-1
HSPB11	Intraflagellar transport protein 25 homolog
HSPD1	Heat shock protein family D (Hsp60) member 1
HSPH1	Heat shock protein 105 kDa
HTRA1	Serine protease HTRA1
HTT	Huntingtin
HUWE1	HECT, UBA and WWE domain containing 1, E3 ubiquitin protein ligase
HYOU1	Hypoxia up-regulated 1
IARS	Isoleucyl-tRNA synthetase
IDE	Insulin-degrading enzyme
IDH1	Isocitrate dehydrogenase (NADP(+)) 1, cytosolic
IDH3A	Isocitrate dehydrogenase subunit alpha
IGF2R	Cation-independent mannose-6-phosphate recept.
IGSF3	Immunoglobulin superfamily member 3
IGSF8	Immunoglobulin superfamily member 8
IL24	Interleukin-24
IL33	interleukin-33
ILF2	Interleukin enhancer-binding factor 2
ILF3	Interleukin enhancer-binding factor 3
IMMT	MICOS complex subunit Mic60
IMPDH2	Inosine-5'-monophosphate dehydrogenase 2

Table S6 continued

Synonym	Full name
IONP1	lon protease homolog
IPO4	Importin-4
IQGAP1	IQ motif containing GTPase activating protein 1
IQGAP2	Ras GTPase-activating-like protein IQGAP2
IST1	IST1 homolog
ITGA2	Integrin alpha-2
ITGA3	Integrin alpha-3
ITGA6	Integrin alpha-6
ITGAV	Integrin alpha-V
ITGB1	Integrin beta-1
ITGB2	Integrin beta-2
ITGB3	Integrin beta-3
ITGB4	Integrin beta-4
ITGB5	Integrin beta-5
ITIH2	Inter-alpha-trypsin inhibitor heavy chain H2
ITIH3	Inter-alpha-trypsin inhibitor heavy chain H3
ITPR2	Inositol 1,4,5-trisphosphate receptor type 2
ITPR3	Inositol 1,4,5-trisphosphate receptor type 3
JAM1	Junctional adhesion molecule A F11R
JH06 2394	Transitional endoplasmic reticulum ATPase
JH06 3331	Diphosphomevalonate decarboxylase
JH06 4067	Dihydroorotase
JH06 4591	Fructosamine ketosamine-3-kinase
JH06 5301	Glycine-tRNA ligase
JUP	Junction plakoglobin
KARS	Lysine--tRNA ligase
KB15	Keratin, type II cytoskeletal cochleal
KDM1A	Lysine-specific histone demethylase 1A KDM1A
KIF13B	Kinesin-like protein KIF13B
KIF15	Kinesin family member 15
KIF5B	Kinesin family member 5B
KNTC1	Kinetochore associated 1
KPNB1	Importin subunit beta-1
KRAS	GTPase KRas
KRT1	Keratin, type II cytoskeletal 1
KRT10	Keratin, type I cytoskeletal 10
KRT14	Keratin, type I cytoskeletal 14
KRT15	Keratin, type I cytoskeletal 15
KRT16	keratin, type I cytoskeletal 16
KRT17	keratin, type I cytoskeletal 17
KRT18	keratin, type I cytoskeletal 18
KRT19	Keratin, type I cytoskeletal 19
KRT20	Keratin, type I cytoskeletal 20
KRT33b	keratin, type I cuticular Ha3-II
KRT4	Keratin, type II cytoskeletal 4
KRT42	keratin, type I cytoskeletal 42
KRT5	Keratin, type II cytoskeletal 5
KRT6A	Keratin, type II cytoskeletal 6A
KRT6B	Keratin, type II cytoskeletal 6B
KRT7	Keratin, type II cytoskeletal 7
KRT73	Keratin, type II cytoskeletal 73
KRT75	Keratin, type II cytoskeletal 75
KRT76	Keratin, type II cytoskeletal 76
KRT77	Keratin, type II cytoskeletal 77
KRT8	Keratin, type II cytoskeletal 8
L2HGDH	L-2-hydroxyglutarate dehydrogenase
LAD1	Ladinin 1
LAMA5	Laminin subunit alpha-5
LAMB1	Laminin subunit beta-1
LAMC1	Laminin subunit gamma-1
LAMTOR1	Ragulator complex protein LAMTOR1
LAP2A	Lamina-associated polypeptide 2, isoform alpha
LAP2B	Lamina-associated polypeptide 2, isoforms beta/gamma
LBR	Lamin-B receptor
LCN2	Neutrophil gelatinase-associated lipocalin
LDHA	L-lactate dehydrogenase
LDHAL6B	L-lactate dehydrogenase A-like 6B
LDHB	L-lactate dehydrogenase B chain
LDLR	Low density lipoprotein receptor
LGALS1	Galectin-1
LGALS3	Galectin-3
LGALS3BP	Galectin-3-binding protein
LIN7C	Protein lin-7 homolog C
LIPH	Lipase member H
LMNA	Lamin A
LMO2	LIM domain 2
LNPEP	Leucyl-cystinyl aminopeptidase
LOC100359539	ribonucleotide reductase M2 polypeptide
LOC100363782	RAB1B, RAS oncogene family like
LOC100910308	multifunctional protein ADE2-like
LOC100910366	uncharacterized

Table S6 continued

Synonym	Full name
LOC100910554	histone H2A type 1-like
LOC100910688	PURH-like
LOC100911244	large neutral amino acid transporter small subunit 1-like
LOC100911966	60S ribosomal protein L7a-like
LOC100912445	leucyl-cystinyl aminopeptidase-like
LOC102551071	myosin light polypeptide 6-like
LOC102551657	Sialic acid synthase-like
LOC102551877	mRNA turnover protein 4 homolog
LOC102552480	histone H2B type 2-E
LOC102554637	anionic trypsin-2-like
LOC103694878	glutathione S-transferase theta-2-like
LOC286987	Hemiferrin
LONP1	Lon peptidase 1
LRBA	LPS responsive beige-like anchor protein
LRP1	LDL receptor related protein 1
LRPPRC	Leucine rich pentatricopeptide repeat containing
LRRC57	Leucine-rich repeat-containing protein 57
LRRC59	Leucine-rich repeat-containing protein 59
LSM1	U6 snRNA-associated Sm-like protein LSm1
LSM4	U6 snRNA-associated Sm-like protein LSm4
LSR	Lipolysis-stimulated lipoprotein receptor
LTA4H	Leukotriene A-4 hydrolase
LTN1	listerin E3 ubiquitin protein ligase 1
LUM	Lumican
LY75	Lymphocyte antigen 75
LYN	Tyrosine-protein kinase Lyn
LYPLA1	Lysophospholipase 1
M6PR	Mannose-6-phosphate receptor, cation dependent
MACF1	Microtubule-actin crosslinking factor 1
MAN1A1	Mannosyl-oligosaccharide 1,2-alpha-mannosidase IA
MAN2B1	Lysosomal alpha-mannosidase
MAP2K1	Dual specificity mitogen-activated protein kinase kinase 1
MAP4	Microtubule associated protein 4
MAP4K4	Mitogen-activated protein kinase kinase 4
MAPK1	Mitogen-activated protein kinase !
MAT1A	Methionine adenosyltransferase 1A
MATR3	Matrin-3
MBOAT7	Lysophospholipid acyltransferase 7
MCM2	DNA replication licensing factor MCM2
MCM6	Minichromosome maintenance complex component 6 [
MCU	Mitochondrial calcium uniporter
MDH2	Malate dehydrogenase 2
MDN1	Midasin AAA ATPase 1
MELTF	Melanotransferrin
MEP1A	Meprin A subunit alpha
MET	Hepatocyte growth factor receptor
MFGE8	Lactadherin
MFI2 /MELTF	Melanotransferrin
MINK1	Misshapen-like kinase 1
MITD1	MIT domain-containing protein 1
MLEC	Malectin
MOB1A	MOB kinase activator 1A
MRPL17	39S ribosomal protein L17, mitochondrial
MRPL19	39S ribosomal protein L19, mitochondrial
MRPL22	39S ribosomal protein L22, mitochondrial
MRPL28	39S ribosomal protein L28, mitochondrial
MRPL43	39S ribosomal protein L43, mitochondrial
MRTO4	MRT4 homolog, ribosome maturation factor
MSN	Moesin
MTAP	Methythioadenosine phosphorylase
MTHFD1	Methylenetetrahydrofolate dehydrogenase, cyclohydrolase and formyltetrahydrofolate synthetase 1
MTHFD1L	Monofunctional C1-tetrahydrofolate synthase, mitochondrial
MTOR	Mechanistic target of rapamycin kinase
MUC13	Mucin-13
MUC4	Mucin 4
MVB12A	Multivesicular body subunit 12A
MVB12B	Multivesicular body subunit 12B
MVP	Major vault protein
MYC	Oncogene, bHLH transcription factor
MYH10	Myosin, heavy polypeptide 10
MYH11	Myosin-11
MYH13	Myosin-13
MYH14	Myosin heavy chain 14
MYH9	Myosin, heavy polypeptide 9
MYL2	Myosin light chain 2
MYL6L	Myosin light polypeptide 6
MYO10	Myosin-10
MYO1B	Unconventional myosin-Ib
MYO1C	Unconventional myosin-Ic
MYO1E	Myosin-1E
MYOF	Myoferlin

Table S6 continued

Synonym	Full name
NAGLU	Alpha-N-acetylglucosaminidase
NAMPT	Nicotinamide phosphoribosyltransferase
NANS	Sialic acid synthase
NAP1L4	Nucleosome assembly protein 1-like 4
NAPA	NSF attachment protein alpha
NARS	Asparagine--tRNA ligase
NBAS	Neuroblastoma amplified sequence
NCBP1	Nuclear cap-binding protein subunit 1
NCL	Nucleolin
NCSTN	Nicastrin
NDUFA5	NADH:ubiquinone oxidoreductase subunit A5
NDUFB10	NADH:ubiquinone oxidoreductase subunit B10
NDUFS2	NADH dehydrogenase [ubiquinone] iron-sulfur protein 2, mitochondrial
NDUFS5	NADH dehydrogenase [ubiquinone] iron-sulfur protein 5
NEMF	Nuclear export mediator factor
NEU1	Sialidase-1
NIPSNAP3A	Protein NipSnap homolog 3A
NMT1	Glycylpeptide N-tetradecanoyltransferase 1
NNT	Nicotinamide nucleotide transhydrogenase
NONO	Non-POU domain-containing octamer-binding protein
NOP2	NOP2 nucleolar protein
NPC1	NPC intracellular cholesterol transporter 1
Npepps	aminopeptidase puromycin sensitive
NPM1	Nucleophosmin
Nqo1	NAD(P)H quinone dehydrogenase 1
NRAS	NRAS proto-oncogene, GTPase
NSF	N-ethylmaleimide sensitive factor, vesicle fusing ATPase
NT5C3A	Cytosolic 5'-nucleotidase 3A
NTMT1	N-terminal Xaa-Pro-Lys N-methyltransferase 1
NUMA1	Nuclear mitotic apparatus protein 1
NUP210	Nuclear pore membrane glycoprotein 210
OAF	out at first homolog
OAT	Ornithine aminotransferase, mitochondrial
OGDH	2-oxoglutarate dehydrogenase, mitochondrial
OGT	O-linked N-acetylglucosamine transferase
OLFM4	Olfactomedin-4
OSBP	Oxysterol-binding protein 1
P4HB	Prolyl 4-hydroxylase subunit beta
PA2G4	Proliferation-associated protein 2G4
PABPC4	Polyadenylate-binding protein 4
PAICS	Multifunctional protein ADE2
PAK1	Serine/threonine-protein kinase PAK 1
PARK7	Parkinsonism associated deglycase
PARP1	Poly [ADP-ribose] polymerase 1
PARP4	Poly [ADP-ribose] polymerase 4
PBDC1	Polysaccharide biosynthesis domain containing 1
PC	Pyruvate carboxylase, mitochondrial
PCBD1	Pterin-4 alpha-carbinolamine dehydratase 1
PCBD2	Pterin-4 alpha-carbinolamine dehydratase 2
PCBP3	Poly(rC)-binding protein 3
PCNA	Proliferating cell nuclear antigen
PDAP1	28 kDa heat- and acid-stable phosphoprotein
PDCD10	Programmed cell death 10
PDCD4	Programmed cell death 4
PDCD6	Programmed cell death 6
PDCD6IP	Programmed cell death 6-interacting protein
PDCL3	Phosducin like 3
PDIA3	Protein disulfide isomerase family A member 3
PDIA4	Disulfide-isomerase A4
PDIA6	Protein disulfide isomerase family A member 6
PDLIM1	PDZ and LIM domain protein 1
PDLIM5	PDZ and LIM domain 5
PDXK	Pyridoxal kinase
PEA-15	Astrocytic phosphoprotein
PECR	Peroxisomal trans-2-enoyl-CoA reductase
PEPD	Xaa-Pro dipeptidase
PFAS	Phosphoribosylformylglycinamidine synthase
PFN1	Profilin-1
PFN2	Profilin-2
PGAM1	Phosphoglycerate mutase 1
PGK1	Phosphoglycerate kinase 1
PGM1	Phosphoglucomutase-1
PHB2	Prohibitin-2
PKM	Pyruvate kinase PKM
PKP3	Plakophilin-3
PLAUR	Urokinase plasminogen activator surface receptor
PLEC	Plectin
PLEKHB2	Pleckstrin homology domain-containing family B member 2
PLOD1	Procollagen-lysine,2-oxoglutarate 5-dioxygenase 1
PLPP2	Phospholipid phosphatase 2

Table S6 continued

Synonym	Full name
PLSCR1	Phospholipid scramblase 1
PLSCR3	Phospholipid scramblase 3
PLXNB2	Plexin-B2
PNPT1	Polyribonucleotide nucleotidyltransferase 1, mitochondrial
POF1B	Protein POF1B
POLA1	DNA polymerase alpha catalytic subunit
POLR1C	RNA polymerase I and III subunit C
POLR2B	DNA-directed RNA polymerase II subunit RPB2
POTEF	POTE ankyrin domain family member F
PPA1	pyrophosphatase 1
PPIA	Peptidyl-prolyl cis-trans isomerase A
PPIB	Peptidyl-prolyl cis-trans isomerase B
PPID	peptidylprolyl isomerase D
PPIDL1	peptidylprolyl isomerase D-like 1
PPL	Periplakin
PPL/ D4A5T8	Periplakin
PPM1D	Protein phosphatase, Mg2+/Mn2+ dependent 1D
PPM1G	Protein phosphatase 1G
PPP1CB	Serine/threonine-protein phosphatase PP1-beta catalytic subunit
PPP1CC	Serine/threonine-protein phosphatase
PPP1R7	Protein phosphatase 1 regulatory subunit 7
PPP2CA	Serine/threonine-protein phosphatase 2A catalytic subunit alpha isoform
PPP2R1B	Serine/threonine-protein phosphatase subunit A beta
Ppp3ca	protein phosphatase 3 catalytic subunit alpha
PRDX2	Peroxiredoxin-2
PRDX3	Peroxiredoxin-3
PRKAR2A	Protein kinase cAMP-dependent type II regulatory subunit alpha
PRKCSH	Glucosidase 2 subunit beta
PRKCZ	Protein kinase C zeta
PRMT1	Protein arginine N-methyltransferase 1
PROM1	Prominin-1
PRPF19	Pre-mRNA-processing factor 19
PRPS1	Ribose-phosphate pyrophosphokinase 1
PRPSAP1	Phosphoribosyl pyrophosphate synthetase associated protein 1
PRPSAP2	Pyrophosphate synthase-assoc. protein2
PRSS23	Serine protease 23
PSAT1	Phosphoserine aminotransferase
PSMA1	Proteasome subunit alpha type-1
PSMA2	Proteasome subunit alpha type-2
PSMA3	Proteasome subunit alpha type-3
PSMA4	Proteasome subunit alpha type
PSMA6	Proteasome subunit alpha type-6
PSMA7	Proteasome subunit alpha type-7
PSMB10	Proteasome subunit beta type 10
PSMB3	Proteasome subunit beta type 3
PSMB5	Proteasome subunit beta type 5
PSMB6	Proteasome subunit beta type-6
PSMB7	Proteasome subunit beta type-7
PSMB8	Proteasome subunit beta type-8
PSMB9	Proteasome subunit beta type
PSMC2	26S protease regulatory subunit 7
PSMD1	26S proteasome non-ATPase regulatory subunit 1
PSMD11	26S proteasome non-ATPase regulatory subunit 11
PSMD12	26S proteasome non-ATPase regulatory subunit 12
PSMD13	26S proteasome non-ATPase regulatory subunit 13
PSMD3	26S proteasome non-ATPase regulatory subunit 3
PSMD5	26S proteasome non-ATPase regulatory subunit 5
PSMD6	26S proteasome non-ATPase regulatory subunit 6
PSMD7	26S proteasome non-ATPase regulatory subunit 7
PSME1	Proteasome activator complex subunit 1
PSME2	Proteasome activator complex subunit 2
PTBP3	Polypyrimidine tract-binding protein 3
PTCD3	Pentatricopeptide repeat domain-containing protein 3, mitochondrial
PTDSS1	Phosphatidylserine synthase 1
PTGFB	Platelet derived growth factor subunit B
PTGFRN	Prostaglandin F2 receptor negative regulator
PTGR1	Prostaglandin reductase 1
PTGS2	Prostaglandin-endoperoxide synthase 2
PTP4A1	Protein tyrosine phosphatase type IVA 1
PTP4A2	Protein tyrosine phosphatase type IVA 2
PTPRA	Receptor-type tyrosine-protein phosphatase alpha
PTPRF	Receptor-type tyrosine-protein phosphatase F
PTRF	Polymerase I and transcript release factor
PUS7	Pseudouridylate synthase 7 homolog
PVR	Tumor-associated glycoprotein E4, poliovirus receptor
PYCR1	Pyrroline-5-carboxylate reductase 1, mitochondrial
PYCR2	Pyrroline-5-carboxylate reductase 2
PYCRL	Pyrroline-5-carboxylate reductase 3
PYGB	Glycogen phosphorylase, brain form
PYGL	Glycogen phosphorylase, liver form

Table S6 continued

**Synonym**	**Full name**
Q4AEF8 /COPG1	Coatomer protein complex subunit gamma 1
QDPR	Dihydropteridine reductase
QPCT	Glutaminyl-peptide cyclotransferase
QSOX1	Sulfhydryl oxidase 1
RAB10	Ras-related protein Rab-10
RAB11B	Ras-related Rab-11B
RAB14	Rab14, member RAS oncogene family
RAB18	Ras-related protein Rab-18
RAB22A	Ras-related protein Rab-22A
Rab23	Ras-related protein Rab-23
RAB25	Ras-related protein Rab-25
RAB31	Ras-related protein Rab-31
RAB35	Ras-related protein Rab-35
RAB5A	Ras-related protein Rab-5A
RAB5B	Ras-related protein Rab-5B
RAB5C	Rab5c, member RAS oncogene family
RAB6A	Ras-related protein Rab-6A
RAB6B	Ras-related protein Rab-6B
RAB6C	Ras-related protein Rab-6C
RAB7A	Ras-related protein Rab-7A
RAB8A	Ras-related protein Rab-8A
RAC1	Rac family small GTPase 1
RACK1 / GNB2L1	receptor for activated C kinase 1
RACK1	Receptor of activated protein C kinase 1
RAD23B	UV excision repair protein RAD23 homolog B
RALA	Ras-related protein Ral-A
RALB	Ras-related protein Ral-B
RALY	RNA-binding protein Raly
RANGAP1	Ran GTPase-activating protein 1
RAP1A	Ras-related protein Rap-1A
RAP1B	Ras-related protein Rap-1b
RAP2A	Ras-related protein Rap-2a
RAP2B	Ras-related protein Rap-2b
RAP2C	Ras-related protein Rap-2c
RAS	Ras protein
RBBP9	Putative hydrolase RBBP9
RBM8A	RNA binding motif protein 8A
RBMX	RNA-binding motif protein, X chromosome
Rcc2	regulator of chromosome condensation 2
RDX	Radixin
REEP6	Receptor expression-enhancing protein 6
RFK	Riboflavin kinase
RGD1561636	Similar to 60S ribosomal protein L38
RGD1564698	Similar to ribosomal protein S10
RHEB	GTP-binding protein Rheb
RHOB	Rho-related GTP-binding protein RhoB
RHOF	Rho-related GTP-binding protein RhoF
RHOG	Rho-related GTP-binding protein RhoG
RIF1	Telomere-associated protein RIF1
RL7	60S ribosomal protein L7 RPL7
RNF213	Ring finger protein 213
RNPEP	Aminopeptidase B
ROCK2	Rho associated coiled-coil containing protein kinase 2
ROR1	Inact. tyrosine-protein kinase transmembrane receptor ROR1
RPA1	Ribose-5-phosphate isomerase
RPA3	replication protein A3
RPL10	60S ribosomal protein L10
RPL10A	60S ribosomal protein L10A
RPL12	60S ribosomal protein L12
RPL13	60S ribosomal protein L13
RPL13A	60S ribosomal protein L13A
RPL14	60S ribosomal protein L14
RPL15	60S ribosomal protein L15
RPL15a	60S ribosomal protein L15A
RPL17	60S ribosomal protein L17
RPL18	60S ribosomal protein L18
RPL18A	60S ribosomal protein L18a
RPL2	60S ribosomal protein L2
RPL23A	60S ribosomal protein L23a
RPL26	60S ribosomal protein L26
RPL28	60S ribosomal protein L28
RPL3	60S ribosomal protein L3
RPL30	60S ribosomal protein L30
RPL34	60S ribosomal protein L34
RPL35A	60S ribosomal protein L35a
RPL36	60S ribosomal protein L36
RPL36A	60S ribosomal protein L36a
RPL36L	60S ribosomal protein L36a-like
RPL37A	60S ribosomal protein L37a
RPL4	60S ribosomal protein L4

Table S6 continued

**Synonym**	**Full name**
Rpl5	60S ribosomal protein L5
RPL6	60S ribosomal protein L6
RPL7	60S ribosomal protein L7
RPL7A	60S ribosomal protein L7a
RPL9	60S ribosomal protein L9
RPLP0	60S acidic ribosomal protein P0
RPLP2	60S acidic ribosomal protein P2
RPN1	Ribophorin I
RPN2	Ribophorin II
RPS11	40S ribosomal protein S11
RPS12	40S ribosomal protein S12
RPS15a	40S ribosomal protein S15a
RPS17	40S ribosomal protein S17
RPS2	40S ribosomal protein S2
RPS27A	Ubiquitin-40S ribosomal protein S27a
RPS3	40S ribosomal protein S3
RPS3A	40S ribosomal protein S3a
RPS4X	40S ribosomal protein S4, X isoform
RPS6KA1	Ribosomal protein S6 kinase A1
RPS6KA3	Ribosomal protein S6 kinase A3
RPS7	40S ribosomal protein S7
RPSA	40S ribosomal protein SA
RRAS	Ras-related protein R-Ras
RRBP1	Ribosome-binding protein 1
RSU1	Ras suppressor protein 1
RT1.A1(0)	RT1 class Ia, locus A1
RTN4	Reticulon 4
S100-A14	S100 calcium binding protein A14
S100A4	S100 calcium binding protein A4
SAFB	Scaffold attachment factor B1
SARNP	SAP domain-containing ribonucleoprotein
SARS	Serine--tRNA ligase, cytoplasmic
SART3	spliceosome associated factor 3, U4/U6 recycling protein
SBNO1	strawberry notch homolog 1
SCARB2	Lysosome membrane protein 2
SDCBP	Syntenin-1
SDCBP2	Syntenin-2
SDF4	45 kDa calcium-binding protein
SEC13	Protein SEC13 homolog
SEC23A	Sec23 homolog A, coat complex II component
SEC23B	Sec23 homolog B, coat complex II component
SEC24A	SEC24 homolog A, COPII coat complex comp.
SEC31A	SEC31 homolog A, COPII coat complex component
SEC63	Translocation protein SEC63 homolog
SELENOF	Selenoprotein F
SELH	Selenoprotein H
SEPT2	Septin-2
SEPT7	Septin-7
SEPT9	Septin-9
SERINC5	Serine incorporator 5 SERINC5
SERPINB6	Serpin B6
SERPINB9	Serpin B9
SERPINC1	Serpin family C member 1
SERPINE2	Glia-derived nexin SERPINE2
SERPINF1	Serpin family F member 1
SET	SET nuclear protooncogene
SF3A1	Splicing factor 3a subunit 1
SF3B1	Splicing factor 3b subunit 1
Sf3b3	Splicing factor 3b subunit 3
SFN	stratifin
SFPQ	Splicing factor, proline- and glutamine-rich
SHMT1	Serine hydroxymethyltransferase 1
SLC12A1	Solute carrier family 12 member 1
SLC16A3	Monocarboxylate transporter 4
SLC1A5	Neutral amino acid transporter B(0)
SLC25A3	Solute carrier family 25 member 3
SLC25A4	Solute carrier family 25 member 4
SLC25A5	Solute carrier family 25 member 5
SLC29A1	Equilibrative nucleoside transporter 1
SLC2A1	Solute carrier family 2, facilitated glucose transporter member 1
SLC35A4	SLC35A4 upstream open reading frame protein
SLC3A2	4F2 cell-surface antigen heavy chain
SLC43A1	Large neutral amino acids transporter small subunit 3
SLC44A1	Choline transporter-like protein 1
SLC44A2	Choline transporter-like protein 2
SLC7A1	High affinity cationic amino acid transporter 1
SMARCA4	Transcription activator BRG1
SMARCA5	SWI/SNF-related matrix-associated actin-dep. regulator of chromatin A5
SMARCC1	Matrix assoc., actin dep. regulator chromatin c1
SMC1A	Structural maintenance of chromosomes protein 1A

Table S6 continued

Synonym	Full name
SMC3	Structural maintenance of chromosomes protein 3
SMCHD1	Structural maintenance of chromosomes flexible hinge domain containing 1
SMPDL3B	Acid sphingomyelinase-like phosphodiesterase 3b
SND1	Staphylococcal nuclease and tudor domain containing 1
SNRPF	Small nuclear ribonucleoprotein polypeptide F
SNX2	Sorting nexin-2
SPRYD4	SPRY domain-containing protein 4
SPTAN1	Spectrin alpha chain 1
SPTBN1	Spectrin beta chain, non-erythrocytic 1
SPTBN2	Spectrin beta chain, non-erythrocytic 2
SRC	Proto-oncogene tyrosine-protein kinase Src
SRI	Sorcin
SRPRB	SRP receptor subunit beta
SRRT	Serrate RNA effector molecule homolog
SRSF1	Serine/arginine-rich splicing factor 1
SRSF3	Serine/arginine-rich splicing factor 3
SSB	Lupus La protein homolog
SSBP1	Single-stranded DNA-binding protein, mitochondrial precursor
SSR4	Translocon-associated protein subunit delta
ST14	Suppressor of tumorigenicity 14 protein
STIP1	Stress induced phosphoprotein 1
STK26	Serine/threonine-protein kinase 26
STMN1	Stathmin
STOM	Erythrocyte band 7 integral membrane protein
STT3A	Oligosaccharyltransferase complex, catalytic subunit
STT3B	Oligosaccharyltransferase complex, catalytic subunit
STX12	Syntaxin-12
STX3	Syntaxin-3
STX4	Syntaxin-4
STX7	Syntaxin-7
STXBP2	Syntaxin-binding protein 2
SUPT16H	SPT16 homolog, facilitates chromatin remodeling subunit
SYNCRIP	Synaptotagmin binding cytoplasmic RNA interacting protein
SYNGR2	Synaptogyrin-2
SYVC	Valine--tRNA ligase
TACSTD2	Tumor associated calcium signal transducer 2
TAGLN2	Transgelin-2
TALDO1	Transaldolase 1
TAOK1	TAO kinase 1
TARS	Valine--tRNA ligase
TBCA	Tubulin folding cofactor A
TCERG1	Transcription elongation regulator 1
TCP1	T-complex protein 1 subunit alpha
TECR	Very-long-chain enoyl-CoA reductase
Testin	Testin gene
TFG	Trk-fused gene
TFRC	Transferrin receptor
TGFBI	Transforming growth factor-beta-induced protein ig-h3
TGFBR1	Transforming growth factor beta receptor 1
THBS1	Thrombospondin-1
THOP1	Thimet oligopeptidase 1
TINAGL1	Tubulointerstitial nephritis antigen like 1
TKFC	Triokinase/FMN cyclase
TKT	Transketolase
TLN1	Talin-1
TLN2	Talin-2
TLR4	toll-like receptor 4
TM9SF2	Transmembrane 9 superfamily member 2
TM9SF4	Transmembrane 9 superfamily member 4
TMED10	Transmembrane p24 trafficking protein 10
TMED3	Transmembrane emp24 domain-containing protein 3
TMEM106B	Transmembrane protein 106B
TMEM2	Transmembrane protein 2
TMPRSS4	Transmembrane protease serine 4
TNIK	TRAF2 and NCK-interacting protein kinase
TOM1L1	TOM1-like protein 1
TOM22	Translocase of outer mitochondrial membrane 22
TOMM40	Translocase of outer mitochondrial membrane 40
TOP2A	DNA topoisomerase II alpha
TP53	Tumor protein p53
TP53BP1	Tumor protein p53 binding protein 1
TPI1	Triosephosphate isomerase 1
TPM1	Tropomyosin 1
TPM3	Tropomyosin 3
TPM4	Tropomyosin alpha-4 chain
TPR	Translocated promoter region, nuclear basket protein
TPR0	Nucleoprotein TPR
TRA2B	Transformer-2 protein homolog beta
TRAP1	TNF receptor associated protein 1
TRIM21	E3 ubiquitin-protein ligase TRIM21

Table S6 continued

**Synonym**	**Full name**
TRIM28	Tripartite motif containing 28
TRIP11	Thyroid receptor-interacting protein 11
TRIP12	Thyroid hormone receptor interactor 12
TROVE2	60 kDa SS-A/Ro ribonucleoprotein
TRP / TYRP1	Tyrosinase related protein 1
TRRAP	Transformation/transcription domain-associated protein
TSG101	Tumor susceptibility gene 101 protein
TSN	Translin
TSNAX	Translin-associated protein X
TSPAN1	Tetraspanin-1
TSPAN14	Tetraspanin-14
TSPAN15	Tetraspanin-15
TSPAN3	Tetraspanin-3
TSPAN4	Tetraspanin-4
TSPAN6	Tetraspanin-6
TSPAN8	Tetraspanin-8
TSTA3	GDP-L-fucose synthase
TTYH3	Protein tweety homolog 3
TUBA1A	Tubulin alpha-1A chain
TUBA1B	Tubulin alpha
TUBA1C	Tubulin alpha-1C chain
TUBA4A	Tubulin alpha-4A chain
TUBA8	Tubulin alpha-8 chain
TUBB1	Tubulin beta-1 chain
TUBB2A	Tubulin beta-2A chain
TUBB3	Tubulin beta-3 chain
TUBB4B	Tubulin alpha-4B chain
TUBB5	Tubulin beta-5 chain
TUBB6	Tubulin beta-6 chain
TUFM	Tu translation elongation factor, mitochondrial
TXN	Thioredoxin
TXNDC12	Thioredoxin domain containing 12
TXNL1	Thioredoxin-like protein 1
UBA1	Ubiquitin like modifier activating enzyme 1
UBA6	Ubiquitin like modifier activating enzyme 6
UBB	Polyubiquitin-B
UBC	Ubiquitin C
UBE2I	Ubiquitin conjugating enzyme E2 I
UBE2L3	Ubiquitin-conjugating enzyme E2 L3
UBE2O	(E3-independent) E2 ubiquitin-conjugating enzyme
UBE2V1	Ubiquitin-conjugating enzyme E2 variant 1
UBE2V2	Ubiquitin-conjugating enzyme E2 variant 2
UBR4	Ubiquitin protein ligase E3 component n-recognin 4
UBR5	E3 ubiquitin-protein ligase UBR5
UEVLD	Ubiquitin-conjugating enzyme E2 variant 3
UGDH	UDP-glucose 6-dehydrogenase
UGGT1	UDP-glucose glycoprotein glucosyltransferase 1
UGT1A6	UDP-glucuronosyltransferase 1-6
UGT1A7	UDP-glucuronosyltransferase 1-7
USO1	General vesicular transport factor p115
USP15	Ubiquitin carboxyl-terminal hydrolase
USP47	Ubiquitin carboxyl-terminal hydrolase 47
USP7	Ubiquitin specific peptidase 7
USP9X	Ubiquitin specific peptidase 9 X-linked
UXS1	UDP-glucuronic acid decarboxylase 1
VAMP3	Vesicle associated membrane protein 3
VAT1	Synaptic vesicle membrane protein VAT-1 homolog
VCP	Transitional endoplasmic reticulum ATPase
VDAC1	Voltage-dependent anion-selective channel protein 1
VPS25	Vacuolar protein-sorting-associated protein 25
VPS28	Vacuolar protein sorting-associated protein 28 homolog
VPS36	Vacuolar protein-sorting-associated protein 36
VPS37B	Vacuolar protein sorting-associated protein 37B
VPS37C	Vacuolar protein sorting-associated protein 37C
VPS4B	Vacuolar protein sorting-associated protein 4B
WARS	Tryptophan--tRNA ligase, cytoplasmic
WDR61	WD repeat domain 61
WFS1	Wolframin
XPO1	Exportin-1
XPO2	Exportin-2
XRCC5	X-ray repair cross-complementing protein 5
XRCC6	X-ray repair complementing defective repair
YBX1	Nuclease-sensitive element-binding protein 1
YES1	Tyrosine-protein kinase Yes
YWHAE	14-3-3 protein epsilon
YWHAG	14-3-3 protein gamma
YWHAZ	14-3-3 protein zeta
ZDHHC5	Palmitoyltransferase ZDHHC5

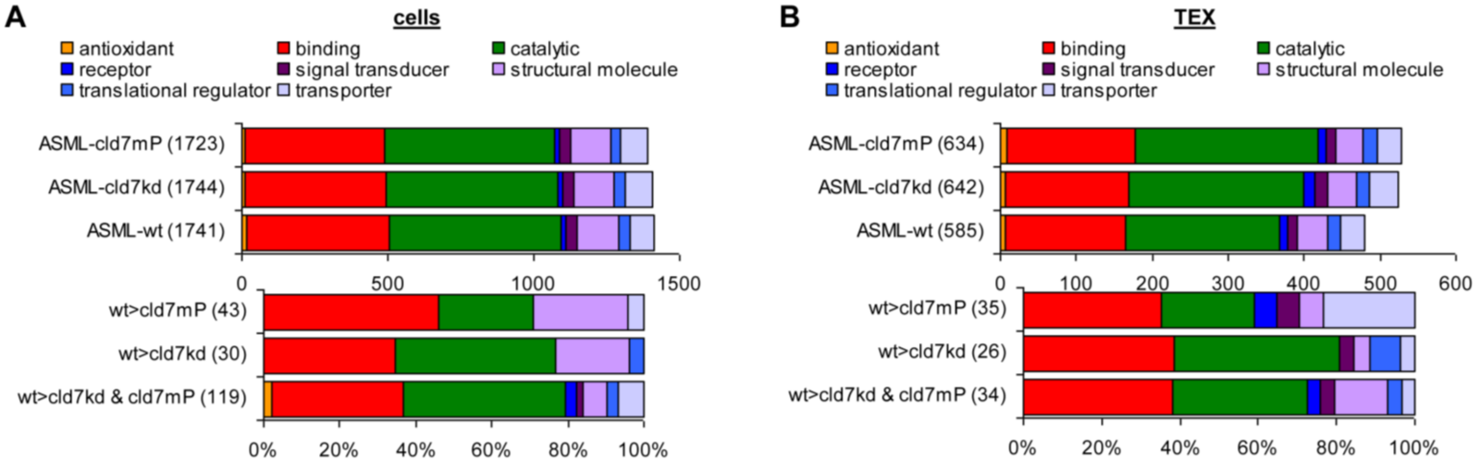
A
cells
antioxidant
binding
catalytic
receptor
signal transducer
structural molecule
translational regulator
transporter
ASML-cld7mP (1723)
ASML-cld7kd (1744)
ASML-wt (1741)
0
500
1000
1500
wt>cld7mP (43)
wt>cld7kd (30)
wt>cld7kd & cld7mP (119)
0%
20%
40%
60%
80%
100%
B
TEX
ASML-cld7mP (634)
ASML-cld7kd (642)
ASML-wt (585)
0
100
200
300
400
500
600
wt>cld7mP (35)
wt>cld7kd (26)
wt>cld7kd & cld7mP (34)

C

Categories: transcription / translation · structure · adhesion / migration · metabolisms · signaling · proteolysis / apoptosis · trafficking / transport

Functional activity of proteins enriched in ASML-wt compared to -cld7kd and -cld7mP cells

transcription / translation: A0A0G2K273, Csd1, Dazap1, Dnajc2, Fancd2, Hcfc1, Hdac1, Kntc1, LOC100359539, LOC100912445, Mcm6, Pola1, Rpl17, Sarnp, Sart3, Snrpf, Trip12, Tufm

structure: Krt20, Tubb3

adhesion / migration: Clip2, D4A5T8, Fat1, HUWE1

metabolisms: A0A0G2K099, Acsl4, Ahcyl2, Akr7a2, Ap2a1, Cbr4, Ccs, Ctsz, Eno2, Far1, Golgb1, Hk2, HUWE1, Immt, LOC103694878, Lrp1, Mia3, Mlec, Ogt, Pdia6, Pecr, Ppp1cc, Prdx3, Smc1a, Usp15, Usp9x

signaling: Ap2a1, Erp29, Fancd2, Itag6, Itgb1, Lgals1, Lrba, Macf1, Mob1a, Polr2b, Pp1cc, Psmb5, Rab31, Rps6ka3, Sfn, Smchd1, Stmn1, Usp9x

proteolysis / apoptosis: Acsl4, Aifm1, Ctsz, Fancd2, Krt20, Lgals1, LOC102554637, Pdia6, Prdx3, Rps6ka3, Trip12

trafficking / transport: A0A0G2K099, Ap2a1, Asct2, Atp1b1, Atp1b2, Ccs, Cope, Ctsz, Esyt1, Golga4, Golgb1, Lrp1, Macf1, Mia3, Napa, Polr2b, Psmb5, Q4aef8, Rab31, Rab6a, Sec23b, Trip11

Functional activity of proteins enriched in ASML-wt compared to -cld7kd cells

transcription / translation: Bud31, Eef1a1, Iars, Pcbd1, RGD1564698, Rpl7, Sf3b1, Usp7

structure: Anlnl1, Flii, Itgb4

adhesion / migration: Drg2, Itga3, Itgb4

metabolisms: Ldlr, Prpsap2, Uba6

signaling: Gdi1, Grb2, Itga3, Itgb4, Rap1a

proteolysis / apoptosis: Serpinb6, Serpinb9, Usp7

trafficking / transport: Dctn1, GDi1, Ldlr, Rab35, Sec23a

Functional activity of proteins enriched in ASML-wt compared to -cld7mP cells

transcription / translation: Bzw1, Cdk1, Ddx5, Eif2s3, Ncl, Psmc2, Rpl10, Rpl13a, Rpl15a, Rps2, Ssb

structure: Krt14, Krt17, Krt6a, Lmna, Tpm4, Tubb1

adhesion / migration: Bzw2, Cotl1, LOC100911966

metabolisms: Eif2s3, Eif4a2, Hdlbp, Hsd17b10, L2hgdh, Ugdh

signaling: Cdk1, Hras, Krt17, Ncl, Psmc2, Rpl13a

proteolysis / apoptosis: D3ZD23, Dnm1l, Lmna, Ncl, Ppidl1

trafficking / transport: Ap2a2, Ap2s1, Arcn1, Arf3, D3ZD23, Dnm1l, Eif2s3, Gcc2, Hdlbp, Kif15, Mcu, Nbas, Sec24a, Tubb1

D

transcription / translation	structure	adhesion / migration	metabolisms	signaling	proteolysis / apoptosis	trafficking / transport

Functional activity of proteins enriched in ASML-wt TEX compared to -cld7kd TEX and -cld7mP TEX

transcription / translation	structure	adhesion / migration	metabolisms	signaling	proteolysis / apoptosis	trafficking / transport
Dcps	Kb15	CD166	Ctps1	CD166	Psmd12	Clic1
EiF2s1	Krt6	Itga2	Ide	Itg2a		Myo10
Eif2s3x	Tuba8	Muc4		Kb15		Ras
Hist1h2b				Myo10		Rdx
Hist2Al1				Ras		
Hist2Bl1				Rdx		
Rpa1						

Functional activity of proteins enriched in ASML-wt TEX compared to -cld7kd TEX

transcription / translation	structure	adhesion / migration	metabolisms	signaling	proteolysis / apoptosis	trafficking / transport
Rpl7			Mdh2	Hspa1a		
Tsnax						

Functional activity of proteins enriched in ASML-wt TEX compared to -cld7mP TEX

transcription / translation	structure	adhesion / migration	metabolisms	signaling	proteolysis / apoptosis	trafficking / transport
Eif3t	Actn1	Lgals3bp	Ggt1	Hsp71	Lta4h	Hsp71
Ggt1	Krt14	PVR	Psat1	Rtn4		Rtn4
H2afx	Krt16			Tuba1b		
	Krt17					
	Krt42					
	Krt5					
	S100a14					
	Tuba1b					

E

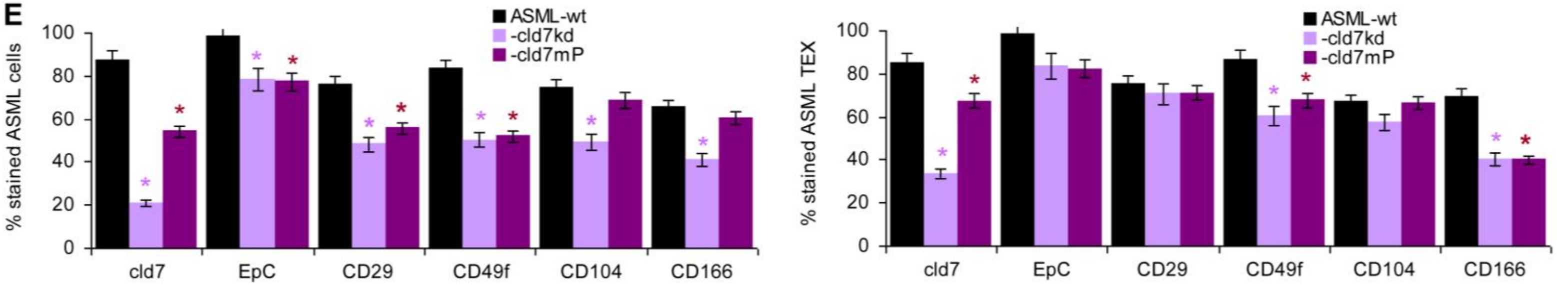

F

Functional activity of proteins enriched in ASML-wt TEX compared to -cld7kd cells

transcription / translation	structure	adhesion / migration	metabolisms	signaling		proteolysis / apoptosis	trafficking / transport
Anxa3	Kb15	Adam10	Apob	Adam10	Kb15	Adam10	Anxa6
Cops3	Krt16	C3	Asl	Anxa6	Krt17	Anxa6	Apob
Cpsf7	Krt17	CD166	Asns	Apob	Mapk1	Clu	CD59
Dcps	Krt42	CD9	Atp6ap2	Atp6ap2	Muc4	Eef1e1	CD63
Eef1a1	Krt6	Coro1c	Carkd	C3	Mvp	F2	Chmp3
Eef1e1	Krt76	Epcam	CD59	CD59	Myo10	Fam175b	Clic1
Eif2s3	Ras	Hexb	Cds1	CD63	Park7	Fam213a	Clic1b
Eif3l		Hist1h2b	Comt	CD9	Plekhb2	Fdps	Cops3
Eif3m		Itga3	Eef1e1	Clu	Plxnb2	Gca	Cops4
Eif3t		Itga6	Eif2s3	Coro1c	Ppp2ca	Hist1h2b	Cops5
Eno1		Itgb3	Eif3l	Eef1e1	Prom1	Hspa1a	Cops6
Epcam		Itgb4	Eif3m	Epcam	Psmb5	Htra1	Dera
Grwd1		JH065301	Eno1	F2	Psmd13	Il24	Ehd3
H2afy		Jup	Fam175b	Fn1	Psmd6	Park7	Emb
Hexb		Krt16	Fth1	Gstp1	Psmd7	Psmb5	Fth1
Hspa1a		Mfge8	Ggt1	Hist2h2be	Ras	Serpinb6	Ftl1
JH063331		Myo10	Gstp1	Hspa1a	Rpa1	Tinagl1	Ist1
Jup		Plxnb2	H2afy	Htra1	Sdcbp	Tspan8	Lypla1
Krt17		Ptgfrn	Hexb	Il24	Sept7	Xpo2	M6pr
Mrt04		Ptprf	Idh1	Itga2	Smpdl3b		Mfi2
Park7		Pvr	Itga2	Itga3	Tacstd2		Psmd13
Polr1c		Sdcbp	JH062394	Itga6	Thbs1		Psmd6
Ptgfrn		Sep9	Lypla1	Itgb3	Thop1		Psmd7
Rpa1		Tacstd2	Nans	Itgb4	Tsnax		Reep6
Rpl7		Thbs1	Pdcl3		Tspan14		Slc44a1
Rplp2		Tinagl1	Plscr3		Tspan6		Tfrc
Smc1a		Tspan8	Rplp2		Tspan8		Tinagl1
Top2a			Smc1a		Tsta		Vps28
Tsnax			Smpdl3b				Xpo2
			Thbs1				
			Thop1				
			Top2a				

Figure S1 **Functional activities of proteins distinctly recovered in ASML-wt, -cld7kd and -cld7mP cells and TEX**. NanoLC-ESI-MS/MS proteome analysis of ASML-wt, -cld7kd and -cld7mP cells and TEX. Distinctly recovered proteins were evaluated in (A) cells and (B) TEX for molecular functions according to Panther/Reactome analysis; in (C) cells and (D) TEX the individual proteins with distinct recovery in wt versus cld7kd and cld7mP cells/TEX are grouped according to the main functional activity; (E) flow-cytometry examples of selected proteins (mean±SD of 3 replicates), significant differences to wt cells/TEX in cld7kd and cld7mP cells/TEX: *; (F) proteins enriched in ASML TEX compared to cld7kd cells were grouped for main functional activities according to (C,D).

The functional coordination of molecules overrepresented in wt cells confirmed a considerably contribution of trafficking and transporting molecules, followed by proteins engaged in transcription/translation, metabolism and signaling. Fewer differences in TEX prohibit a ranking, but in wt TEX compared to kd cells the ranking shifted towards a dominance of signaling molecules.

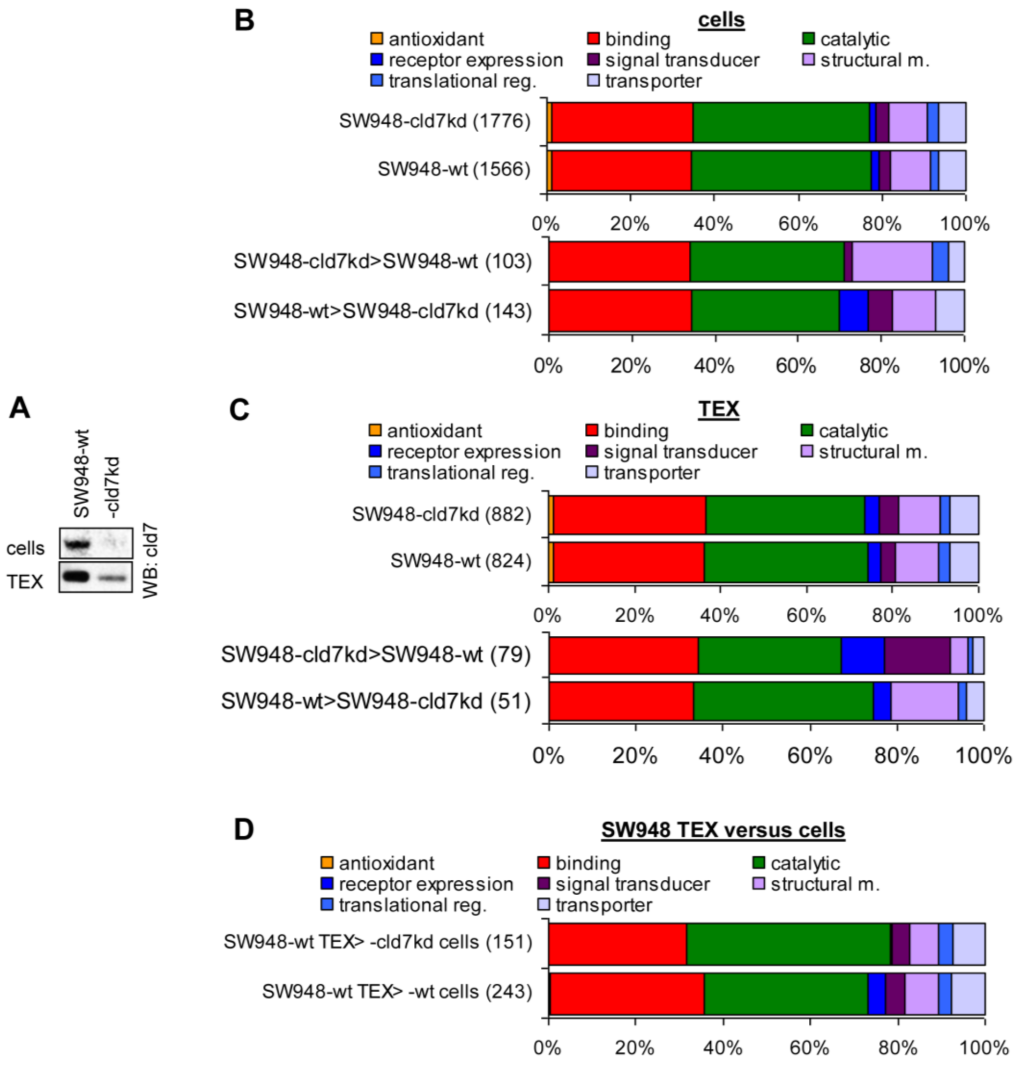
B
cells
antioxidant
binding
catalytic
receptor expression
signal transducer
structural m.
translational reg.
transporter
SW948-cld7kd (1776)
SW948-wt (1566)
SW948-cld7kd>SW948-wt (103)
SW948-wt>SW948-cld7kd (143)
0%
20%
40%
60%
80%
100%
A
SW948-wt
-cld7kd
cells
TEX
WB: cld7
C
TEX
SW948-cld7kd (882)
SW948-wt (824)
SW948-cld7kd>SW948-wt (79)
SW948-wt>SW948-cld7kd (51)
D
SW948 TEX versus cells
SW948-wt TEX> -cld7kd cells (151)
SW948-wt TEX> -wt cells (243)

E

SW948-wt cells > -cld7kd cells

transcription / translation

AKAP9
CUL4A
DDX17
DDX5
DNMT1
ELAVL1
FANCI
GARS
HNRNPA3
HNRNPC
HNRNPH2
HNRNPH3
HNRNPL
HNRNPR
HNRNPUL2
ILF2
LSM4
MDN1
MRPL17
MRPL43
NUMA1
NUP210
PABPC4
PCBP3
PNPT1
PRMT1
PUS7
RALY
RIF1
RRBP1
SFPQ
SMARCA4
SRSF1
SUPT16H
TP53BP1
TPR
UBE2L3
YBX1

structure

FLNC
JUP
LAMC1
SELENOF

adhesion / migration

CD166
CDH1
ITGA6
ITGB1
ITGB4
JUP
LAMA5
LAMB1
LAMC1
MYOC1
OLFM4
PLXNB2
SELENOF
TM9SF4

metabolisms

AKR1C1
AKR1C2
CYB5R3
DPEP1
EPHX1
ERLIN2
ERO1A
HACD3
HK2
HSD17B11
IDE
LAMB1
LBR
MDN1
MTHFG1L
MUC13
NAMPT
NUP210
OGDH
PRKCSH
PRMT1
PUS7
SEC63
TOM22
UBA6
UGT1A6
UGT1A7

signaling

AKAP9
AKR1C2
ARMC10
ARPC1B
CD166
CDH1
CIT
CTNNB1
DUSP3
EGFR
EPHB2
HACD3
HM13
HSBP1
ITGA6
ITGB1
ITGB4
ITPR3
JUP
LAMA5
MATR3
MDN1
MVP
MYH10
NAMPT
NUP210
PFN2
PLXNB2
PRKCSH
PRMT1
RRbP1
SEPT7
SFPQ
SMARCA4

proteolysis / apoptosis

ARMC10
DPEP1
HM13
HTT
IDE

trafficking / transport

AKAP9
ERLIN2
HTT
MVP
MYO1B
MYO1C
NDUFS2
NSF
OGDH
REEP6
SLC35A4
SRSF1
TM9SF4
TPR
USO1

F

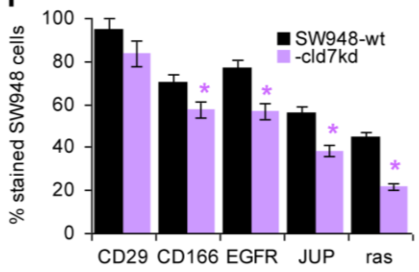

G

transcription / translation | **structure** | **adhesion / migration** | **metabolisms** | **signaling** | **proteolysis / apoptosis** | **trafficking / transport**

SW948-wt TEX > -cld7kd TEX

- transcription / translation: APEH, COPS5, H2AFY, NARS, PPP2CA, SYVC
- structure: GSN, TUBBA1A, TUBB2A
- adhesion / migration: ACTR1B, CIB1, GC, GSN, HSP4A, OLFM4, RDX, TSTA3
- metabolisms: ADA, AKR1C3, AOC1, ATP5B, GLB1, GPI, GSS, HSD17B4, NAGLU, NEU1, PAICS, PDXK, PRPSAP1, PYGL, QSOX1
- signaling: AK1C3, ANXA6, AOC1, ARPC1B, ATP6V1B2, CAPN5, CIB1, CSNKB2, EPS8L3, GLG1, GSN, IGF2R, NMT1, PSMB8, PSME1, RAB7A, RAC1, RDX, RHEB, RS27A, TUBBA1A, XPO1
- proteolysis / apoptosis: CAPN1, CAPN5, CIB1, GSN, OLFM4, PARP4, RNPEP
- trafficking / transport: AKR1C3, ANXA6, COPS5, GC, EPS8L3, IGF2R, RAB6B, RAB7A, TSTA3, TUBA1A, TUBB2A, XPO1

SW948-cld7kd TEX > SW948-wt TEX

- transcription / translation: BMP4, DYNLL1, EIF1AX, EPRS, HRSP12, PA2G4, RPL35A, SARS, VPS36
- structure: KRT14, KRT6B, MINK1, PKP3, POTEF, TLN1, TUBA1C
- adhesion / migration: CD166, CIB1, DLG1, ITGB5, PLXNB2, PTPRF
- metabolisms: ATP5B, DIP2A, G6PD, LIPH, PRSS23, PTP4A1, TMEM2, UEVLD
- signaling: ACP2, AP2A2, AP2B1, ATP1A3, BET1L, BMP4, CASK, CD166, CD276, CD97, CIB1, CTNND1, DLG1, EPHA2, EPHB4, EPRS, FARP1, FARP2, FKBP4, GNA11, GNAS, ITGB5, LY75, MAP4K4, MET, MINK1, MVB12A, MVB12B, MYOF, PLAUR, PSMC2, PTPRF, RAB6C, RAP2A, RHOG, RPA3, TLN1, TNIK, UBB
- proteolysis / apoptosis: CAPN5, CIB1, DIP2A, MINK1, PLAUR, PRSS23, SERPINB6, ST14
- trafficking / transport: AP2A2, AP2B1, ATP1A3, BET1L, CASK, DYNLL1, FKBP4, LY75, MVB12A, MVB12B, PKP3, PSMC2, RAB6C, RAP2A, TUBA1C, UEVLD, VPS36

H

SW948-wt TEX > SW948-wt & -cld7kd cells

transcription / translation

ACTL6A
COPS4
COPS5
DLG1
H2AFY
HIST1H2BB
PLSCR1
VPS25

structure

CLDN2
CLDN3
DSG2
FAT1
JAM1
JUP
TUBA1A
TUBB1

adhesion / migration

ACTRB1
ADAM9
BROX
CASK
CD63
CD9
CIB1
CLD2
DNAJA1
DSG2
EFNB1
EFNB2
EPCAM
FAT1
FPRP
GOLGA7
IGSF8
ITGA2
ITGA3
ITGA6
ITGB1
ITGB5
JAM1
JUP
LGALS3BP
MFGE8
MITD1
PARP4
PLEKHB2
PROM1
SERPINE2

metabolisms

CASK
CEMIP
CLU
DIP2B
GAA
GLB1
GNB4
ITIH2
ITIH3
LSR
MAN1A1
MELFT
MFGE8
MUC13
PLOD1
PLSCR3
PPP1R7
PTP4A1
QDPR
QPCT
QSOX1
SERINC5
SLC44A1
SMPDL38
STXBP2
TGFBI
THBS1
UXS1

signaling

A2M
ACTL6A
ADAM10
ADAM9
ANXA6
AOC1
AP2A2
APOB
ARRDC1
ATP6AP2
ATP6V1D
C3
CD55
CD63
CD81
CD82
CD9
CEMIP
CIB1
CLDN2
CLDN3
CLU
COPS5
CSNK1A1
CXADR
DLG1
DNAJA1
DPP4
EFNB1
EFNB2
EHD4
EPCAM
EPHB2
EPHB3
EPSBL3
F2
FAT1
FPRP
FYN
GNAI3
GNAS
GNB1
GNB2
GNB4
GPA33
GPRC5A
GPRC5C
HIST1H2BB
HIST1H2BC
HIST3H3
IGSF3
ITGA2
ITGA3
ITGA6
ITGAV
ITGB1
ITGB5
JAM1
JUP
LGALS3BP
LYN
MINK1
MVB12B
MVP
NCSTN
PLPP2
PLSCR1
PROM1
PSMA3
PSMB7
PSMB8
PSMD6
RAB35
RAB6B
RAP1A
RAP2A
RAP2B
RAP2C
RRAS
RSU1
S100A4
SCARB2
SDCBP
SDCBP2
SLC1A5
SLC44A2
SMPDL3B
SRC
STX4
TGBI
THBS1
TNIK
TOM1L1
TSPAN6
TSPAN8
TUBA1A
TUBB1
YES1

proteolysis / apoptosis

ADAM10
DPP4
DSG2
ITIH3
MEP1A
NCSTN
PDCD6IP
PLSC3
SERPINE2
TMPRSS4

trafficking / transport

ANXA6
AP2A2
AP2M1
APOB
ARRDC1
ATP1B1
ATP6AV0D1
ATP6AV1D
CASK
CD55
CD63
CHMP1B
CHMP2A
COPS4
COPS5
DLG1
EHD1
EHD4
F2
FA5
FTL
GNAS
GOLGA7
IST1
MVB12B
PDCD6IP
PSMA3
PSMB7
PSMB8
QDPR
RAB35
RAB6B
RALA
RAP1A
SCARB2
SDCBP
SDCBP2
SERPINE2
SLC1A5
SLC29A1
SLC2A1
SLC44A1
SLC44A2
STOM
STX12
STX3
STX4
STXBP2
TEM106B
TOM1L1
TSG101
TSPAN8
TTYH3
TUB1A1
TUBB1
VPS25
VPS28
VPS37B
VPS4B

I

SW948-wt TEX > SW948-wt cells

transcription / translation	structure	adhesion / migration	metabolisms	signaling	proteolysis / apoptosis	trafficking / transport
DHX9<br>EIF3E<br>EIF3F<br>EIF3L<br>EIF3M<br>EIF4E<br>GARS<br>HIST1H2AD<br>RPL10A<br>RPL12<br>RPL18<br>RPL6<br>RPLP0<br>RPL2<br>RPS7<br>RPSA<br>WARS		ANXA2<br>ANXA7<br>FLOT2<br>ITGB4<br>SEPT9	DPEP1<br>HBA1<br>HIST1H2AD<br>NANS<br>NT5C3A<br>PYGB<br>PYGL<br>RPL10A	DHX9<br>FLOT1<br>HISTH2AD<br>IGTB4<br>PSMB6<br>RAB22A<br>RAB5A<br>RACK1<br>RANGAP<br>SEPT9	DPEP1<br>PSMB6	ANXA2<br>ATP5L<br>COPE<br>DNAJC13<br>FLOT1<br>FLOT2<br>HBA1<br>PSMB6<br>RAB22a<br>RAB5A<br>SLC7A1<br>SYNGR2

SW948-wt TEX > SW948-cld7kd cells

transcription / translation	structure	adhesion / migration	metabolisms	signaling	proteolysis / apoptosis	trafficking / transport
PRMT1		CDH1<br>OLFM4<br>PTPRF	PYCRL<br>SLC16A3<br>TSTA3	CDH1<br>CTNNB1<br>IGF2R<br>KRAS<br>LAMTOR<br>OLFM4<br>PRMT1<br>PSMD7<br>RAB25<br>RAP1B<br>RHOB<br>TSPAN14<br>TSPAN15		ACTR1A<br>FTH1<br>IGF2R<br>LAMTOR<br>LINC7<br>PSMD7<br>RAB25<br>RAP1B<br>RDX<br>REEP6<br>RHOB<br>SLC16A3<br>TSPAN14<br>TSPAN15<br>TSTA3

J

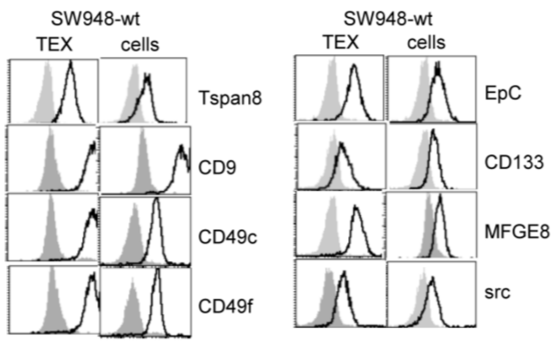

grey area: negative control, black line: antibody staining

Figure S2 **The impact of cld7 on the protein profile of colorectal cancer cells and TEX and functional activities of proteins distinctly recovered in cells and TEX**. (A) Representative WB example of cld7 expression in SW948-wt and -cld7kd cells and TEX. (B-D) NanoLC-ESI-MS/MS proteome analysis of SW948-wt and -cld7kd cells and TEX was evaluated for differences in molecular functions; (B) Panther/Reactome analysis of SW948-wt and -cld7kd cells and of proteins higher in wt or cld7kd cells; (C) Panther/Reactome analysis of SW948-wt and -cld7kd TEX and of proteins higher in wt or cld7kd TEX; (D) Panther/Reactome analysis of SW948-wt TEX versus wt cells and cld7kd cells. (E-J) Distinctly recovered proteins were clustered according to transcription/translation, structure, adhesion/migration, metabolism, signaling, proteolysis/apoptosis and trafficking/transport; (E) Proteins enriched in SW948-wt compared to -cld7kd cells; (F) Flow-cytometry examples of selected proteins (mean±SD of 3 replicates), significant differences between wt and cld7kd cells: *; (G) Proteins enriched or reduced in SW948-wt compared to -cld7kd TEX; (H) Proteins enriched in SW948-wt TEX compared to -wt and -cld7kd cells or (I) only in comparison to -wt or -cld7kd cells; (J) Flow-cytometry examples of selected proteins that expression differs significantly between TEX and cells, the overlay of antibody staining with the negative control (grey area) is shown. (E-J: full names of synonyms in Table S6).

The comparative analysis of SW948-wt versus -cld7kd cells and TEX confirms the engagement of cld7 in protein recruitment into TEX. In SW948-wt versus -cld7kd cells, differences in transcription/translation, signaling and metabolic proteins are most frequent, whereas in TEX differences in signaling and trafficking/transporting proteins become dominating. This ranking is also seen in the comparison between wt TEX versus wt and cld7kd cells, which is in line with the findings in ASML cells/TEX that cld7 strongly affects transporter and signaling molecule recruitment into TEX.

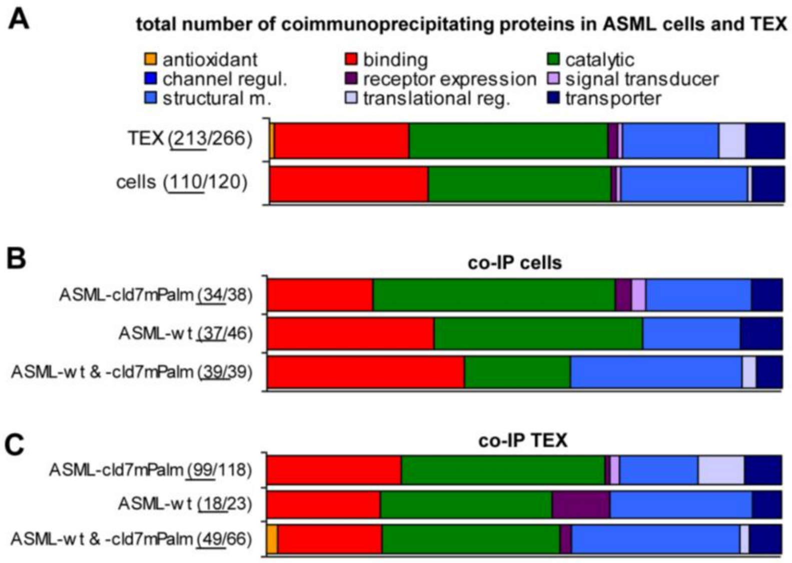

Figure S3 **Overview on molecular functions of proteins coimmunoprecipitating with claudin7 in ASML-wt and -cld7mP cells and TEX**. Lysates of ASML-wt and -cld7mP reconstituted -cld7kd cells and TEX were precipitated with anti-cld7. Precipitates were subjected to NanoLC-ESI-MS/MS proteome analysis. (A-C) The number of coimmunoprecipitating proteins and Panther/Reactome molecular function analysis of (A) cell and TEX precipitates, (B) wt versus cld7mP cells and (C) wt versus cld7mP TEX is shown.

The strong differences in the number of coimmunoprecipitating proteins in cells versus TEX confirm the impact of cld7 on protein recruitment during vesicle biogenesis. The difference in cld7- versus cld7mP-coimmunoprecipitating molecules in TEX suggests a broader range of proteins being recruited towards ILV by non-palmitoylated than palmitoylated cld7. The interpretation was supported by NanoLC-ESI-MS/MS proteome analysis of anti-EpCAM precipitates (data not shown).

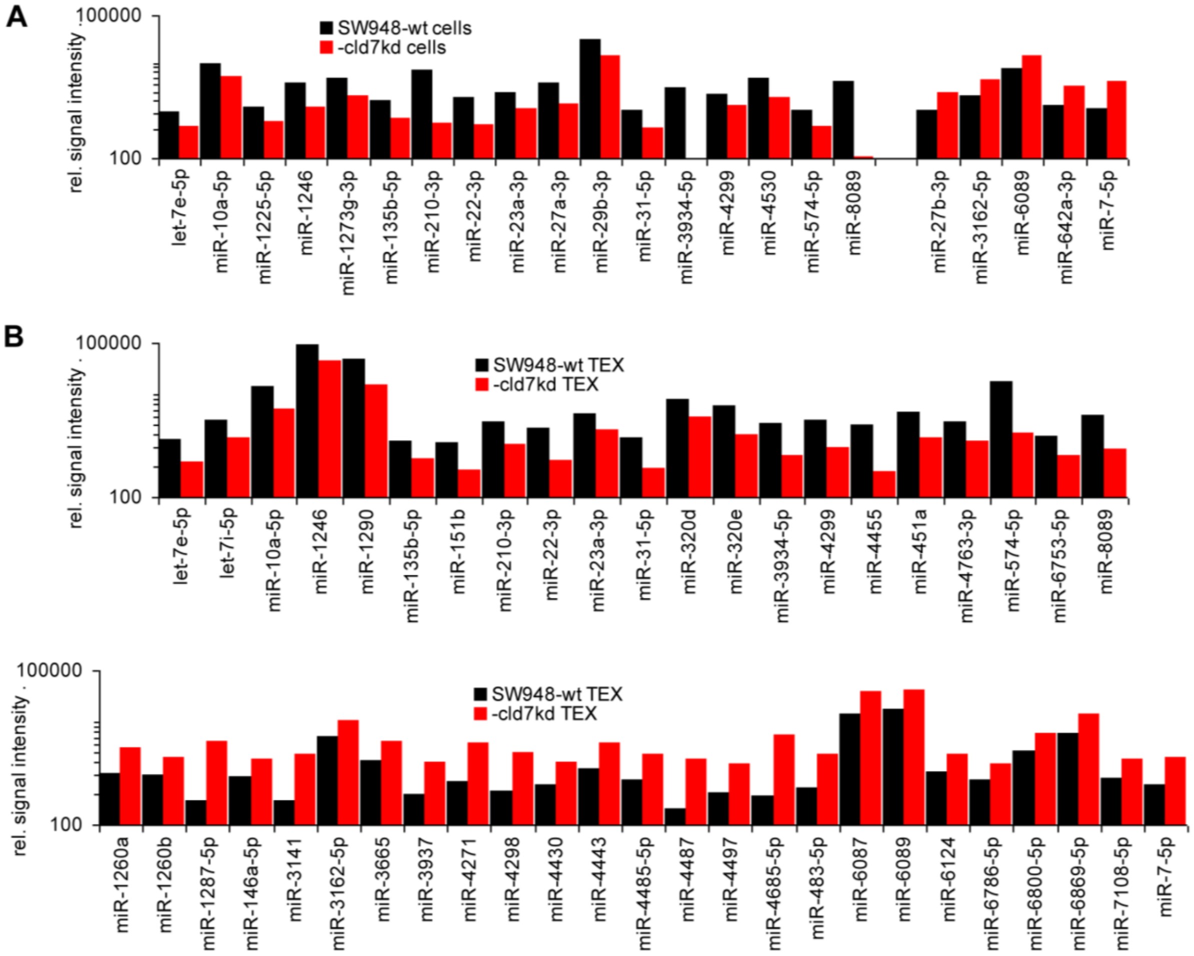
A
100000
100
rel. signal intensity
SW948-wt cells
-cld7kd cells
let-7e-5p
miR-10a-5p
miR-1225-5p
miR-1246
miR-1273g-3p
miR-135b-5p
miR-210-3p
miR-22-3p
miR-23a-3p
miR-27a-3p
miR-29b-3p
miR-31-5p
miR-3934-5p
miR-4299
miR-4530
miR-574-5p
miR-8089
miR-27b-3p
miR-3162-5p
miR-6089
miR-642a-3p
miR-7-5p
B
100000
100
rel. signal intensity
SW948-wt TEX
-cld7kd TEX
let-7e-5p
let-7i-5p
miR-10a-5p
miR-1246
miR-1290
miR-135b-5p
miR-151b
miR-210-3p
miR-22-3p
miR-23a-3p
miR-31-5p
miR-320d
miR-320e
miR-3934-5p
miR-4299
miR-4455
miR-451a
miR-4763-3p
miR-574-5p
miR-6753-5p
miR-8089
100000
100
rel. signal intensity
SW948-wt TEX
-cld7kd TEX
miR-1260a
miR-1260b
miR-1287-5p
miR-146a-5p
miR-3141
miR-3162-5p
miR-3665
miR-3937
miR-4271
miR-4298
miR-4430
miR-4443
miR-4485-5p
miR-4487
miR-4497
miR-4685-5p
miR-483-5p
miR-6087
miR-6089
miR-6124
miR-6786-5p
miR-6800-5p
miR-6869-5p
miR-7108-5p
miR-7-5p

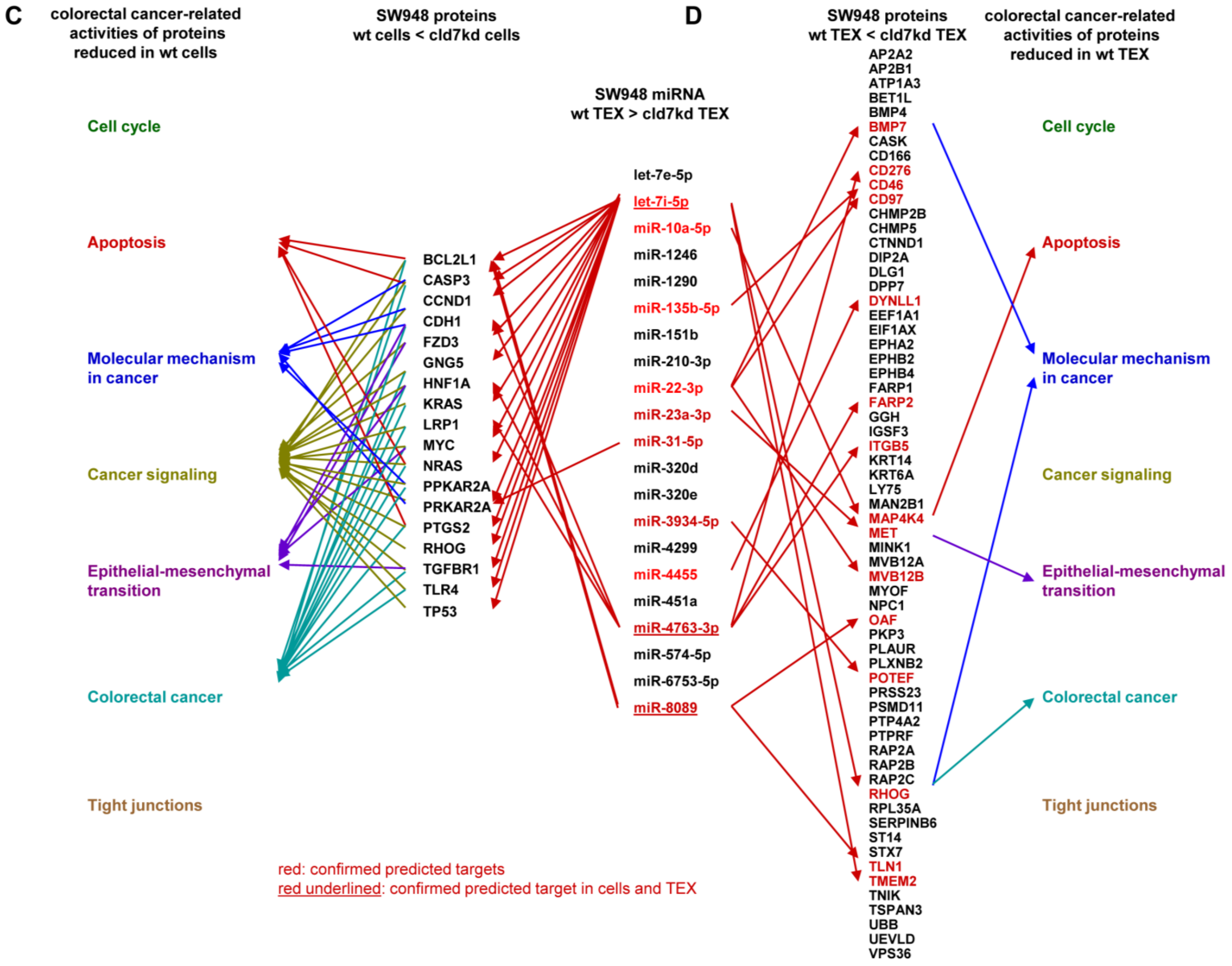
C
colorectal cancer-related activities of proteins reduced in wt cells
SW948 proteins wt cells < cld7kd cells
Cell cycle
Apoptosis
Molecular mechanism in cancer
Cancer signaling
Epithelial-mesenchymal transition
Colorectal cancer
Tight junctions
BCL2L1
CASP3
CCND1
CDH1
FZD3
GNG5
HNF1A
KRAS
LRP1
MYC
NRAS
PPKAR2A
PRKAR2A
PTGS2
RHOG
TGFBR1
TLR4
TP53
D
SW948 miRNA wt TEX > cld7kd TEX
let-7e-5p
let-7i-5p
miR-10a-5p
miR-1246
miR-1290
miR-135b-5p
miR-151b
miR-210-3p
miR-22-3p
miR-23a-3p
miR-31-5p
miR-320d
miR-320e
miR-3934-5p
miR-4299
miR-4455
miR-451a
miR-4763-3p
miR-574-5p
miR-6753-5p
miR-8089
SW948 proteins wt TEX < cld7kd TEX
AP2A2
AP2B1
ATP1A3
BET1L
BMP4
BMP7
CASK
CD166
CD276
CD46
CD97
CHMP2B
CHMP5
CTNND1
DIP2A
DLG1
DPP7
DYNLL1
EEF1A1
EIF1AX
EPHA2
EPHB2
EPHB4
FARP1
FARP2
GGH
IGSF3
ITGB5
KRT14
KRT6A
LY75
MAN2B1
MAP4K4
MET
MINK1
MVB12A
MVB12B
MYOF
NPC1
OAF
PKP3
PLAUR
PLXNB2
POTEF
PRSS23
PSMD11
PTP4A2
PTPRF
RAP2A
RAP2B
RAP2C
RHOG
RPL35A
SERPINB6
ST14
STX7
TLN1
TMEM2
TNIK
TSPAN3
UBB
UEVLD
VPS36
colorectal cancer-related activities of proteins reduced in wt TEX
Cell cycle
Apoptosis
Molecular mechanism in cancer
Cancer signaling
Epithelial-mesenchymal transition
Colorectal cancer
Tight junctions
red: confirmed predicted targets
red underlined: confirmed predicted target in cells and TEX

Figure S4 **Cld7-dependent recruitment of miRNA into colorectal cancer TEX**. MiRNA recovery in SW948-wt and -cld7kd cells and TEX was evaluated by STAR aligner version 2.5.2a. Distinct recovery (>2-fold difference in signal strength) of (A) miRNA in wt versus cld7kd cells and (B) miRNA in wt versus cld7kd TEX; (C,D) miRNA higher in wt than cld7kd TEX were evaluated for predicted targets engaged in colorectal cancer related activities (red). IPA analysis was used to assign recovery of predicted target that expression inversely correlated with the miRNA recovery. For those mRNA the engagement in colorectal cancer activity is indicated; (C) predicted targets with lower expression in wt than cld7kd cells and (D) in wt than cld7kd TEX and their engagement in CoCa-related activities are shown. (C,D: full names of synonyms in Table S6).

Several miRNA are distinctly recovered in wt versus cld7kd TEX, confirming a suggested engagement of cld7 in MVB transport to the plasma membrane. In the cell, several predicted targets of miRNA higher in wt than cld7kd TEX are accordingly repressed, all of the predicted targets being engaged in cancer-related activities, mostly in signaling in cancer. On the other hand, there is a poor correlation between miRNA and predicted target recovery in TEX, the vast majority of affected proteins being not a target of the high level recovered miRNA and only 4 are engaged in cancer-related activities. The finding suggests that miRNA recruited via cld7 into TEX does not shape the TEX protein profile.

Supplementary methods

Exosome preparation: SW948 cells were enriched for CIC by 3 rounds of spheroid growth. ASML-wt TEX can be considered as CIC-TEX, the ASML PaCa being monoclonal, not growing locally and displaying all features of a CIC [35]. CIC-enriched cells were seeded in 250ml flasks in RPMI1640 supplemented with 10% Exo-depleted FCS. After adhesion and repeated washing with PBS, the subconfluent cultures were maintained for 48h in 15ml FCS-free RPMI1640 for TEX collection. After 24h recovery (RPMI with 10% Exo-depleted FCS), TEX were collected for an additional 48h in FCS-free medium. Cell viability was not affected by the TEX collection process, being >98% before and after collection. CIC-enriched cells were discarded after TEX collection. The CIC supernatants were cleared (2x10min, 500g, 1x20min, 2000g, 1x30min, 10000g, 4°C), filtered (0.22µm) and centrifuged (Beckman Coulter ultracentrifuge, Type 45 Ti rotor, 50 ml, 120min, 100000g, 4 C). The pellet was resuspended and washed (PBS, 120min, 100000g, 4°C). The same procedure was used to deplete FCS from exosomes, collecting the supernatants. After washing, the pellet was resuspended in 0.8ml and mixed with 0.8ml 80% sucrose and layered at the bottom of 4ml ultracentrifugation tubes. The 40% sucrose was overlaid with 1.6ml 30% and 0.8ml 5% sucrose gradient and centrifuged (Beckman Coulter ultracentrifuge, SW41Ti rotor, 4ml tubes, 16h, 100000g, 4°C), collecting 12 fraction of 320µl, TEX being enriched in light density fractions 1-4 (d: 1.15-1.56g/ml). TEX preparations were either stored at -80°C or were used immediately for mass spectrometry and miRNA sequencing.

Mass spectrometry and database searches: Cell and TEX lysates and dissolved immunoprecipitates were separated by 1D SDS gel electrophoresis. After staining with Coomassie lanes were cut into ten slices. Proteins in the individual gel slices were reduced with DTT, alkylated with iodoacetamide and in-gel digested with trypsin (Promega, Mannheim, Germany) overnight. Tryptic peptides were extracted from the gel pieces, evaporated to dryness in a speed-vac concentrator and dissolved in 5µl 0.1% TFA/2.5% hexafluoro-2-propanol prior to analysis by nanoLC-ESI-MS/MS. Peptide mixtures were separated using a nanoAcquity UPLC system. For trapping we used a C18 pre-column (180µm × 20mm) with a particle size of 5µm (Waters GmbH, Eschborn, Germany). Liquid chromatography separation was performed on a BEH130 C18 main-column (100µm × 100mm) with a particle size of 1.7µm (Waters GmbH, Eschborn, Germany). Peptide mixtures were loaded on the trap column at a flow rate of 5µl/min and were eluted with a gradient at a flow rate of 400nl/min. The nanoUPLC system was coupled online to an LTQ-Orbitrap XL mass spectrometer (Thermo Scientific, Bremen, Germany). The mass spectrometer was operated in data-dependent mode to automatically measure MS1 and MS2. Data were acquired by scan cycles of one FTMS scan with a resolution of 60.000 at *m/z* 400 and a range from 300 to 2000 *m/z* in parallel with six MS/MS scans in the linear ion trap of the most abundant precursor ions. The mgf-files generated by Xcalibur software (Thermo Scientific, Bremen, Germany) were used for database searches with the MASCOT search engine (version 2.4.1, Matrix Science, London, UK) against the SwissProt database (SwissProt 2015_08, 549008 sequences; 195692017 residues) with taxonomy human (20278 sequences). Peptide mass tolerance for database searches was set to 7 ppm and fragment mass tolerance to 0.4 Da. Carbamidomethylation of C was set as fixed modification. Variable modifications included oxidation of M and deamidation of NQ. One missed cleavage site in case of incomplete trypsin hydrolysis was allowed. Furthermore, proteins were considered as identified if more than one unique peptide had an individual ion score exceeding the MASCOT identity threshold.

Real-time PCR (qRT-PCR): Real-time polymerase chain reaction (PCR) was performed using a standard TaqMan PCR kit protocol on an Applied Biosystems 7900HT Sequence Detection System (Applied Biosystems). The 10µl PCR included 0.67µl of reverse transcriptase product, 1x TaqMan Universal PCR Master Mix (Applied Biosystems), 0.2µM TaqMan probe, 1.5µM forward primer, and 0.7µM reverse primer. The reactions were incubated in a 384-well plate at 95°C for 10min, followed by 40cycles of 95°C for 15sec and 60°C for 1min. All reactions were run in triplicate. Small nuclear snRNA U6 was used as internal control for miRNA. The threshold cycle (C_T) is defined as the fractional cycle number at which the fluorescence passes the fixed threshold. TaqMan C_T values were converted into absolute copy numbers using a standard curve from synthetic lin-4 miRNA. Statistical analysis was done by the ΔC_T method [$^{\Delta}C_T$ = C_T test gene - C_T endogenous control; $^{\Delta\Delta}C_T$ = $^{\Delta}C_T$ sample - ΔC_T calibrator. For RQ (relative quantification/fold change) wt cells or TEX were used as reference.

Data accessibility: Proteome analysis are available at Functional Proteome Analysis, DKFZ, Heidelberg, Im Neuenheimer Feld 280, D69120 Heidelberg, Germany, File numbers SH2726 and SH2769, Dr. Martina Schnölzer, e-mail: m.schnoelzer@dkfz-heidelberg.de. MicroRNA microarray data are deposited at GEO (human miRNA: http://www.ncbi.nlm.nih.gov/geo/query/acc.cgi? acc=GSE119031, GSE119032, -GSE 11903, rat miRNA: http://www.ncbi.nlm.nih.gov/geo/query/ acc.cgi? acc=GSE120185).
